# Peste des petits ruminants (PPR) in Africa and Asia: A systematic review and meta‐analysis of the prevalence in sheep and goats between 1969 and 2018

**DOI:** 10.1002/vms3.300

**Published:** 2020-06-12

**Authors:** Md Ahaduzzaman

**Affiliations:** ^1^ Department of Medicine & Surgery Chattogram Veterinary & Animal Sciences University (CVASU) Chattogram Bangladesh

**Keywords:** epidemiology, infectious disease, meta‐analysis, PPR, ruminant

## Abstract

**Background:**

Peste des petits ruminants (PPR) is a prevalent viral disease of sheep and goats that impacts productivity and international animal trade. Despite the substantial economic consequences related to PPR, little is known about the prevalence of this disease at the broad geographical levels.

**Objective:**

The present study aimed to use a systematic approach to assess the regional prevalence of PPR in sheep and goats, and the associated factors that contribute to prevalence estimates.

**Methods:**

Published articles on PPR in sheep and goats were searched in PubMed, Web of Science, Scopus, Google Scholar and the reference lists of articles reporting the prevalence from 1 January 1969 to 31 December 2018. Articles were selected using inclusion and exclusion criteria. Since the heterogeneity among the studies was significant, pooled prevalences were estimated by a random effect meta‐analysis model.

**Results:**

Data on the prevalence of PPR were obtained from Africa and Asia, where the pooled prevalence estimates were 40.99% (95% CI: 37.20%–44.79%) and 38.43% (95% CI: 35.64%–41.22%) respectively. Overall, the estimated pooled prevalence at Africa‐Asia level in sheep was 39.31% (95% CI: 35.75%–42.88%) and in goats was 39.57% (95% CI: 36.66%–42.48%). Significant heterogeneity (*I* ^2^ > 80%) was noted in most pooled estimates.

**Conclusion:**

The results on the regional prevalence estimates of PPR presented here will be useful in raising awareness and advocating for Governments to engage in initiatives to eradicate PPR and prevent it from spreading to other continents.

## Graphical abstract

The present study aimed to assess the regional prevalence of Peste des petits ruminants (PPR) in sheep and goats and their associated factors that contribute in prevalence estimates using a systematic review and meta‐analysis approach. The results indicate that prevalence of PPR is high at regional context and needs of further prevalence studies in endemic regions. Results presented here will be useful in raising awareness and advocating for Governments to engage in initiatives to eradicate PPR and prevent it from spreading to other continents.

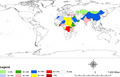

## 1 INTRODUCTION

Peste des petits ruminants (PPR) is an economically significant and widespread viral disease of ruminants that is caused by *Peste des petits ruminants virus*, a Morbillivirus that belongs to the family *Paramyxoviridae* (Gibbs, Taylor, Lawman, & Bryant, 1979). PPR spreads quickly in susceptible ruminant species, and the highest number of outbreaks occurs in sheep and goats. Cattle, camels and several wild ruminants have been infected occasionally; however, there is currently no evidence to show that the disease is maintained in these populations without concurrent infection in sheep or goats (Lembo et al., 2013). PPR is considered to be the most significant economic threat to the development of sustainable sheep and goat production across the developing world, particularly in Africa and Asia. For example, in India, the estimated annual loss caused by PPR in sheep and goats was about US$ 1,297 million per year (Singh, Bardhan, Verma, Prasad, & Sinha, 2014), while in the Turkana County of Kenya, it was US$ 19.1 million (Kihu, Gitao, et al., 2015). Moreover PPR affects national and international movement and trade of sheep and goats and their products. The disease is currently considered as one of the main transboundary and notifiable disease that constitutes an emerging or re‐emerging threat in many countries of the world. In March 2015, PPR was targeted as a high priority disease for progressive control by the World Organisation for Animal Health (OIE) and the Food and Agriculture Organisation (FAO) to eradicate the disease by 2030.

PPR is also known as sheep and goat plague. The causative agent, *Peste des petits ruminants virus* (PPRV), is considered sensitive to abiotic environmental factors, and it does not survive long outside of a host. The virus is primarily transmit through aerosol and direct contact between infected and susceptible animals (Fournié et al., 2018). The incubation period of the disease is 4–6 days, but can be up to 14 days. Clinical infection varies, and may include fever, oculo‐nasal discharges, oral erosions, pneumonia and diarrhoea (Naznin, Ahaduzzaman, Chowdhury, & Biswas, 2014). The infection period is usually 5–7 days, and death of the infected animal may occur within 10–12 days post‐infection due to severe dehydration and respiratory failure (Diallo et al., 2007). Morbidity and mortality are usually high, and PPR can create epidemics that can cause up to 100% mortality in susceptible sheep and goat populations (Parida et al., 2016). In some cases, particularly in the mild form of the disease, affected animals develop coughing and diarrhoea, and spontaneous recovery may occur within 10–15 days of infection. The magnitude of the disease depends on several factors such as the virulence of the PPR virus strain, species of animal, age, gender, breed, host immune status and previous population exposure to PPRV (Abubakar, Rasool, et al., 2016). Sheep and goats in endemic regions may develop lifelong immunity following natural infection, but naive animals may allow continuous circulation of the virus to establish an endemic situation (Mariner et al., 2016).

Since the discovery of PPR, there have been many advances in the diagnosis of PPR in sheep and goats, and diagnosis protocols range from symptomatic diagnosis to virus isolation (Banyard et al., 2010). The current diagnosis is based on clinical symptoms, pathological lesions and precise identification of PPRV antigen or antibody in various molecular or serological tests in biological samples (Balamurugan, Hemadri, Gajendragad, Singh, & Rahman, 2014). Virus isolation is the gold‐standard for the diagnosis of PPR, but this is mostly impractical in the field (Banyard et al., 2010). As a morbillivirus, PPRV is antigenically similar to the viruses that cause rinderpest in cattle, measles in humans and distemper in dogs, but can be serologically distinguished by use of commercially available enzyme‐linked immunoassay (ELISA) kits (Anderson & McKay, 1994). In many countries, diagnosticians are moving towards the use of molecular techniques such as polymerase chain reaction (PCR) for early and specific diagnosis of PPR in sheep and goats (Kgotlele, Kasanga, Kusiluka, & Misinzo, 2014). Adaptation of a method of diagnosis often depends on local facilities and availability of resources (Banyard et al., 2010).

The first report of PPR was made in the Ivory Coast, West Africa, in 1942. Today, PPR is quite common in both Africa and parts of Asia, and is emerging as a threat to other continents such as Europe (Parida et al., 2016). In recent decades, several scholarly narrative reviews of the distribution of PPRV lineages based on nucleoprotein and fusion genes sequence analysis for particular geographical regions have been published (Banyard et al., 2010; Dilli, Geidam, & Egwu, 2011; Parida et al., 2016). A comprehensive pooled prevalence estimate of PPR has not been reported, but a wide range of prevalence estimates of PPR in sheep and goats have been reported in various regions (Cêtre‐Sossah et al., 2016; Jaisree et al., 2017; Li et al., 2017). Reasons for the inconsistent prevalence estimates of PPR could include an endemic situation of PPRV in a particular geographic area, differences in methods used for identifying the disease, origin of samples, sampling strategy, year of study, study duration and species of animal studied. An overview of knowledge on the regional prevalence of PPR in sheep and goats will offer a better understanding of the distribution of the disease and its impacts on animal production, and will be useful in disease control. This study aims to use a systematic review and meta‐analysis approach to estimate the regional prevalence of PPR in sheep and goats, and to evaluate the potential factors that contribute to the variability in the prevalence and distribution of disease.

## 2 METHODS

The study was conducted following PRISMA (Preferred Reporting Items for Systematic Reviews and Meta‐Analyses) guidelines for systemic review and meta‐analysis. The PRISMA 2009 checklist was used to ensure the inclusion of relevant information and maintain study standard (Appendix S1, Table S1).

### 2.1 Search strategy

An optimized systemic search strategy was used to identify all published studies related to the prevalence of PPR in sheep and goats. Published works were searched in four electronic web search engines: PubMed, Web of Science, Scopus and Google Scholar for articles, published between January 1969 and December 2018. The search was conducted on 25 February 2019. The search syntaxes were used to search the literature with the following keywords: (Prevalence OR Incidence OR Frequency OR Detection OR Occurrence OR Identification OR Isolation OR Characterization OR Investigation OR Survey OR Rate) AND (PPR OR Peste des petits ruminants OR Goat plague OR Kata OR ovine rinderpest OR Caprine rinderpest) AND (Goat OR Doe OR Buck OR Caprine OR Ovine OR Sheep OR Ram OR Ewe OR small ruminant). Search field option was selected as “All fields”. A restriction was placed on the language of publication “English”. Search terms and keywords were adjusted according to minor differences in syntax rules of four electronic databases. The reference management software EndNote X8 (Clarivate Analytics, Philadelphia, PA) was used to organize and remove duplicate articles between the search engines. The reference lists of extracted articles were also searched manually in triplicate for additional potential articles and to ensure that selected databases searches did not miss any reports.

### 2.2 Selection of studies

Articles were selected for meta‐analysis based on the following criteria: published between January 1969 and December 2018; full‐text article; published in English; any country of the world; studied population is sheep or goat or both; reported as animal level prevalence data; cross‐sectional, case‐control, longitudinal and cohort studies. Reasons for exclusion of articles were species other than sheep or goat, prevalence data not reported, case study or retro‐prospective study, flock with a history of vaccination, comparison of methods, experimental trial and articles in languages other than English.

### 2.3 Quality of the studies

Studies selected for this meta‐analysis were assessed for quality of reporting and selection for bias using a quality assessment checklist (Ahaduzzaman, 2019; Hoy et al., 2012). The checklist included nine parameters which have “yes” and “no” applicable option. Operationally, the “yes” answer was awarded a score of 1; while “no” was provided with 0 scores. Ultimately, for each article, the mean score was determined. Articles were categorised as follows: low quality = 0−3; moderate quality = 4−6 and high quality = 7−9 (Appendix S2, Texts S1, Figure S1).

### 2.4 Data extraction

The following data were extracted from each eligible study on a spreadsheet where possible: author, year, country, continent, study duration, species of animal, origin of sample, diagnostic technique, population size and number of positive samples. Overall, data from 243,864 animals (Sheep: 87,580 and goats: 156,284) from various geographical locations were analysed (Tables 1 and 2).

**TABLE 1:** Estimated pooled prevalence of PPR in sheep by world region

World region	No. of study	No. of sheep sampled	No. of positive sheep	Pooled estimate %	95% CI	Heterogeneity chi‐squared (χ2)	*l* ^2^%	*p*‐value
Africa‐Asia estimate	136	87,580	30,935	39.31	35.75–42.88	67,409.94	99.8	<.0001
Africa	66	42,694	15,428	40.16	34.59–45.73	45,254.77	99.9	<.0001
Asia	70	44,886	15,507	38.63	33.73–43.53	21,120.37	99.7	<.0001

Note

No record of goat PPR from other continents.

Abbreviations: CI, confidence interval; *I* ^2^, inverse variance index; χ2, Cochran's Q chi‐square.

**TABLE 2:** Estimated pooled prevalence of PPR in goats by world region

World region	No. of study	No. of goat sampled	No. of positive goat	Pooled estimate %	95% CI	Heterogeneity chi‐squared (χ2)	*l* ^2^%	*p*‐value
Africa‐Asia estimate	192	156,284	47,278	39.57	36.66–42.48	63,814.71	99.7	<.0001
Africa	70	42,128	13,992	41.79	36.34–47.23	20,821.71	99.7	<.0001
Asia	122	114,156	33,286	38.34	34.79–41.89	42,822.59	99.7	<.0001

Note

No record of goat PPR from other continents.

Abbreviations: CI, confidence interval; *I* ^2^: inverse variance index; χ2, Cochran's Q chi‐square.

### 2.5 Data analysis

All extracted data were transcribed and stored in a Microsoft Excel spreadsheet. Crude prevalence estimation was done by the number of positive animals divided by the total number of animals sampled and expressed as a percentage. Only the crude estimate of prevalence was used throughout, and the 95% confidence interval (CI). The 95% CI of the mean was calculated using the standard formula for a proportion (p): $p\;\pm \;1.96\sqrt{\left[p\;\;\times \;\;(100-p)÷n\right]}$, where n is the studied population size. In the event that the higher limit of CI exceeded 100, then the value was settled to 100 to avoid >100% prevalence, or when the lower limit was lower than the positive value, then the value was settled to 0 to avoid negative prevalence. Data were analysed using the “metan” command of the STATA v.11.0 software (StataCorp LP, College Station, TX, USA). Heterogeneity across studies was assessed by chi‐square test on Cochran"s *Q* statistic (represented as *χ* ^2^ and *p*‐values) (Higgins & Thompson, 2002), which was interpreted by *I* ^2^ statistic value, considering the *I* ^2^ values of 25%, 50% and 75% as low, moderate and high heterogeneity respectively (Higgins & Thompson, 2002). Owing to the nature of the studies, there was substantial heterogeneity between studies; therefore, random effect meta‐analysis was used for summary statistics. Subgroup analysis was also conducted by world region, age, sex, origin of sample, methods of detection and study duration. Publication bias was assessed by Egger's test using two funnel plots, and the sources of funnel plots asymmetry were also tested to identify small study effects (Egger, Smith, Schneider, & Minder, 1997).

## 3 RESULTS

### 3.1 Search results and eligible studies

**FIGURE 1:**
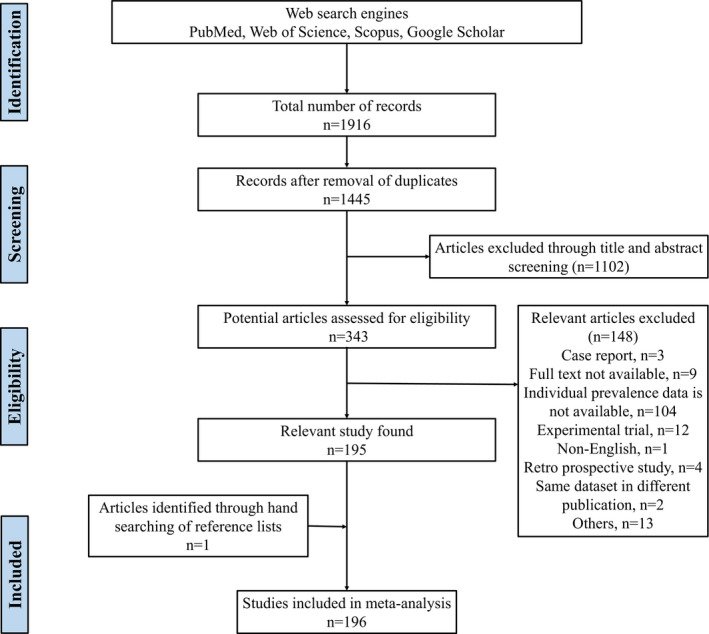
Flow diagram of the selection of eligible studies for inclusion in the meta‐analysis

Figure 1 shows the search results. The electronic search on selected search engines identified 1916 articles. After removal of duplicates and screening of titles and abstracts, a total of 343 eligible articles were found, of which 148 articles were excluded due to following reasons: case report (*n* = 3); full‐text not available (*n* = 9); individual prevalence data not available (*n* = 104); experimental trial (*n* = 12); article in a language other than English (*n* = 1); retro prospective study (*n* = 4); same dataset in different publication (*n* = 2) and others (*n* = 13). The list of excluded articles together with the causes for their exclusion is provided in Appendix S3, Texts S2. A total of 196 eligible articles were used for systemic review and meta‐analysis (Abdalla, Majok, El Malik, & Ali, 2012; Abraham et al., 2005; Abubakar, Ali, & Khan, 2008; Abubakar, Jamal, Arshed, Hussain, & Ali, 2009; Abubakar, Jamal, Hussain, & Ali, 2008; Abubakar, Jamal, Khan, & Ali, 2008; Abubakar, Javed Arshed, Hussain, & Ali, 2011; Abubakar, Manzoor, et al., 2016; Abubakar, Rasool, et al., 2016; Abubakar, Zahur, Afzal, Ali, & Gonzales, 2017; Abubakar, Zahur, Naeem, Khan, & Qureshi, 2018; Acharya, Poudel, & Acharya, 2018; Adel, Abu‐Elzein, Al‐Naeem, & Amin, 2004; Adombi et al., 2017; Afera, Hussien, & Amsalu, 2014; Ahmad, Jamal, Ali, & Hussain, 2005; Ahmed et al., 2017; Ahmed, Rahman, Alam, Paul, & Uddin, 2016; Alam et al., 2018; Albayrak & Alkan, 2009; Albayrak & Gür, 2010; Al‐Dubaib, 2008, 2009; Ali, Intisar, & Khalafalla, 2014; Al‐Majali, Hussain, Amarin, & Majok, 2008; Almeshay et al., 2017; Ameen & Ajayi, 2013; Amin, 2015; Anees et al., 2013; Atta‐ur‐Rahman, Rahman, Akhtar, & Ullah, 2004; Ayim‐Akonor, Obese, Arthur, Owusu‐Ntumy, & Otsyina, 2014; Aytekin, Mamak, Ulucan, & Kalınbacak, 2011; Baazizi, Ait‐Oudhia, Parida, Mahapatra, & Khelef, 2015; Baazizi, Khelef, & Hussain, 2017; Balamurugan, Das, et al., 2014; Balamurugan, Krishnamoorthy, et al., 2014; Balamurugan et al., 2011; Balamurugan, Saravanan, et al., 2012; Banik, Podder, Samad, & Islam, 2008; Bari et al., 2018; Begum et al., 2016, 2017; Bello et al., 2016, 2018; Bhanuprakash et al., 2008; Bhaskar, Deshmukh, Chopade, Rautmare, & Aziz, 2011; Birindwa, George, Ntagereka, Christopher, & Lilly, 2017; Bupasha, Hossain, Sarker, Ahaduzzaman, & Biswas, 2015; Cêtre‐Sossah et al., 2016; Chauhan et al., 2012, 2014; Chavan, Digraskar, Dhonde, & Bedarkar, 2009; Choudhary, Jhala, & Kanani, 2009; Chowdhury, Bhuiyan, Rahman, Siddique, & Islam, 2014; Das, Shil, & Islam, 2007; De et al., 2016; Delil, Asfaw, & Gebreegziabher, 2012; Devi, Das, Sharma, & Dutta, 2016; Durrani, Kamal, Mehmood, & Shakoori, 2010; Elhaig, Selim, Mandour, Schulz, & Hoffmann, 2018; El‐Rahim, Baky, Habashi, Mahmoud, & Al‐Mujalii, 2005; El‐Rahim, Sharawi, Barakat, & El‐Nahas, 2010; El‐Yuguda, Abubakar, Nabi, Andrew, & Baba, 2009; El‐Yuguda, Chabiri, Adamu, & Baba, 2010; El‐Yuguda, Saheed Baba, Ganiyu Ambali, & Egwu, 2013; Enan et al., 2013; Ezeokoli, Umoh, Chineme, Isitor, & Gyang, 1986; Faris, Yilkal, Berhe, & Kelay, 2012; Farougou, Gagara, & Mensah, 2013; Fentie et al., 2018; Gari et al., 2015; Gari, Serda, Negesa, Lemma, & Asgedom, 2017; Goossens et al., 1998; Güler, Şevik, & Hasöksüz, 2014; Gurcay, Kizil, & Baydar, 2013; Haq et al., 2017; Haque, Habib, Islam, Khan, & Hannan, 2004; Haroun, Hajer, Mukhtar, & Ali, 2002; Hota et al., 2018; Intisar et al., 2017; Ishag, Intisar, & Ali, 2014; Ishag, Saeed, & Ali, 2015; Islam et al., 2014, 2015, 2016, 2017; Islam, Kamal, & Ali, 2018; Islam, Khan, Kader, Begum, & Asgar, 2012; Jaisree et al., 2017; Jalees, Hussain, Arshad, Mohammad, & Khan, 2016; Jalees et al., 2013; Janus, Tresamol, Saseendranath, Vijayakumar, & Pillai, 2009; Kabir, Hossain, Ershaduzzaman, Yousuf, & Islam, 2016; Kabir et al., 2010; Karam et al., 2018; Kardjadj, Ben‐Mahdi, & Luka, 2015; Karlewad, Bhikane, Ambore, & Awaz, 2007; Kgotlele, Kasanga, et al., 2014; Kgotlele, Macha, et al., 2014; Kgotlele, Torsson, Kasanga, Wensman, & Misinzo, 2016; Khan, Siddique, Abubakar, Arshad, & Hussain, 2008; Khan, Siddique, Arshad, Khan, & Rehman, 2007; Khaskheli et al., 2017; Kihu,Gachohi, et al., 2015; Krishna, Rao, & Shaila, 2001; Kumar, Sinha, Roy, Kumari, & Kumar, 2017; Kwiatek et al., 2011; Lawal, Lasisi, Emikpe, & Ogundipe, 2011; Li et al., 2017; Lucky et al., 2016; Lundervold et al., 2004; Luther et al., 2005; Maganga et al., 2013; Mahajan, Agrawal, Kumar, Mohan, & Pande, 2012, 2013; Mahamat et al., 2018; Mahapatra et al., 2015; Mahmoud, Abdellatif, & Abdalla, 2017; Mahmoud, Abdellatif, & Shazali, 2016; Mahmoud, Elbayoumy, Sedky, & Ahmed, 2017; Mahmoud & Galbat, 2017; Maitlo et al., 2017; Mbyuzi, Komba, Kimera, & Kambarage, 2014; Mebrahtu, Getachew, Tesfaye, Sahlu, & Aragaw, 2018; Megersa et al., 2011; Meher, Afrin, Hassan, & Alam, 2017; Mehmood, Ali, Gadahi, Malik, & Shah, 2009; Milind et al., 2018; Mohanto, Hoque, & Juli, 2018; Mostafa, 2012; Moumin, Moussa, Teshale, & Gezahegne, 2018; Muhsen, 2013; Mulindwa et al., 2011; Munir, Shah, Shabbir, & Berg, 2013; Munir, Siddique, Shehzad, Zohari, & Stahl, 2010; Muse et al., 2012; Nabi, Hossain, Saha, Alam, & Giasuddin, 2018; Nath et al., 2014; Naznin et al., 2014; Nizamani et al., 2015; Nwobodo, Ezeifeka, Ezejiofor, & Onyianta, 2013; Opasina, 1985; Opasina & Putt, 1985; Oshiek et al., 2018; Osman, Ibrahim, Osman, Alnour, & Eldin, 2018; Otsyina, Arthur, Ayim‐Akunnor, & Obese, 2013; Özkul et al., 2002; Ozmen, Kale, Haligur, & Yavru, 2009; Parvez, Khatun, & Al Noman, 2014; Patil et al., 2009; Poddar, Tuli, Sultana, Akter, & Alauddin, 2018; Raghavendra et al., 2008; Rahman, Alam, Alam, Hasan, & Moonmoon, 2016; Rahman et al., 2018; Rahman, Hassan, Sultana, Uddin, & Hossain, 2017; Rahman, Hossain, Ahsan, Khokon, & Kibria, 2011; Rahman, Shadmin, et al., 2011; Rakshit et al., 2015; Rashid, Asim, & Hussain, 2008; Rony, Rahman, Alam, Dhand, & Ward, 2017; Saeed, Abdel‐Aziz, & Gumaa, 2018; Saeed, Ali, Khalafalla, & Rahman‐Mahasin, 2010; Sağlam & Temur, 2009; Salih, Elfadil, Saeed, & Ali, 2014; Sande et al., 2011; Sannat, Chandel, Chauhan, & Dadawala, 2011; Saravanan et al., 2007; Saritha, Shobhamani, Rajak, & Sreedevi, 2015; Saritha, Shobhamani, & Sreedevi, 2014; Sarker & Islam, 2011; Şevik & Sait, 2015; Sharma, Mehta, Prakash, & Shukla, 2012; Sharma, Shrivastava, Mehta, & Shukla, 2012; Shukla, Singh, & Hirpurkar, 2008; Siddiqui et al., 2014; Singh, Malik, Sharma, & Kuldeep, 2015; Singh, Saravanan, Sreenivasa, Singh, & Bandyopadhyay, 2004; Singh, Jindal, Nain, & Khokhar, 2006; Soltan & Abd‐Eldaim, 2014; Sundufu et al., 2015; Swai et al., 2009; Taylor, Al Busaidy, & Barrett, 1990; Thombare & Sinha, 2009; Torsson et al., 2017; Undrakhbayar, Uuganbayar, & Odbileg, 2016; Wang et al., 2009; Waret‐Szkuta et al., 2008; Yapici et al., 2014; Yener, Sağlam, Temur, & Keleş, 2004; Yilmaz, 2016; Yousuf, Giasuddin, Islam, & Islam, 2015; Yousuf et al., 2017; Zahur et al., 2008, 2009, 2011, 2014) (Appendix S4, Texts S3). Among the selected articles, 115 articles reported the prevalence of PPR in both sheep and goats, 11 articles in sheep and 70 articles in goats. In relation to the origin of samples, 4 studies were from abattoirs, 32 from farms, 17 from free range flocks, 31 from hospitals, 29 from household flocks, 1 from both hospital and household and 42 from mixed flocks; for 40 studies, the origin was not mentioned. Based on the method of diagnosis, 19 studies used PCR, 128 used serology, 40 used symptoms and 9 used combined or other methods. An overview of the characteristics of each included study is supplied (Appendix S4, Texts S4).

### 3.2 Continents and countries

All articles included in this study represent data from two continents (Africa and Asia), covering 34 countries of the world. The highest number of articles (*n* = 129) were from Asia covering 12 countries: Bangladesh (*n* = 37), China (*n* = 2), India (*n* = 38), Iraq (*n* = 1), Kazakhstan (*n* = 1), Kyrgyzstan (*n* = 1), Mongolia (*n* = 1), Nepal (*n* = 1), Oman (*n* = 1), Pakistan (*n* = 28), Saudi Arabia (*n* = 7) and Turkey (*n* = 11). Articles (*n* = 67) from Africa covered 22 countries: Algeria (*n* = 3), Benin (*n* = 1), Chad (*n* = 1), Comoros (*n* = 1), Congo (*n* = 1), Djibouti (*n* = 1), Egypt (*n* = 4), Eritrea (*n* = 1), Ethiopia (*n* = 10), Gabon (*n* = 1), Gambia (*n* = 1), Ghana (*n* = 2), Jordan (*n* = 1), Kenya (*n* = 1), Libya (*n* = 1), Morocco (*n* = 1), Niger (*n* = 1), Nigeria (*n* = 11), Sierra Leone (*n* = 1), Sudan (*n* = 13), Tanzania (*n* = 8) and Uganda (*n* = 2).

### 3.3 Prevalence estimates

**FIGURE 2:**
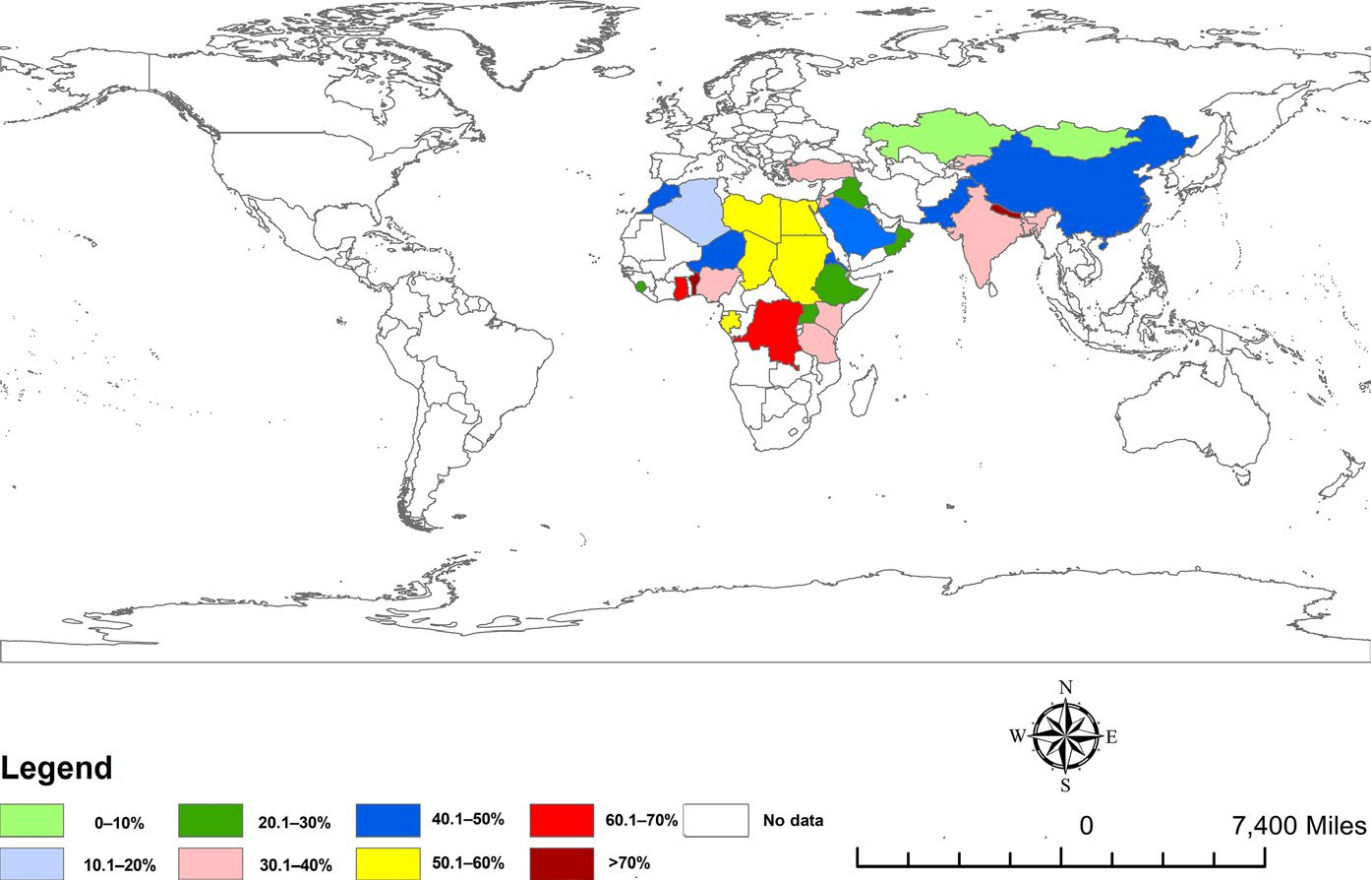
Estimated prevalence of Peste des petits ruminants (PPR) in sheep and goats in different countries of the world from 1969 to 2018. The prevalence estimate is based on a meta‐analysis of 196 studies comprising 2, 43,864 sheep and goats. The map was produced using ArcGIS v.10.3.1 (Esri, Redlands, CA, USA)

**FIGURE 3:**
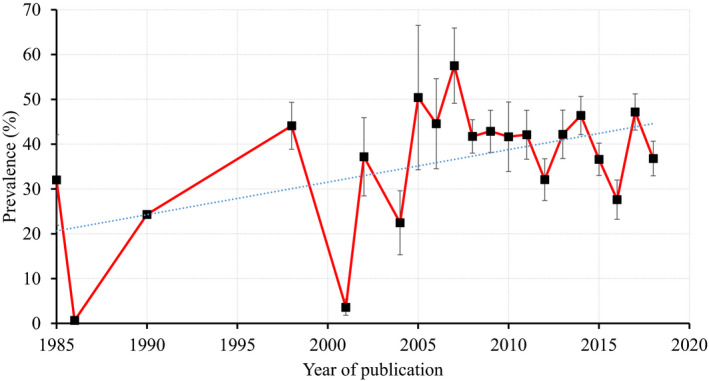
Trend in the pooled prevalence (LSM ± *SE*) of Peste des petits ruminants (PPR) in sheep and goats based on 196 studies within the range of this meta‐analysis (1969 to 2018) (*p* = .0003). No prevalence data found between 1969 and 1984

The random effect meta‐analysis showed that the pooled prevalence of PPR in sheep ranged from 38.63 (95% CI: 33.73%–43.53%) to 40.16 (95% CI: 34.59%–45.73%), with an overall random pooled prevalence of 39.31 (95% CI: 35.75%–42.88%) with considerable heterogeneity (*I* ^2^ = 99.8%, *p* < .0001) (Table 1). Likewise, the pooled prevalence of PPR in goats ranged from 38.34 (95% CI: 34.79%–41.89%) to 41.79 (95% CI: 36.34%–47.23%), with an overall random pooled prevalence of 39.57 (95% CI: 36.66%–42.48%) with considerable heterogeneity (*I* ^2^ = 99.7%, *p* < .0001) (Table 2). Overall, the Africa‐Asia pooled estimated prevalence of PPR in sheep and goats was 39.46% (95% CI: 37.23%–41.69%) with substantial heterogeneity (*I* ^2^ = 99.8%, *p* < .0001) (Table 3). The estimated pooled prevalence of PPR in sheep and goats by country is shown in Figure 2 and Table 4. The pooled prevalence of PPR in sheep and goats by publication year is presented in Figure 3.

**TABLE 3:** Pooled prevalences and estimated sources of heterogeneity in the prevalence of PPR in sheep and goats

Variables	Population	Pooled estimate prevalence (%)	95% CI	Heterogeneity chi‐squared (χ2)	*l* ^2^%	*p*‐value
World region						
Africa‐Asia estimate	243,864	39.46	37.23–41.69	130,000	99.8	<.0001
Africa	84,822	40.99	37.20–44.79	68,670.75	99.8	<.0001
Asia	159,042	38.43	35.64–41.22	64,331.34	99.7	<.0001
Age						
Young (≤1 year)	25,059	35.99	31.12–40.87	6,235.86	99.3	<.0001
Adult (>1 year)	26,879	39.91	33.70–46.12	10,675.58	99.5	<.0001
Sex						
Male	11,171	35.14	30.28–40.01	1929.90	97.5	<.0001
Female	23,168	41.00	35.09–46.91	5,307.65	99.1	<.0001
Origin of sample						
Abattoir	2,192	42.72	37.97–47.47	16.86	70.3	<.0001
Farm	25,305	42.70	36.40–49.01	16,225.88	99.7	<.0001
Free range flocks	9,488	39.33	32.32–46.34	5,827.96	99.4	<.0001
Hospital	42,123	30.15	26.95–33.36	7,532.16	99.5	<.0001
House hold flocks	19,204	37.38	32.22–46.53	24,957.71	99.8	<.0001
Mixed flocks	83,976	40.76	36.43–45.09	15,715.13	99.5	<.0001
Methods of detection						
IHC	362	32.32	7.76–56.87	49.15	95.9	<.0001
PCR	2,453	48.03	36.32–59.74	1,476.29	98.0	<.0001
Serology	163,886	40.13	37.45–42.81	84,175.68	99.7	<.0001
Symptomatic	77,163	33.30	28.06–38.55	45,212.97	99.9	<.0001
Study duration						
≤6	38,327	41.47	36.09–46.85	31,834.92	99.7	<.0001
>6 to ≤12	74,100	31.39	28.37–34.42	16,186.05	99.6	<.0001
>12	97,644	38.64	35.92–45.07	63,650.34	99.6	<.0001

Abbreviations: CI, confidence interval; *I* ^2^: inverse variance index; χ2, Cochran's Q chi‐square.

**TABLE 4:** Estimated pooled prevalence of PPR in sheep and goats in different countries

Country	Population	Pooled estimate prevalence (%)	95% CI	Heterogeneity (χ2)	*I* ^2^%	*p*‐value
Algeria	7,440	19.29	15.62–22.96	93.41	90.4	<.0001
Bangladesh	41,418	31.02	26.60–35.45	7,945.5	99.5	<.0001
Benin	19	84.15	70.17–98.13	0.01	0	.931
Chad	3,546	52.73	44.10–61.35	27.68	96.4	<.0001
China	1632	42.93	0–88.72	1503.58	99.9	<.0001
Comoros	1,048	2.37	1.43–3.31	0	—	—
Congo	150	64.67	57.02–72.32	0	—	—
Djibouti	1516	4.79	0.70–8.87	12.58	92	<.0001
Egypt	2,776	53.26	38.82–67.71	650.49	98.6	<.0001
Eritrea	32	44.19	15.23–73.15	1.83	45.3	.176
Ethiopia	16,958	25.6	21.56–29.65	4,404.54	99.5	<.0001
Gabon	106	59.22	0–100	59.3	98.3	<.0001
Gambia	1686	44.1	33.80–54.40	14.53	93.1	<.0001
Ghana	3,269	69.97	47.13–92.82	528.46	99.4	<.0001
India	43,838	39.7	33.73–45.68	15,824.5	99.6	<.0001
Iraq	1,175	27.66	25.10–30.22	0	—	—
Jordan	1,329	38.93	19.39–58.47	46.94	97.9	<.0001
Kazakhstan	679	0.59	0.07–1.11	0.08	0	.774
Kenya	969	35.7	27.63–43.76	7.16	86	.007
Kyrgyzstan	655	35.11	31.46–38.77	0	—	—
Libya	721	51.18	36.60–65.75	9.17	89.1	.002
Mongolia	1950	0.81	0.41–1.21	0.18	0	.674
Morocco	36	44.44	28.21–60.68	0	—	—
Nepal	460	82.61	79.15–86.08	0	—	—
Niger	519	44.45	38.70–50.21	1.82	45	.178
Nigeria	12,950	39.29	28.75–49.83	14,974.08	99.9	<.0001
Oman	724	24.31	21.18–27.43	0.04	0	.846
Pakistan	56,984	43.55	38–22–48.88	8,936.32	99.5	<.0001
Saudi Arabia	6,743	49.97	34.32–65.62	5,976.52	99.8	<.0001
Sierra Leone	5,679	29.04	27.86–30.22	0	—	—
Sudan	16,832	53.97	46.45–61.49	1,890.76	98.8	<.0001
Tanzania	6,487	38.97	32.20–45.73	257.96	95.7	<.0001
Turkey	2,784	34.29	25.15–43.43	342.16	96.2	<.0001
Uganda	754	26.06	0–54–25	194.05	99	<.0001

Note

Not estimated due to having single study.

**FIGURE 4:**
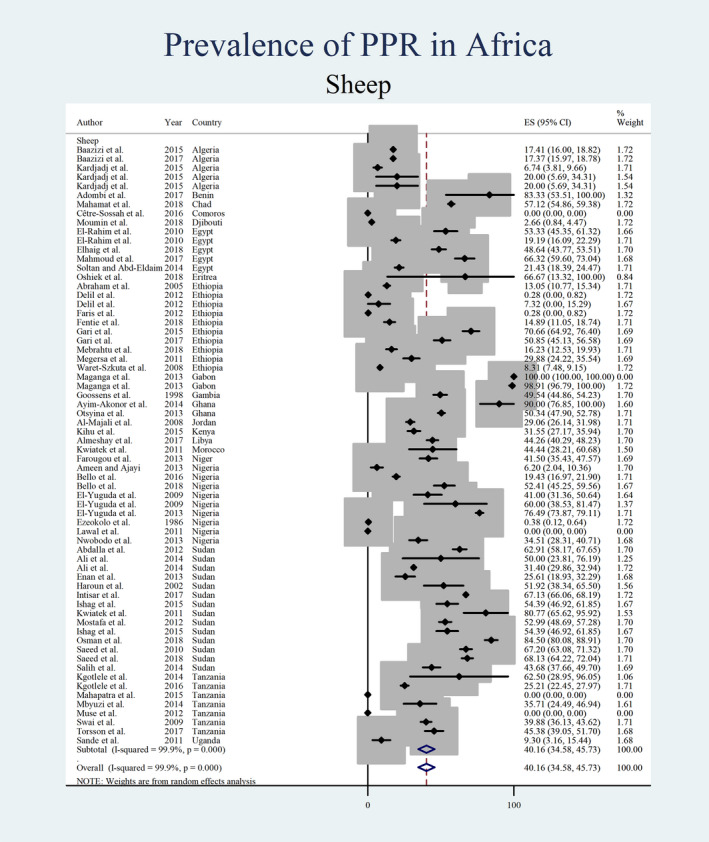
Forest plot of the prevalence estimates of Peste des petits ruminants (PPR) in sheep amongst studies conducted in Africa

**FIGURE 5:**
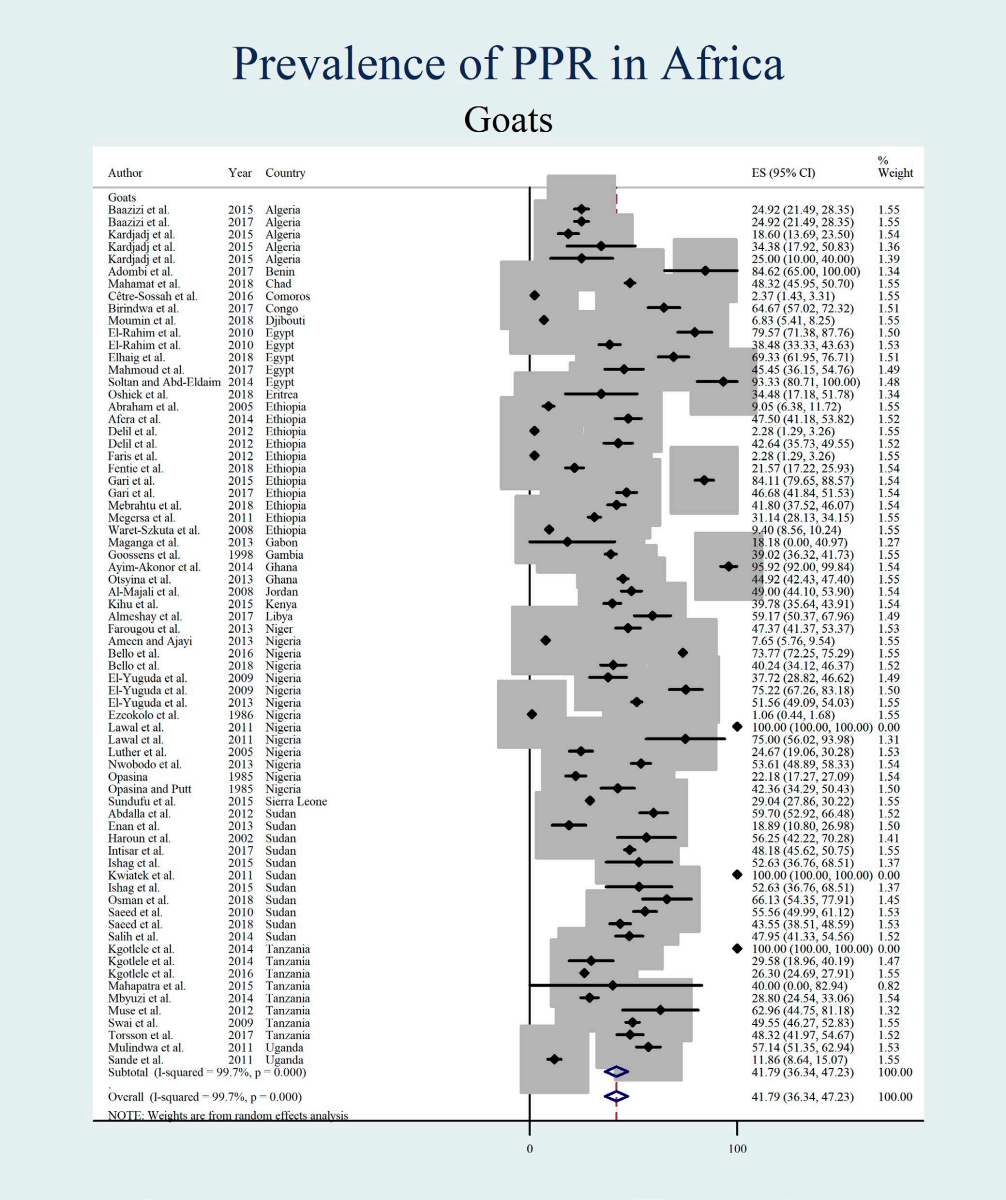
Forest plot of the prevalence estimates of Peste des petits ruminants (PPR) in goats amongst studies conducted in Africa

**FIGURE 6:**
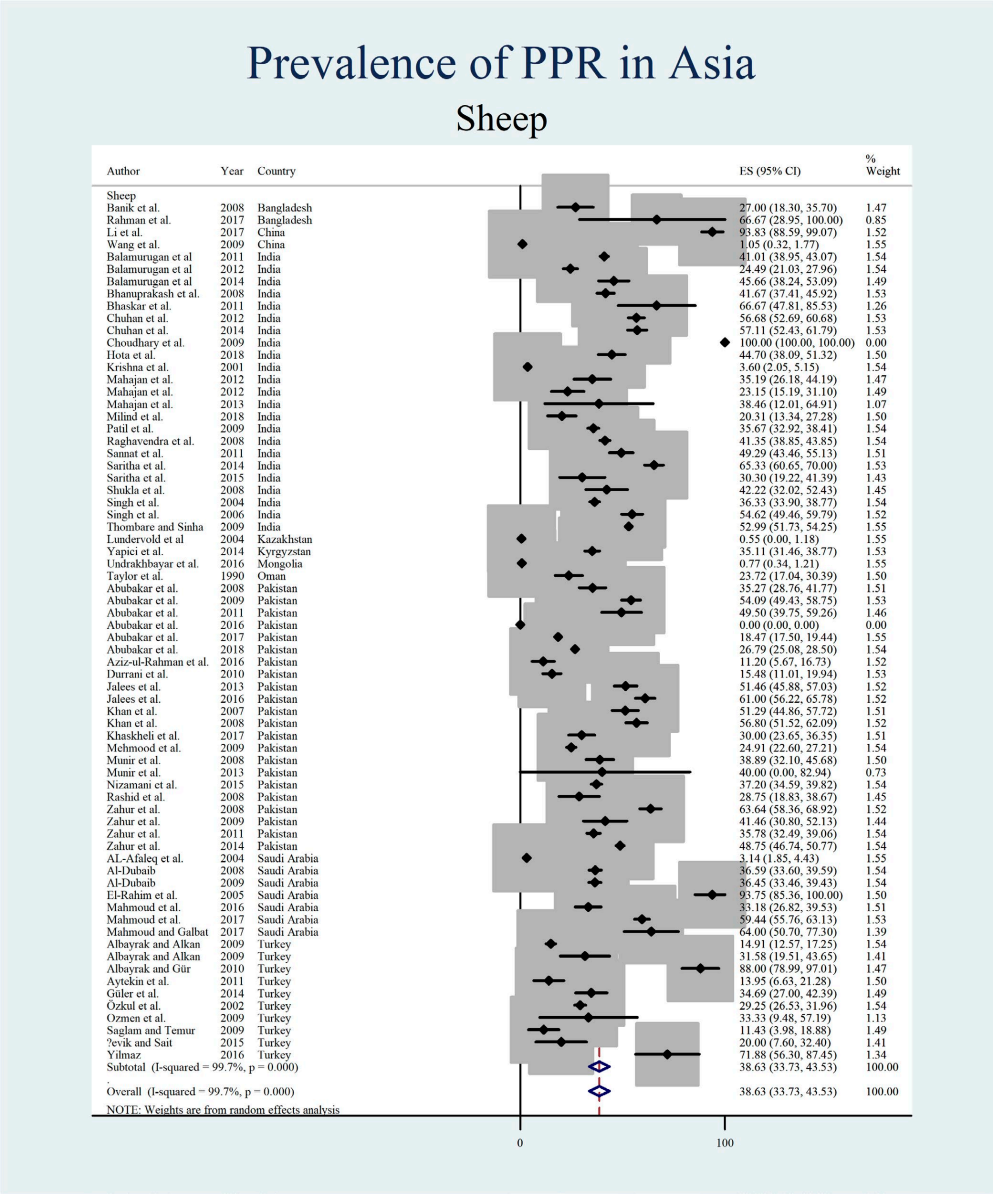
Forest plot of the prevalence estimates of Peste des petits ruminants (PPR) in sheep amongst studies conducted in Asia

**FIGURE 7:**
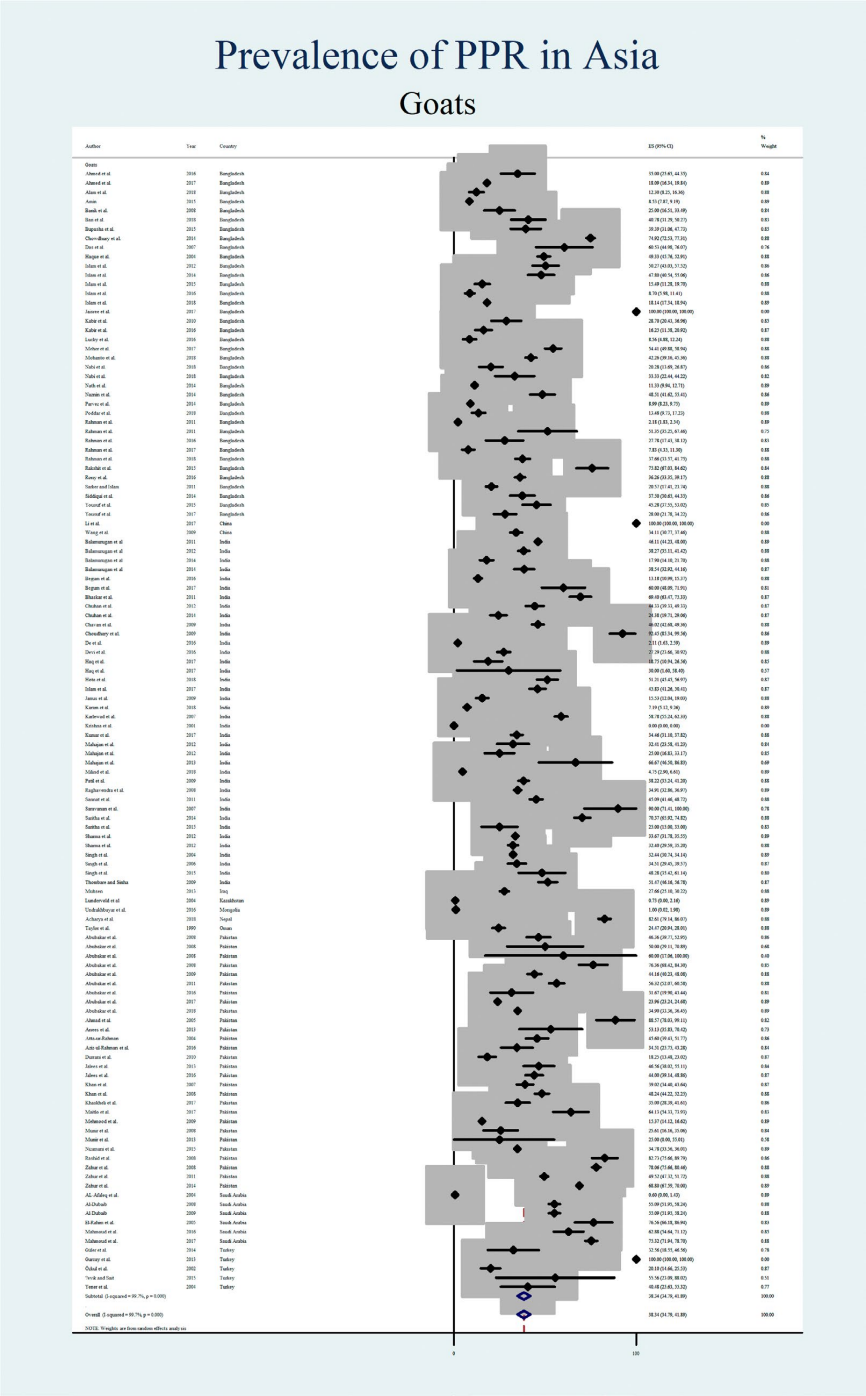
Forest plot of the prevalence estimates of Peste des petits ruminants (PPR) in goats amongst studies conducted in Asia

Figures 4, 5, 6, 7 show the prevalence estimates from individual contributing studies by world region. Country‐wise subgroup analysis showed that the lowest prevalence was reported as 0.59% (95% CI: 0.07%–1.11%, χ2: 0.08, *I* ^2^ = 0.0, *p* < .774) in Kazakhstan and the highest prevalence as 84.15% (95% CI: 70.17%–98.13%; χ2: 0.01, *I* ^2^ = 0.0, *p* < .931) in Benin. Age‐wise subgroup analysis showed that the prevalence estimate in young sheep was 41.49% (95% CI: 29.47%–53.50%; χ2: 1718.98, *I* ^2^ = 99.2, *p* < .0001) and in young goats was 33.44% (95% CI: 27.84%–39.04%; χ2: 4,367.58, *I* ^2^ = 99.3 *p* < .0001), while the prevalence estimate in adult sheep was 48.40% (95% CI: 36.21%–60.60%; χ2: 2,125.88, *I* ^2^ = 99.3, *p* < .0001) and in adult goat was 35.91% (95% CI: 28.47%–43.35%; χ2: 8,235.89, *I* ^2^ = 99.6, *p* < .0001). Sex‐wise subgroup analysis showed that the prevalence estimate in male sheep was 35.09 (95% CI: 25.65%–44.54%; χ2: 305.95, *I* ^2^ = 96.1, *p* < .0001) and male goats was 35.22% (95% CI: 29.19%–41.25%; χ2: 1607.66, *I* ^2^ = 97.8, *p* < .0001), while the prevalence estimate in female sheep was 40.55 (30.11%–51.00%; χ2: 806.63, *I* ^2^ = 98.5, *p* < .0001) and in female goats was 41.18 (34.07%–48.29%; χ2: 4,195.33 i^2^ = 99.2 *p* < .0001).

Pooled prevalences based on age, sex, origin of samples, methods of detection and study duration are shown in Table 3. The overall estimated pooled prevalence of PPR in sheep and goats slaughtered in abattoirs was 42.72% (95% CI: 37.97%–47.47%) while the prevalence in hospital‐based studied animals was 30.15% (95% CI: 26.95%–33.36%). Prevalence estimates were 48.03% (95% CI: 36.32%–59.74%) in studies that used PCR and 32.32% (95% CI: 7.76%–56.87%) in studies that used immunohistochemistry to detect PPR in sheep and goats. Studies conducted for less than or equal to 6 months had higher prevalence, at 41.47% (95% CI: 36.09%–46.85%), than those conducted for more than12 months [38.64% (95% CI: 35.92%–45.07%)] and for between 6 and 12 months [31.39% (95% CI: 28.37%–34.42%)].

### 3.4 Source of heterogeneity

Heterogeneity in the prevalence of PPR in sheep and goats was due to six sources: world region (*p* < .0001), age (*p* < .0001), sex (*p* < .0001), origin of the sample (*p* < .0001), method of detection (*p* < .0001) and study duration (*p* < .0001). Overall, there was substantial heterogeneity (*I* ^2^ > 80%) in most pooled prevalence estimates (Table 3).

**FIGURE 8:**
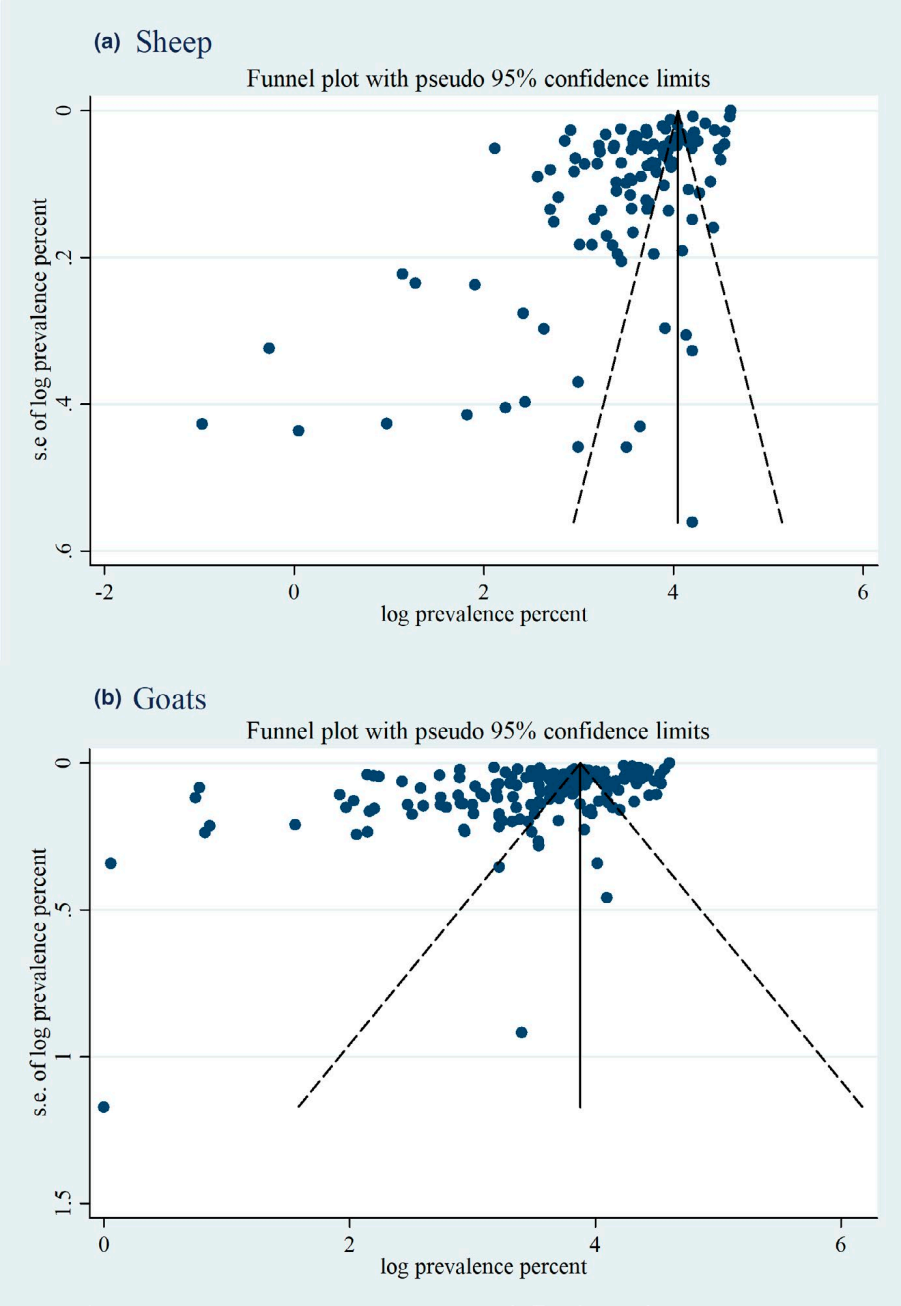
Funnel plot for examination of publication bias of the prevalence estimates of Peste des petits ruminants (PPR) in sheep (a) and goat (b). *Abbreviation*: *SE*, standard error

The extent of publication bias in the included articles was measured separately for sheep and goats. Funnel plots indicated that there was publication bias (Figure 8). Egger's test for publication bias also showed that there was a small study effect. The estimated bias coefficient in sheep was 4.28 (95% CI: 4.18–4.37) with a standard error of 0.049 providing a *p*‐value of <.0001, while the estimated bias coefficient in goats was 4.09 (95% CI: 3.98–4.20) with a standard error of 0.054 and a *p*‐value of <.0001. An assessment checklist for possible bias and scores of individual studies is supplied (Appendix S2, Table S2).

## 4 DISCUSSION

This systemic review and meta‐analysis summarises the prevalence of PPR in sheep and goats at regional level, based on a large population (*n* = 243,864). Articles included were from 34 countries from two continents, which allowed the analysis of reliable prevalence estimates, to increase knowledge of PPR epidemiology and consequently inform PPR control and eradication. This is the first meta‐analysis of the prevalence of PPR in sheep and goats at a regional level to the best of the author's knowledge.

The analysis showed a high prevalence of PPR in sheep and goats in most endemic countries of Africa and Asia, with an estimated pooled prevalence of 39.46% (95% CI: 37.23%–41.69%) across 196 published studies. Although countries of Africa and Asia had the highest reported disease prevalence, it has recently been reported that PPR is emerging in ruminant populations in Europe (Parida et al., 2016). However, because of a lack of individual‐level prevalence data or studies being in species other than sheep and goat, studies from Europe were not included in this meta‐analysis.

Africa had a higher disease prevalence than Asia. Most of the individual studies showed a high prevalence; however prevalence estimates were low in only a few studies (Ameen & Ajayi, 2013; Cêtre‐Sossah et al., 2016; Ezeokoli et al., 1986). Reasons for the high prevalence of PPR in sheep and goats could include transboundary movement of infected animals with inadequate quarantine, the presence of hot and humid climatic conditions that favour disease epidemiology, lack of vaccination or vaccine administration monitoring, lack of awareness about PPR among backyard farmers, and limited funding for disease eradication in developing or underdeveloped countries. Moreover many studies included in this meta‐analysis used serum sample or symptomatic diagnostic approaches to report PPR prevalence; such approaches can quickly reveal the status of a large population (Delil et al., 2012; El‐Rahim et al., 2010; Mahamat et al., 2018; Parvez et al., 2014).

The estimated pooled prevalence indicated that PPR is equally prevalent in both sheep and goats. Prevalence was higher in goats than sheep, but the difference was not statistically significant (*p* = .402). Such findings are consistent with other research that reported an equal prevalence of PPR in sheep and goats (Balamurugan, Saravanan, et al., 2012). Several individual studies reported that PPR is more prevalent in sheep than goats (Abubakar et al., 2009; El‐Yuguda et al., 2010; Enan et al., 2013) or more prevalent in goats than sheep (Delil et al., 2012; Farougou et al., 2013; Fentie et al., 2018). Although there are biological differences between sheep and goats, higher prevalence in one species than another could be due to factors such as sampling process, richness or distribution of animal in a geographical area, management practices and strain of the virus. It is also possible that PPRV preferentially infects goats over sheep or vice‐versa, depending on the endemic situation, and disease severity may also vary between species (Truong et al., 2014).

PPR was higher in adult animals than in young animals based on estimated pooled prevalence. These results agree with the findings of many studies (Abubakar et al., 2009, 2011, 2017; Acharya et al., 2018; Gari et al., 2017), but not with several others (Alam et al., 2018; Bari et al., 2018; Bello et al., 2018). The higher prevalence in adults could be due to factors such as the higher likelihood of older animals being exposed to PPRV because of virus circulation, the foraging behaviour of adult animals and the decay of maternally derived antibody in older animals. It has been reported that PPRV is highly immunogenic, and animals remain seropositive for a long period, particularly in an endemic area (Acharya et al., 2018; Balamurugan, Sen, et al., 2012). In contrast, higher prevalence in young animals could be due to malnutrition, less developed immune system and poor husbandry practices (Bari et al., 2018).

The estimated pooled prevalence of PPR was higher in female animals than in male animals. This could be due to breeding females being used for flock reproduction maintenance for a more extended period than males, while males are sold for meat at an early age (1–2 years) except those used for breeding purpose. Other factors could be a higher density of females than males in flocks, or physiological differences between females and males (e.g. females face some degree of stress as a result of production and reproduction). The findings of this study are in agreement with previous findings (El‐Yuguda et al., 2013; Farougou et al., 2013; Mahamat et al., 2018). Other studies found a higher prevalence in males, possibly due to a higher proportion of male animals in a flock particularly when the age of the studied animals was under two years (Rony et al., 2017).

The variation in the prevalence data in this report also depended on the source of the sample, the method of detection and the length of the study. Prevalence was higher in animals originating from abattoirs and farms, and in animals kept in mixed flocks. This may be due to there being a higher chance of having sick animals in abattoirs (because many farmers sell animals for slaughter during outbreaks) or to animals in abattoir coming from different regions and different farms; alternatively, it may be due to the method of detection in case of mixed flocks and farm‐based studies, given that most of the studies were conducted using serology (Goossens et al., 1998; Rashid et al., 2008). Cross‐transmission of PPRV between sheep and goats, or persistence of the virus in mixed flocks could be another contributing factor (Gari et al., 2017). Based on the method of detection, the prevalence was higher where the PCR‐based method was used; this is due to PCR being more sensitive than other methods. Studies conducted for less than six months showed a higher prevalence, and this could be due to a difference in study design.

Egger's test results using a linear regression approach and funnel plots asymmetry revealed strong evidence of publication bias. However, the source of funnel plot asymmetry could additionally be due to true heterogeneity, location, data irregularity and artefacts or even to chance (Egger et al., 1997).

### 4.1 Limitations

This study has several limitations. First, no reports on PPR prevalence in sheep and goats were found in continents other than Africa and Asia within the search range. Thus, it was not possible to obtain information on the prevalence from these regions. Second, age is an important factor to PPR, but there were not enough articles on age available for inclusion to carry out a multiple subgroup analysis; hence, the data are presented as young and adult. Third, the review excluded non‐English articles, unpublished articles, retro perspective articles, method validation articles, results of experimental trials and case reports. Fourth, the genotype/sequencing data used to identify countries that are endemic for PPR or have PPR outbreaks (e.g. Bulgaria) have shown that PPRV is prevalent in many countries of the world, but it was not possible to include them in this study because of the lack of prevalence data,. Finally, heterogeneity in models was significant, suggesting that other factors that were not considered might have had substantial effects.

## 5 CONCLUSIONS

The Africa‐Asia pooled prevalence of PPR in sheep and goats estimated through this meta‐analysis was high, and it varied between geographical regions and countries. The disease was found to be more prevalent in Africa than in Asia, and more prevalent in adult animals; thus, vaccination of young animals may prevent the disease. The findings suggest that screening tests for PPR, and effective preventive and eradication measures, should be routinely carried out in sheep and goat flocks in regions with a high disease prevalence, to control the outbreak and improve animal productivity. Further, there are regions where the virus is circulating, but no reports on prevalence estimates are available; therefore, epidemiological surveillance is needed for estimating disease status and eliminating the disease. Additionally, factors that contribute to the prevalence estimate heterogeneity should be handled appropriately in any survey to accurately estimate the true extent of PPR.

## CONFLICT OF INTEREST

The author declares that he has no competing interests.

## AUTHOR CONTRIBUTION

**Md Ahaduzzaman:** Conceptualization; Data curation; Formal analysis; Investigation; Methodology; Software; Visualization; Writing‐original draft; Writing‐review & editing.

## Supporting information

Appendix S1

Appendix S2

Appendix S3

Appendix S4

## References

[vms3300-bib-0001] Abdalla, A. , Majok, A. , El Malik, K. , & Ali, A. (2012). Sero‐prevalence of Peste des petits ruminants virus (PPRV) in small ruminants in Blue Nile, Gadaref and North Kordofan States of Sudan. Journal of Public Health and Epidemiology, 4(3), 59–64.

[vms3300-bib-0002] Abraham, G. , Sintayehu, A. , Libeau, G. , Albina, E. , Roger, F. , & Laekemariam, Y. (2005). Antibody seroprevalences against Peste des petits ruminants (PPR) virus in camels, cattle, goats and sheep in Ethiopia. Preventive Veterinary Medicine, 70(1–2), 51–57. 15967242 10.1016/j.prevetmed.2005.02.011

[vms3300-bib-0003] Abubakar, M. , Ali, Q. , & Khan, H. A. (2008). Prevalence and mortality rate of peste des petitis ruminant (PPR): Possible association with abortion in goat. Tropical Animal Health and Production, 40(5), 317–321. 18509938 10.1007/s11250-007-9105-2

[vms3300-bib-0004] Abubakar, M. , Jamal, S. M. , Arshed, M. J. , Hussain, M. , & Ali, Q. (2009). Peste des petits ruminants virus (PPRV) infection; its association with species, seasonal variations and geography. Tropical Animal Health and Production, 41(7), 1197–1202. 19130284 10.1007/s11250-008-9300-9

[vms3300-bib-0005] Abubakar, M. , Jamal, S. M. , Hussain, M. , & Ali, Q. (2008). Incidence of Peste des petits ruminants (PPR) virus in sheep and goat as detected by immuno‐capture ELISA (Ic ELISA). Small Ruminant Research, 75(2–3), 256–259.

[vms3300-bib-0006] Abubakar, M. , Jamal, S. , Khan, M. A. , & Ali, Q. (2008). Peste des petits ruminants outbreak in small ruminants of Northern areas of Pakistan. Research in Veterinary Science, 1(1), 56–61.

[vms3300-bib-0007] Abubakar, M. , Javed Arshed, M. , Hussain, M. , & Ali, Q. (2011). Evidence of Peste des petits ruminants in serology of sheep and goats from Sindh, Pakistan. Transboundary and Emerging Diseases, 58(2), 152–166. 21167015 10.1111/j.1865-1682.2010.01193.x

[vms3300-bib-0008] Abubakar, M. , Manzoor, S. , Wensman, J. J. , Torsson, E. , Qurban, A. , & Munir, M. (2016). Molecular and epidemiological features of Peste des petits ruminants outbreak during endemic situation. Hosts and Viruses, 3(4), 123–129.

[vms3300-bib-0009] Abubakar, M. , Rasool, M. H. , Manzoor, S. , Saqalein, M. , Rizwan, M. , Munir, M. , … Wensman, J. J. (2016). Evaluation of risk factors for Peste des petits ruminants virus in sheep and goats at the Wildlife‐Livestock Interface in Punjab Province, Pakistan. BioMed Research International, 2016, 1–6. 10.1155/2016/7826245 PMC4884585 27294134

[vms3300-bib-0010] Abubakar, M. , Zahur, A. B. , Afzal, M. , Ali, Q. , & Gonzales, J. (2017). Peste des petits ruminants (PPR) in Pakistan: Analysis of a national level serological data. Small Ruminant Research, 155, 57–65.

[vms3300-bib-0011] Abubakar, M. , Zahur, A. B. , Naeem, K. , Khan, M. A. , & Qureshi, S. (2018). Field and molecular epidemiology of Peste des petits ruminants in Pakistan. Pakistan Journal of Zoology, 50(2), 559–566.

[vms3300-bib-0012] Acharya, N. , Poudel, S. P. , & Acharya, K. P. (2018). Cross‐sectional sero‐prevalence study of Peste des petits ruminants (PPR) in goats of Syangja and Kaski districts of Nepal. Virus Disease, 29(2), 173–179. 29911150 10.1007/s13337-018-0449-1 PMC6003051

[vms3300-bib-0013] Adel, A.‐A. , Abu‐Elzein, E. , Al‐Naeem, A.‐M. , & Amin, M. (2004). Serosurveillance for Peste des petits ruminants (PPR) and rinderpest antibodies in naturally exposed Saudi sheep and goats. Veterinarski Arhiv, 74(6), 459–465.

[vms3300-bib-0014] Adombi, C. , Waqas, A. , Dundon, W. , Li, S. , Daojin, Y. , & Kakpo, L. (2017). Peste des petits ruminants in Benin: Persistence of a single virus genotype in the country for over 42 years. Transboundary and Emerging Diseases, 64(4), 1037–1044. 26801518 10.1111/tbed.12471

[vms3300-bib-0015] Afera, B. , Hussien, D. , & Amsalu, K. (2014). Seroprevalence of Peste des petits ruminants in goats of southern parts of Tigray region. Global Veterinaria, 12, 512–516.

[vms3300-bib-0016] Ahaduzzaman, M. (2019). The global and regional prevalence of oestrosis in sheep and goats: A systematic review of articles and meta‐analysis. Parasites & Vectors, 12(1), 346. 31300017 10.1186/s13071-019-3597-2 PMC6625052

[vms3300-bib-0017] Ahmad, K. , Jamal, S. , Ali, Q. , & Hussain, M. (2005). An outbreak of Peste des petits ruminants (PPR) in a goat flock in Okara, Pakistan. Pakistan Veterinary Journal, 209(5), 146–148.

[vms3300-bib-0018] Ahmed, M. S. , Riadul, H. , Hossain, M. A. , Uddin, F. , Rashid, H. , & Talha, M. H. (2017). Clinical prevalence and influencing factors analysis for the occurrence of Peste des petits ruminants (PPR) disease of goat at Sylhet region, Bangladesh. Veterinary Clinical Science, 5(1), 1–5.

[vms3300-bib-0019] Ahmed, S. , Rahman, F. , Alam, M. , Paul, S. , & Uddin, M. (2016). Risk factors for Peste des petits ruminants: A hospital basedcase‐control study. International Journal of Natural Sciences, 6(1), 17–20.

[vms3300-bib-0020] Alam, M. B. , Mahmud, T. , Khan, S. A. , Islam, A. , Hai, M. A. , & Hassan, M. M. (2018). Occurrence of diseases and disease conditions in cattle and goats at the Upazilla Veterinary Hospital, Debidwar, Comilla. Journal of Advanced Veterinary and Animal Research, 5(2), 117–122.

[vms3300-bib-0021] Albayrak, H. , & Alkan, F. (2009). PPR virus infection on sheep in blacksea region of Turkey: Epidemiology and diagnosis by RT‐PCR and virus isolation. Veterinary Research Communications, 33(3), 241–249. 10.1007/s11259-008-9172-5 18787968

[vms3300-bib-0022] Albayrak, H. , & Gür, S. (2010). A serologic investigation for Peste des petits ruminants infection in sheep, cattle and camels (Camelus dromedarius) in Aydın province, West Anatolia. Tropical Animal Health and Production, 42(2), 151–153. 19554466 10.1007/s11250-009-9400-1

[vms3300-bib-0023] Al‐Dubaib, M. (2008). Prevalence of Peste despetitis ruminants infection in sheep and goat farms at the Central Region of Saudi Arabia. Research Journal of Veterinary Sciences, 1(1), 67–70.

[vms3300-bib-0024] Al‐Dubaib, M. (2009). Peste des petitis ruminants morbillivirus infection in lambs and young goats at Qassim region, Saudi Arabia. Tropical Animal Health and Production, 41(2), 217–220. 18504645 10.1007/s11250-008-9178-6

[vms3300-bib-0025] Ali, Y. , Intisar, K. , & Khalafalla, A. (2014). Outbreaks of Peste des petits ruminants in two different localities in Sudan. Journal of Veterinary Medicine and Animal Health, 6(6), 174–177.

[vms3300-bib-0026] Al‐Majali, A. M. , Hussain, N. O. , Amarin, N. M. , & Majok, A. A. (2008). Seroprevalence of, and risk factors for, Peste des petits ruminants in sheep and goats in Northern Jordan. Preventive Veterinary Medicine, 85(1–2), 1–8. 18291541 10.1016/j.prevetmed.2008.01.002

[vms3300-bib-0027] Almeshay, M. D. , Gusbi, A. , Eldaghayes, I. , Mansouri, R. , Bengoumi, M. , & Dayhum, A. S. (2017). Peste des petits ruminants in Tripoli Region, Lybia. Veterinaria Italiana, 53(3), 235–242. 29152705 10.12834/vetit.964.5025.3

[vms3300-bib-0028] Ameen, S. , & Ajayi, J. (2013). Studies on influence of seasonality on clinical conditions of small ruminants in Ogbomoso areas of Oyo State. International Journal of Applied Agriculture and Apiculture Research, 9(1–2), 18–27.

[vms3300-bib-0029] Amin, M. R. (2015). Prevalence of common parasitic and infectious diseases of goat at Babugonj upazilla, Barisal, Bangladesh. Asian Journal of Medical and Biological Research, 1(3), 449–456.

[vms3300-bib-0030] Anderson, J. , & McKay, J. (1994). The detection of antibodies against Peste des petits ruminants virus in cattle, sheep and goats and the possible implications to rinderpest control programmes. Epidemiology & Infection, 112(1), 225–231. 8119361 10.1017/s0950268800057599 PMC2271469

[vms3300-bib-0031] Anees, M. , Shabbir, M. Z. , Muhammad, K. , Nazir, J. , Shabbir, M. A. B. , & Wensman, J. J. (2013). Genetic analysis of Peste des petits ruminants virus from Pakistan. BMC Veterinary Research, 9(1), 60. 23537146 10.1186/1746-6148-9-60 PMC3639103

[vms3300-bib-0032] Atta‐ur‐Rahman, M. A. , Rahman, S. , Akhtar, M. , & Ullah, S. (2004). Peste des petits ruminants antigen in mesenteric lymph nodes of goats slaughtered at DI Khan. Pakistan Veterinary Journal, 24(3), 159–160.

[vms3300-bib-0033] Ayim‐Akonor, M. , Obese, F. , Arthur, C. , Owusu‐Ntumy, D. , & Otsyina, H. (2014). Molecular detection and differentiation of Peste des Petits Ruminant virus and Rinderpest virus in sheep and goats with PPR‐like symptoms in Dangme West District of Ghana. International Journal of Current Research and Academic Review, 2(6), 197–205.

[vms3300-bib-0034] Aytekin, İ. , Mamak, N. , Ulucan, A. , & Kalınbacak, A. (2011). Clinical, haematological, biochemical and pathological findings in lambs with Peste des petits ruminants.

[vms3300-bib-0035] Baazizi, R. , Ait‐Oudhia, K. , Parida, S. , Mahapatra, M. , & Khelef, D. (2015). Peste of small ruminants in algeria: Virus circulation by serosurvey preliminary results. Egyptian Journal of Sheep and Goat Sciences, 65(2363), 1–2.

[vms3300-bib-0036] Baazizi, R. , Khelef, D. , & Hussain, T. (2017). Peste des petits ruminants in Algeria: Viral circulation of PPRV between 2012 and 2015. Journal of Animal and Plant Sciences, 27(5), 1522–1527.

[vms3300-bib-0037] Balamurugan, V. , Das, S. , Raju, D. , Chakravarty, I. , Nagalingam, M. , & Hemadri, D. (2014). Prevalence of Peste des petits ruminants in goats in North‐East India. Virus Disease, 25(4), 488–492. 25674627 10.1007/s13337-014-0237-5 PMC4262313

[vms3300-bib-0038] Balamurugan, V. , Hemadri, D. , Gajendragad, M. , Singh, R. , & Rahman, H. (2014). Diagnosis and control of Peste des petits ruminants: A comprehensive review. Virusdisease, 25(1), 39–56. 24426309 10.1007/s13337-013-0188-2 PMC3889233

[vms3300-bib-0039] Balamurugan, V. , Krishnamoorthy, P. , Raju, D. , Rajak, K. , Bhanuprakash, V. , & Pandey, A. (2014). Prevalence of Peste‐des‐petits‐ruminant virus antibodies in cattle, buffaloes, sheep and goats in India. Virus Disease, 25(1), 85–90. 24426314 10.1007/s13337-013-0177-5 PMC3889243

[vms3300-bib-0040] Balamurugan, V. , Saravanan, P. , Sen, A. , Rajak, K. , Bhanuprakash, V. , & Krishnamoorthy, P. (2011). Sero‐epidemiological study of Peste des petits ruminants in sheep and goats in India between 2003 and 2009. Revue Scientifique Et Technique‐OIE, 30(3), 889–896. 10.20506/rst.30.3.2087 22435199

[vms3300-bib-0041] Balamurugan, V. , Saravanan, P. , Sen, A. , Rajak, K. K. , Venkatesan, G. , & Krishnamoorthy, P. (2012). Prevalence of Peste des petits ruminants among sheep and goats in India. Journal of Veterinary Science, 13(3), 279–285. 23000584 10.4142/jvs.2012.13.3.279 PMC3467403

[vms3300-bib-0042] Balamurugan, V. , Sen, A. , Venkatesan, G. , Rajak, K. K. , Bhanuprakash, V. , & Singh, R. K. (2012). Study on passive immunity: Time of vaccination in kids born to goats vaccinated against Peste des petits ruminants. Virologica Sinica, 27(4), 228–233. 10.1007/s12250-012-3249-6 22899430 PMC8218118

[vms3300-bib-0043] Banik, S. , Podder, S. , Samad, M. , & Islam, M. (2008). Sero‐surveillance and immunization in sheep and goats against Peste des petits ruminants in Bangladesh. Bangladesh Journal of Veterinary Medicine, 6(2), 185–190.

[vms3300-bib-0044] Banyard, A. C. , Parida, S. , Batten, C. , Oura, C. , Kwiatek, O. , & Libeau, G. (2010). Global distribution of Peste des petits ruminants virus and prospects for improved diagnosis and control. Journal of General Virology, 91(12), 2885–2897. 10.1099/vir.0.025841-0 20844089

[vms3300-bib-0045] Bari, M. S. , Rana, E. A. , Ahaduzzaman, M. , Al Masud, A. , Das, T. , & Hasan, T. (2018). Hemato‐biochemical parameters of Pesti‐des Petits Ruminants (PPR) affected goats in Chittagong, Bangladesh. Journal of Advanced Veterinary and Animal Research, 5(2), 211–217.

[vms3300-bib-0046] Begum, S. , Mahato, G. , Muthuchelvan, D. , Chaudhary, D. , Hussain, M. , & Nashiruddullah, N. (2017). Molecular identification of Peste‐des‐petits‐ruminant virus from goats of Assam state of North‐East India. Journal of Entomology and Zoology Studies, 5(2), 1366–1368.

[vms3300-bib-0047] Begum, S. S. , Mahato, G. , Sharma, P. , Sharma, K. , Hussain, M. , & Das, B. C. (2016). Seroprevalence of Peste des petits ruminants in goats in Assam, India. Asian Journal of Animal and Veterinary Advances, 11(3), 210–222.

[vms3300-bib-0048] Bello, A. , Lawal, J. , Dauda, J. , Wakil, Y. , Lekko, Y. , & Mshellia, E. (2016). Research for Peste des petits ruminants (PPR) virus antibodies in goats, sheep and gazelle from Bauchi and Gombe States, north eastern Nigeria. Direct Research Journal of Agriculture and Food Science, 4(8), 193–198.

[vms3300-bib-0049] Bello, M. , Kazeem, H. , Oladele, S. , Fatihu, M. , Tambuwal, F. , & Jibril, A. (2018). Seroprevalence of Peste des petits ruminants among unvaccinated small ruminants in Sokoto State, northwestern Nigeria. Comparative Clinical Pathology, 27(5), 1141–1146.

[vms3300-bib-0050] Bhanuprakash, V. , Saravanan, P. , Hosamani, M. , Balamurugan, V. , Mondal, B. , & Singh, R. K. (2008). Status of sheep sera to bluetongue, Peste des petits ruminants and sheep pox in a few northern states of India. Veterinaria Italiana, 44(3), 527–536. 20405449

[vms3300-bib-0051] Bhaskar, S. , Deshmukh, V. , Chopade, N. , Rautmare, S. , & Aziz, A. (2011). Peste des petits ruminants (PPR) outbreak in sheep and goats in Maharashtra: Laboratory confirmation by s‐ELISA (Mukteshwar) and vero cell culture. Animal Science Reporter, 5(2), 64–68.

[vms3300-bib-0052] Birindwa, B. A. , George, G. C. , Ntagereka, B. P. , Christopher, O. , & Lilly, B. C. (2017). Mixed infection of peste‐des‐petits ruminants and Capripox in goats in South Kivu, Democratic Republic of Congo. Journal of Advanced Veterinary and Animal Research, 4(4), 348–355.

[vms3300-bib-0053] Bupasha, Z. , Hossain, F. , Sarker, M. , Ahaduzzaman, M. , & Biswas, P. (2015). Variability in prevalence and therapeutic effectiveness in PPR affected goats of Thakurgoan, Bangladesh. Annals of Veterinary and Animal Science, 2, 15–19.

[vms3300-bib-0054] Cêtre‐Sossah, C. , Kwiatek, O. , Faharoudine, A. , Soulé, M. , Moutroifi, Y. , & Vrel, M.‐A. (2016). Impact and epidemiological investigations into the incursion and spread of Peste des petits ruminants in the Comoros Archipelago: An increased threat to surrounding Islands. Transboundary and Emerging Diseases, 63(4), 452–459. 25430822 10.1111/tbed.12296

[vms3300-bib-0055] Chauhan, H. , Dadawala, A. , Chandel, B. , Kalyani, I. , Patel, S. S. , & Kher, H. (2012). Seroprevalence of Peste des petits ruminants in small ruminants under different managemental conditions. Indian Journal of Field Veterinarians, 7(3), 37–39.

[vms3300-bib-0056] Chauhan, H. C. , Kher, H. , Rajak, K. K. , Sen, A. , Dadawala, A. I. , & Chandel, B. S. (2014). Epidemiology and diagnosis of Peste des petits ruminants in sheep and goats by Serological, molecular and isolation methods in Gujarat, India. Advanced in Animal and Veterinary Sciences, 2(4), 192–198.

[vms3300-bib-0057] Chavan, V. , Digraskar, S. , Dhonde, S. , & Bedarkar, S. (2009). Seromonitoring of Peste des petits ruminants (PPR) in goats (Capra hircus) of Parbhani region of Maharashtra. Veterinary World, 2, 8.

[vms3300-bib-0058] Choudhary, P. , Jhala, M. , & Kanani, A. (2009). Incidence of PPR virus in Gujarat by s‐ELISA and molecular detection by F, N and H gene based RT‐PCR. Royal Veterinary Journal of India, 5(1/2), 1–4.

[vms3300-bib-0059] Chowdhury, E. H. , Bhuiyan, A. R. , Rahman, M. M. , Siddique, M. S. A. , & Islam, M. R. (2014). Natural Peste des petits ruminants virus infection in Black Bengal goats: Virological, pathological and immunohistochemical investigation. BMC Veterinary Research, 10(1), 263. 25394771 10.1186/s12917-014-0263-y PMC4233235

[vms3300-bib-0060] Das, K. K. , Shil, N. K. , & Islam, M. R. (2007). Sero‐epidemiological investigation on Peste des petits ruminants in black Bengal goats. Bangladesh Journal of Microbiology, 24(2), 143–145.

[vms3300-bib-0061] De, A. , Debnath, B. , Dutta, T. , Shil, S. , Bhadouriya, S. , & Chaudhary, D. (2016). Sero‐epidemiology of Peste‐des‐petits‐ruminants in goats of Tripura state of North‐East India. Advances in Animal and Veterinary Sciences, 4(5), 215–217.

[vms3300-bib-0062] Delil, F. , Asfaw, Y. , & Gebreegziabher, B. (2012). Prevalence of antibodies to Peste des petits ruminants virus before and during outbreaks of the disease in Awash Fentale district, Afar, Ethiopia. Tropical Animal Health and Production, 44(7), 1329–1330. 22359089 10.1007/s11250-012-0110-8

[vms3300-bib-0063] Devi, M. , Das, S. , Sharma, K. , & Dutta, R. (2016). Seroprevelance and molecular detection of Peste des petits ruminants in goats of Assam. Virus Disease, 27(1), 91–97. 26925449 10.1007/s13337-015-0291-7 PMC4758308

[vms3300-bib-0064] Diallo, A. , Minet, C. , Le Goff, C. , Berhe, G. , Albina, E. , & Libeau, G. (2007). The threat of Peste des petits ruminants: Progress in vaccine development for disease control. Vaccine, 25(30), 5591–5597. 17399862 10.1016/j.vaccine.2007.02.013

[vms3300-bib-0065] Dilli, H. , Geidam, Y. , & Egwu, G. (2011). Peste de petits ruminants in Nigeria: A review. Nigerian Veterinary Journal, 32, 2.

[vms3300-bib-0066] Durrani, A. Z. , Kamal, N. , Mehmood, N. , & Shakoori, A. R. (2010). Prevalence of Peste des petits ruminants (KATA) in sheep and goats of Punjab. Pakistan Journal of Zoology, 42, 3.

[vms3300-bib-0067] Egger, M. , Smith, G. D. , Schneider, M. , & Minder, C. (1997). Bias in meta‐analysis detected by a simple, graphical test. BMJ, 315(7109), 629–634. 9310563 10.1136/bmj.315.7109.629 PMC2127453

[vms3300-bib-0068] Elhaig, M. M. , Selim, A. , Mandour, A. S. , Schulz, C. , & Hoffmann, B. (2018). Prevalence and molecular characterization of Peste des petits ruminants virus from Ismailia and Suez, Northeastern Egypt, 2014–2016. Small Ruminant Research, 169, 94–98. 10.1016/j.smallrumres.2018.07.001

[vms3300-bib-0069] El‐Rahim, I. , Baky, M. , Habashi, A. , Mahmoud, M. , & Al‐Mujalii, D. (2005). Peste des petits ruminants among sheep and goats in Saudi Arabia in 2004. Assuit Veterinary Medicine Journal, 51, 100–111.

[vms3300-bib-0070] El‐Rahim, I. , Sharawi, S. , Barakat, M. , & El‐Nahas, E. (2010). An outbreak of Peste des petits ruminants in migratory flocks of sheep and goats in Egypt in 2006. Scientific and Technical Review of the Office International Des Epizooties, 29(3), 655–662. 10.20506/rst.29.3.2004 21309463

[vms3300-bib-0071] El‐Yuguda, A. , Abubakar, M. , Nabi, A. , Andrew, A. , & Baba, S. (2009). Outbreak of Peste des petits ruminant in an unvaccinated Sahel goat farm in Maiduguri, Nigeria. African Journal of Biomedical Research, 12(1), 83–87.

[vms3300-bib-0072] El‐Yuguda, A. , Chabiri, L. , Adamu, F. , & Baba, S. (2010). Peste des petits ruminants virus (PPRV) infection among small ruminants slaughtered at the central abattoir, Maiduguri, Nigeria. Sahel Journal of Veterinary Science, 8(2), 93–96.

[vms3300-bib-0073] El‐Yuguda, A.‐D. , Saheed Baba, S. , Ganiyu Ambali, A. , & Egwu, G. O. (2013). Seroprevalence of Peste des petits ruminants among domestic small and large ruminants in the semi‐arid region of North‐eastern Nigeria. Veterinary World, 6(10), 807–811. 10.14202/vetworld.2013.807-811

[vms3300-bib-0074] Enan, K. , Intisar, K. , Haj, M. , Hussien, M. , Taha, K. , & Elfahal, A. (2013). Seroprevalence of two important viral diseases in small ruminants in Marawi Province Northern State, Sudan. International Journal of Livestock Production, 4(2), 18–21. 10.5897/IJLP11.048

[vms3300-bib-0075] Ezeokoli, C. , Umoh, J. , Chineme, C. , Isitor, G. , & Gyang, E. (1986). Clinical and epidemiological features of Peste des petits ruminants in Sokoto Red goats. Revue D’elevage Et De Medecine Veterinaire Des Pays Tropicaux, 39(3–4), 269–273. 3659476

[vms3300-bib-0076] Faris, D. , Yilkal, A. , Berhe, G. , & Kelay, B. (2012). Seroprevalence and sero‐conversion after vaccination against Peste des petits ruminants in sheep and goats from Awash Fentale district Afar, Ethiopia. Preventive Veterinary Medicine, 103(2–3), 157–162. 10.1016/j.prevetmed.2011.10.007 22088269

[vms3300-bib-0077] Farougou, S. , Gagara, M. , & Mensah, G. A. (2013). Prevalence of Peste des petits ruminants in the arid zone in the Republic of Niger. Onderstepoort Journal of Veterinary Research, 80(1), 1–6. 10.4102/ojvr.v80i1.544 24396910

[vms3300-bib-0078] Fentie, T. , Teshome, Y. , Ayele, B. , Molla, W. , Fenta, N. , & Nigatu, S. (2018). Sero‐epidemiological study of Peste des petits ruminants in small ruminants in Amahara region, Ethiopia. Comparative Clinical Pathology, 27(4), 1029–1036. 10.1007/s00580-018-2697-2

[vms3300-bib-0079] Fournié, G. , Waret‐Szkuta, A. , Camacho, A. , Yigezu, L. M. , Pfeiffer, D. U. , & Roger, F. (2018). A dynamic model of transmission and elimination of Peste des petits ruminants in Ethiopia. Proceedings of the National Academy of Sciences, 115(33), 8454–8459. 10.1073/pnas.1711646115 PMC6099864 30054316

[vms3300-bib-0080] Gari, G. , Mekonnen, G. , Sibhat, D. , Abebe, A. , Sahle, M. , & Abie, G. (2015). Participatory disease surveillance (PDS) of sheep and goats deseases in selected districts of Afar Regional State: Particular focus on Pestes des petit ruminants (PPR) and sheep and goat pox disease (SGP). Ethiopian Veterinary Journal, 19(1), 83–105. 10.4314/evj.v19i1.8

[vms3300-bib-0081] Gari, G. , Serda, B. , Negesa, D. , Lemma, F. , & Asgedom, H. (2017). Serological investigation of Peste des petits ruminants in East Shewa and Arsi Zones, Oromia Region, Ethiopia. Veterinary Medicine International. 2017, 1–5. 10.1155/2017/9769071 PMC5745772 29387503

[vms3300-bib-0082] Gibbs, P. J. , Taylor, W. P. , Lawman, M. J. , & Bryant, J. (1979). Classification of Peste des petits ruminants virus as the fourth member of the genus Morbillivirus. Intervirology, 11(5), 268–274. 457363 10.1159/000149044

[vms3300-bib-0083] Goossens, B. , Osaer, S. , Kora, S. , Chandler, K. , Petrie, L. , & Thevasagayam, J. (1998). Abattoir survey of sheep and goats in The Gambia. Veterinary Record, 142(11), 277–281. 10.1136/vr.142.11.277 9569483

[vms3300-bib-0084] Güler, L. , Şevik, M. , & Hasöksüz, M. (2014). Phylogenetic analysis of Peste des petits ruminants virus from outbreaks in Turkey during 2008–2012. Turkish Journal of Biology, 38(5), 671–678. 10.3906/biy-1401-28

[vms3300-bib-0085] Gurcay, M. , Kizil, O. , & Baydar, E. (2013). Peste des petits ruminants (PPR) virus infections in goats in the Eastern anatolia of Turkey. Kafkas Universitesi Veteriner Fakultesi Dergisi, 19, 93–98.

[vms3300-bib-0086] Haq, A. A. , Santhamani, R. , Chakravarti, S. , Yadav, A. K. , Rajak, K. K. , & Upmanyu, V. (2017). Investigation on Peste des Petits ruminants outbreak in goats of Bareilly district of Uttar Pradesh, India. Journal of Immunology and Immunopathology, 19(1), 47–54. 10.5958/0973-9149.2017.00007.7

[vms3300-bib-0087] Haque, M. , Habib, S. , Islam, M. , Khan, K. , & Hannan, A. (2004). Sero‐monitoring of Peste Des petits ruminants (PPR) antibodies in small and large ruminants in Bangladesh. Journal of Animal and Veterinary Advances, 3(7), 453–458.

[vms3300-bib-0088] Haroun, M. , Hajer, I. , Mukhtar, M. , & Ali, B. (2002). Detection of antibodies against Peste des petits ruminants virus in sera of cattle, camels, sheep and goats in Sudan. Veterinary Research Communications, 26(7), 537–541. 12416868 10.1023/a:1020239515020

[vms3300-bib-0089] Higgins, J. P. , & Thompson, S. G. (2002). Quantifying heterogeneity in a meta‐analysis. Statistics in Medicine, 21(11), 1539–1558. 12111919 10.1002/sim.1186

[vms3300-bib-0090] Hota, A. , Biswal, S. , Sahoo, N. , Rout, M. , Chaudhary, D. , & Pandey, A. (2018). Seroprevalence of PPR among sheep and goats of different agroclimatic zones of Odisha. International Journal of Livestock Research. 8, 296–302.

[vms3300-bib-0091] Hoy, D. , Brooks, P. , Woolf, A. , Blyth, F. , March, L. , & Bain, C. (2012). Assessing risk of bias in prevalence studies: Modification of an existing tool and evidence of interrater agreement. Journal of Clinical Epidemiology, 65(9), 934–939. 22742910 10.1016/j.jclinepi.2011.11.014

[vms3300-bib-0092] Intisar, K. , Ali, Y. H. , Haj, M. , Sahar, M. , Shaza, M. , & Baraa, A. (2017). Peste des petits ruminants infection in domestic ruminants in Sudan. Tropical Animal Health and Production, 49(4), 747–754. 10.1007/s11250-017-1254-3 28321790

[vms3300-bib-0093] Ishag, O. , Intisar, K. , & Ali, Y. (2014). Detection of antibodies to Peste des petits ruminants virus using passive haemagglutination test and cELISA in the White Nile state‐Sudan, comparative study. African Journal of Microbiology Research, 8(38), 3475–3481. 10.5897/AJMR2014.7070

[vms3300-bib-0094] Ishag, O. M. , Saeed, I. K. , & Ali, Y. H. (2015). Peste des petits ruminants outbreaks in White Nile State, Sudan. Onderstepoort Journal of Veterinary Research, 82(1), 1–4. 10.4102/ojvr.v82i1.897 PMC6238699 26304168

[vms3300-bib-0095] Islam, K. , Ahad, A. , Mahmood, A. , Rahman, M. M. , Islam, M. Z. , & Bin, M. H. (2014). Prevalence and clinico‐pathological features of Peste des petits ruminants in different breeds of goats and their response to antimicrobials. Journal of Infection and Molecular Biology, 2(3), 43–48. 10.14737/jimb.2307-5465/2.3.43.48

[vms3300-bib-0096] Islam, M. , CPathak, D. , Das, S. , Rahman, T. , Sarma, S. , Hussain, J. , … Gogoi, S. B . (2017). Seroprevalence of Peste des petits ruminants in goats of Assam, India. Journal of Entomology and Zoology Studies, 5(5), 899–901.

[vms3300-bib-0097] Islam, M. M. , Hasan, M. A. , Yousuf, M. A. , Islam, U. K. , Shawan, M. M. A. K. , & Islam, M. R. (2016). Seroprevalence of Peste des petits ruminant virus specific antibody in goats in different regions of Bangladesh. Journal of Advanced Veterinary and Animal Research, 3(2), 127–133. 10.5455/javar.2016.c140

[vms3300-bib-0098] Islam, M. M. , Kamal, A. H. M. , & Ali, M. Z. (2018). Prevalence of Peste des petits ruminants (PPR) in goat in Sylhet District, Bangladesh. International Journal of Biosciences, 13(6), 102–108.

[vms3300-bib-0099] Islam, M. , Khan, M. , Kader, H. , Begum, M. , & Asgar, M. (2012). Prevalence of PPR of goat and their response to antibiotic treatment at Mirzaganj Upazila of Patuakhali Distrtict. Journal of Environmental Science and Natural Resources, 5(2), 181–184. 10.3329/jesnr.v5i2.14811

[vms3300-bib-0100] Islam, S. S. , Rao, S. , Akhter, A. T. , Hossain, M. M. , Islam, M. R. , & Islam, S. S. (2015). Investigation of Peste des petits ruminants outbreaks in goat farms of Chuadanga District of Bangladesh in 2014. Asian Journal of Medical and Biological Research, 1(3), 434–441. 10.3329/ajmbr.v1i3.26449

[vms3300-bib-0101] Jaisree, S. , Hemalatha, S. , Muthuramalingam, T. , Manimaran, K. , Mahaprabhu, R. , & Gnanaraj, P. T. (2017). Investigation on outbreak of Peste des petits ruminants (PPR) in an organized farm among Tellicherry. Breed of Goats International Journal of Livestock Research., 7(1), 100–106.

[vms3300-bib-0102] Jalees, M. M. , Hussain, I. , Arshad, M. , Mohammad, G. , & Khan, Q. M. (2016). Seroprevalence and molecular detection of Peste des petits ruminants virus (PPRV) in different breeds of sheep and goat of Punjab (Pakistan) and its status in gravid animals. Pakistan Journal of Life and Social Sciences, 14, 12–17.

[vms3300-bib-0103] Jalees, M. M. , Hussain, I. , Arshad, M. , Muhammad, G. , Khan, Q. M. , & Mahmood, M. S. (2013). Occurrence of Peste des petitis ruminants in five districts of Punjab, Pakistan. Pakistan Veterinary Journal, 33(2), 165–169.

[vms3300-bib-0104] Janus, A. , Tresamol, P. , Saseendranath, M. , Vijayakumar, K. , & Pillai, U. N. (2009). Seroprevalence of PPR in goats in Kerala by cELISA. Journal of Veterinary and Animal Science, 40, 15–16.

[vms3300-bib-0105] Kabir, M. E. , Hossain, M. M. , Ershaduzzaman, M. , Yousuf, M. A. , & Islam, M. R. (2016). Sero‐surveillance and sero‐monitoring of locally produced PPR vaccine in the field and experimental level. Asian Journal of Medical and Biological Research, 2(1), 33–37. 10.3329/ajmbr.v2i1.27566

[vms3300-bib-0106] Kabir, M. , Reza, M. , Razi, K. , Parvez, M. , Bag, M. , & Mahfuz, S. (2010). A report on clinical prevalence of diseases and disorders in cattle and goat at the Upazilla Veterinary Hospital, Ulipur, Kurigram. International Journal of Biological Research, 2(11), 17–23.

[vms3300-bib-0107] Karam, A. , Puro, K. , Das, S. , Shakuntala, I. , Sanjukta, R. , & Milton, A. (2018). Seroprevalence of Peste des petits ruminants and bluetongue in goat population of Meghalaya, India. Veterinary World, 12, 1689–1691. 10.14202/vetworld.2018.1689-1691 PMC6362336 30774259

[vms3300-bib-0108] Kardjadj, M. , Ben‐Mahdi, M.‐H. , & Luka, P. D. (2015). First serological and molecular evidence of PPRV occurrence in Ghardaïa district, center of Algeria. Tropical Animal Health and Production, 47(7), 1279–1284. 10.1007/s11250-015-0860-1 26017753

[vms3300-bib-0109] Karlewad, V. , Bhikane, A. , Ambore, B. , & Awaz, K. (2007). Epidemiological observations on Peste des petits ruminants in Osmanabadi goats in Maharashtra. Veterinary Practitioner, 8(1), 92–93.

[vms3300-bib-0110] Kgotlele, T. , Kasanga, C. J. , Kusiluka, L. J. , & Misinzo, G. (2014). Preliminary investigation on presence of Peste des petits ruminants in Dakawa, Mvomero district, Morogoro region, Tanzania. Onderstepoort Journal of Veterinary Research, 81(2), 1–3. 10.4102/ojvr.v81i2.732 25134174

[vms3300-bib-0111] Kgotlele, T. , Macha, E. , Kasanga, C. , Kusiluka, L. , Karimuribo, E. , & Van Doorsselaere, J. (2014). Partial genetic characterization of Peste des petits ruminants virus from goats in northern and eastern Tanzania. Transboundary and Emerging Diseases, 61, 56–62. 10.1111/tbed.12229 25135464 PMC4260210

[vms3300-bib-0112] Kgotlele, T. , Torsson, E. , Kasanga, C. , Wensman, J. J. , & Misinzo, G. (2016). Seroprevalence of Peste des petits ruminants virus from samples collected in different regions of Tanzania in 2013 and 2015. Journal of Veterinary Science and Technology, 6(6), e1000394 10.4172/2157-7579.1000394

[vms3300-bib-0113] Khan, H. A. , Siddique, M. , Abubakar, M. , Arshad, M. J. , & Hussain, M. (2008). Prevalence and distribution of Peste des petits ruminants virus infection in small ruminants. Small Ruminant Research, 79(2–3), 152–157. 10.1016/j.smallrumres.2008.07.021

[vms3300-bib-0114] Khan, H. , Siddique, M. , Arshad, M. , Khan, Q. , & Rehman, S. (2007). Sero‐prevalence of Peste des petits ruminants (PPR) virus in sheep and goats in Punjab province of Pakistan. Pakistan Veterinary Journal, 27(3), 109–112.

[vms3300-bib-0115] Khaskheli, A. A. , Khaskheli, M. I. , Khaskheli, A. , Khaskheli, G. , Abro, R. , & Barham, G. S. (2017). Clinical prevalence of Peste des petits ruminants (PPR) disease in small ruminants at the urban areas of Hyderabad, Sindh. Journal of Basic and Applied Sciences, 13, 281–286. 10.6000/1927-5129.2017.13.46

[vms3300-bib-0116] Kihu, S. M. , Gachohi, J. M. , Ndungu, E. K. , Gitao, G. C. , Bebora, L. C. , & John, N. M. (2015). Sero‐epidemiology of Peste des petits ruminants virus infection in Turkana County, Kenya. BMC Veterinary Research, 11(1), 87 10.1186/s12917-015-0401-1 25888990 PMC4396631

[vms3300-bib-0117] Kihu, S. M. , Gitao, G. C. , Bebora, L. C. , John, N. M. , Wairire, G. G. , & Maingi, N. (2015). Economic losses associated with Peste des petits ruminants in Turkana County Kenya. Pastoralism, 5(1), 9. 10.1186/s12917-015-0401-1 PMC4396631 25888990

[vms3300-bib-0118] Krishna, V. , Rao, M. S. , & Shaila, M. (2001). Neutralizing antibodies to Peste‐des‐petits ruminants virus in small ruminants in Andhra Pradesh‐A serological survey. The Indian Journal of Animal Sciences, 71(3), 228–230.

[vms3300-bib-0119] Kumar, P. , Sinha, B. S. , Roy, R. K. , Kumari, R. R. , & Kumar, A. (2017). Peste des petits ruminants in goats: Sero‐epidemiological study in middle Indo‐Gangetic plains. Indian Journal of Animal Sciences, 87(4), 418–421.

[vms3300-bib-0120] Kwiatek, O. , Ali, Y. H. , Saeed, I. K. , Khalafalla, A. I. , Mohamed, O. I. , & Obeida, A. A. (2011). Asian lineage of Peste des petits ruminants virus, Africa. Emerging Infectious Diseases, 17(7), 1223 10.3201/eid1707.101216 21762576 PMC3381390

[vms3300-bib-0121] Lawal, A. , Lasisi, O. , Emikpe, B. , & Ogundipe, G. (2011). Outbreak of Peste des petits ruminants in West African Dwarf goats in Eruwa, Southwestern Nigeria. Nigerian Veterinary Journal, 32(4), 331–335.

[vms3300-bib-0122] Lembo, T. , Oura, C. , Parida, S. , Hoare, R. , Frost, L. , & Fyumagwa, R. (2013). Peste des petits ruminants infection among cattle and wildlife in northern Tanzania. Emerging Infectious Diseases, 19(12), 2037. 24274684 10.3201/eid1912.130973 PMC3840886

[vms3300-bib-0123] Li, J. , Li, L. , Wu, X. , Liu, F. , Zou, Y. , & Wang, Q. (2017). Diagnosis of Peste des petits ruminants in wild and domestic animals in Xinjiang, China, 2013–2016. Transboundary and Emerging Diseases, 64(6), e43–e47. 28101989 10.1111/tbed.12600

[vms3300-bib-0124] Lucky, N. S. , Hossain, M. K. , Roy, A. C. , Haque, M. M. , Uddin, A. M. , & Islam, M. M. (2016). A longitudinal study on clinical diseases and disorders of cattle and goats in Sylhet, Bangladesh. Journal of Advanced Veterinary and Animal Research, 3(1), 24–37. 10.5455/javar.2016.c128

[vms3300-bib-0125] Lundervold, M. , Milner‐Gulland, E. J. , O'Callaghan, C. J. , Hamblin, C. , Corteyn, A. , & Macmillan, A. P . (2004). A serological survey of ruminant livestock in Kazakhstan during post‐Soviet transitions in farming and disease control. Acta Veterinaria Scandinavica, 45(4), 211 10.1186/1751-0147-45-211 15663081 PMC1820992

[vms3300-bib-0126] Luther, N. , Umoh, J. , Majiyagbe, K. , Shamaki, D. , Nwosuh, D. , & Dogo, G. (2005). Studies on the prevalence of antibodies to Peste des petits ruminants virus (PPRV) among goats in Bauchi state. Nigerian Veterinary Journal, 27(1), 17–22.

[vms3300-bib-0127] Maganga, G. D. , Verrier, D. , Zerbinati, R. M. , Drosten, C. , Drexler, J. F. , & Leroy, E. M. (2013). Molecular typing of PPRV strains detected during an outbreak in sheep and goats in south‐eastern Gabon in 2011. Virology Journal, 10(1), 82 10.1186/1743-422X-10-82 23497402 PMC3599724

[vms3300-bib-0128] Mahajan, S. , Agrawal, R. , Kumar, M. , Mohan, A. , & Pande, N. (2012). Risk of seroconversion to Peste des petits ruminants (PPR) and its association with species, sex, age and migration. Small Ruminant Research, 104(1‐3), 195–200. 10.1016/j.smallrumres.2011.10.009

[vms3300-bib-0129] Mahajan, S. , Agrawal, R. , Kumar, M. , Mohan, A. , & Pande, N. (2013). Incidence of Peste des petits ruminants in nomadic sheep and goat of Jammu region. Veterinary World, 6(7), 384–387. 10.5455/vetworld.2013.384-387

[vms3300-bib-0130] Mahamat, O. , Doungous, T. , Kebkiba, B. , Oumar, H. A. , Oussiguéré, A. , & Yacoub, A. H. (2018). Seroprevalence, geographical distribution, and risk factors of Peste des petits ruminants in the Republic of Chad. Journal of Advanced Veterinary and Animal Research, 5(4), 420–425. 10.5455/javar.2018.e293 31453152 PMC6702906

[vms3300-bib-0131] Mahapatra, M. , Sayalel, K. , Muniraju, M. , Eblate, E. , Fyumagwa, R. , & Shilinde, S. (2015). Spillover of Peste des petits ruminants virus from domestic to wild ruminants in the Serengeti ecosystem, Tanzania. Emerging Infectious Diseases, 21(12), 2230–2234. 10.3201/eid2112.150223 26583961 PMC4672450

[vms3300-bib-0132] Mahmoud, A. , Abdellatif, M. , & Abdalla, A. (2017). High seroprevalence of PPRV‐antibodies among sheep and goats in Hail, Saudi Arabia. Veterinary Sciences: Research and Reviews., 3(1), 1–5. 10.17582/journal.vsrr/2017.3.1.1.5

[vms3300-bib-0133] Mahmoud, A. Z. , Abdellatif, M. , & Shazali, L. (2016). Prevalence of PPR‐virus antibodies in sheep, goats and camels in Hail, Saudi Arabia. British Journal of Virology, 3(3), 86 10.17582/journal.bjv/2016.3.3s.86.89

[vms3300-bib-0134] Mahmoud, M.‐A.‐E.‐F. , Elbayoumy, M. K. , Sedky, D. , & Ahmed, S. (2017). Serological investigation of some important RNA viruses affecting sheep and goats in Giza and Beni‐Suef governorates in Egypt. Veterinary World, 10(10), 1161–1166. 10.14202/vetworld.2017.1161-1166 29184360 PMC5682259

[vms3300-bib-0135] Mahmoud, M. , & Galbat, S. (2017). Outbreak of foot and mouth disease and Peste des petits ruminants in sheep flock imported for immediate slaughter in Riyadh. Veterinary World, 10(2), 238–243. 10.14202/vetworld.2017.238-243 28344409 PMC5352851

[vms3300-bib-0136] Maitlo, A. , Ujan, J. , Ujjan, S. , Ruk, M. , Memon, B. , & Mahar, A. (2017). Screening of Peste des petits ruminants virus in a population of district Khairpur, Pakistan. Genetics and Molecular Research, 16(3), 10.4238/gmr16039679 28973722

[vms3300-bib-0137] Mariner, J. C. , Jones, B. A. , Rich, K. M. , Thevasagayam, S. , Anderson, J. , & Jeggo, M. (2016). The opportunity to eradicate Peste des petits ruminants. The Journal of Immunology, 196(9), 3499–3506. 27183645 10.4049/jimmunol.1502625

[vms3300-bib-0138] Mbyuzi, A. O. , Komba, E. V. , Kimera, S. I. , & Kambarage, D. M. (2014). Sero‐prevalence and associated risk factors of Peste des petits ruminants and contagious caprine pleuro‐pneumonia in goats and sheep in the Southern Zone of Tanzania. Preventive Veterinary Medicine, 116(1‐2), 138–144. 10.1016/j.prevetmed.2014.06.013 25022914

[vms3300-bib-0139] Mebrahtu, K. , Getachew, S. , Tesfaye, T. , Sahlu, E. , & Aragaw, K. (2018). Sero‐epidemiological study of Peste des petits ruminants (PPR) in sheep and goats under different production systems in South Omo, southern Ethiopia. Small Ruminant Research, 169, 90–93. 10.1016/j.smallrumres.2018.06.017

[vms3300-bib-0140] Megersa, B. , Biffa, D. , Belina, T. , Debela, E. , Regassa, A. , & Abunna, F. (2011). Serological investigation of Peste des petits ruminants (PPR) in small ruminants managed under pastoral and agro‐pastoral systems in Ethiopia. Small Ruminant Research, 97(1–3), 134–138. 10.1016/j.smallrumres.2011.03.003

[vms3300-bib-0141] Meher, M. , Afrin, M. , Hassan, Z. , & Alam, J. (2017). Epidemiological investigation of Peste des petits ruminants virus infection in goat with therapeutic managementat at Bera upazila of Pabna in Bangladesh. Progressive Agriculture, 28(2), 114–119. 10.3329/pa.v28i2.33472.

[vms3300-bib-0142] Mehmood, A. , Ali, Q. , Gadahi, J. A. , Malik, S. A. , & Shah, S. I. (2009). Detection of Peste des petits ruminants (PPR) virus antibodies in sheep and goat populations of the North West Frontier Province (NWFP) of Pakistan by competitive ELISA (cELISA). Veterinary World, 2(9), 333–336.

[vms3300-bib-0143] Milind, M. , Bincy Joseph, D. K. , Sharma, D. K. , Gaurav, A. , Sharma, M. C. , & Prakash, C. (2018). Status of Peste des petits ruminants in small ruminants of semi arid regions of Rajasthan. International Journal of Current Microbiology and Applied Sciences, 7(12), 1217–1224. 10.20546/ijcmas.2018.712.152

[vms3300-bib-0144] Mohanto, J. K. , Hoque, M. F. , & Juli, M. S. B. (2018). Prevalence of Peste des petits ruminants (PPR) in goat and their response to antibiotic treatment at Gangachara upazila of Rangpur district. Journal of Agricultural and Rural Research, 2(3), 30–37.

[vms3300-bib-0145] Mostafa, S. A. E. T. (2012). Sero‐prevalence and risk factors of Peste des petits ruminant (ppr) in sheep in River Nile and White Nile states, Sudan. Journal of Veterinary Medicine and Animal Production, 3(2), 106–126.

[vms3300-bib-0146] Moumin, G. , Moussa, C. , Teshale, S. , & Gezahegne, M. (2018). Seroprevalence and risk factors for Peste des petits ruminants in sheep and goats in Djibouti. Revue Scientifique Et Technique De l'OIE, 37(3), 961–969. 10.20506/37.3.2899 30964456

[vms3300-bib-0147] Muhsen, R. (2013). Seroepidemiology of PPR in goats in Basrah province. AL‐Qadisiyah Journal of Veterinary Medicine Sciences, 12(1), 139–143. 10.29079/vol12iss1art242

[vms3300-bib-0148] Mulindwa, B. , Ruhweza, S. P. , Ayebazibwe, C. , Mwiine, F. N. , Muhanguzi, D. , & Olaho‐Mukani, W. (2011). Peste des petits ruminants serological survey in Karamoja sub region of Uganda by competitive ELISA. Veterinary World, 4(4), 149–152. 10.5455/vetworld.2011.149-152

[vms3300-bib-0149] Munir, M. , Shah, S. H. , Shabbir, M. Z. , & Berg, M. (2013). Peste des petits ruminants in Pakistan. Journal of Infection and Molecular Biology, 1(4), 64–66.

[vms3300-bib-0150] Munir, M. , Siddique, M. , Shehzad, A. , Zohari, S. , & Stahl, K. (2010). Seroprevalence of antibodies to Peste des petits ruminants at various governmental livestock farms of Punjab, Pakistan. Asian Journal of Epidemiology, 3(3), 183–191.

[vms3300-bib-0151] Muse, E. , Matondo, R. , Karimuribo, E. D. , Misinzo, G. , Albano, M. O. , & Gitao, G. C. (2012). Clinico‐pathological findings of the 2011 outbreak of Peste des petits ruminants (PPR) in Tandahimba district, southern Tanzania. Research Opinion in Animal and Veterinary Sciences, 2(4), 256–262.

[vms3300-bib-0152] Nabi, R. , Hossain, M. S. , Saha, S. , Alam, J. , & Giasuddin, M. (2018). Molecular epidemiology of Peste des petits ruminants (PPR) in goat. International Journal of Scientific and Technology Research, 7(3), 7–12.

[vms3300-bib-0153] Nath, T. C. , Bhuiyan, M. J. U. , Mamun, M. , Datta, R. , Chowdhury, S. , & Hossain, M. (2014). Common infectious diseases of goats in Chittagong district of Bangladesh. International Journal of Scientific Research in Agricultural Sciences, 1(3), 43–49. 10.12983/ijsras-2014-p0043-0049

[vms3300-bib-0154] Naznin, M. , Ahaduzzaman, M. , Chowdhury, S. , & Biswas, P. (2014). Prevalence and clinico‐pathological parameters of PPR infected goats and their response to antibiotic treatment at Panchlaish, Chittagong, Bangladesh. International Journal of Natural Sciences., 4(2), 1–7.

[vms3300-bib-0155] Nizamani, A. , Nizamani, Z. , Umrani, A. , Dewani, P. , Vandiar, M. , & Gandahi, J. (2015). Prevalence of Peste des petits ruminants virus antibodies in small Ruminantsin Sindh, Pakistan. The Journal of Animal & Plant Sciences, 25(6), 1515–1529.

[vms3300-bib-0156] Nwobodo, H. , Ezeifeka, G. , Ezejiofor, C. , & Onyianta, O. (2013). Seroprevalence of Peste des petits ruminants among goats and sheep in Enugu State of Nigeria. Animal Health and Production, 61, 613–616.

[vms3300-bib-0157] Opasina, B. (1985). Disease constraints on village goat production in southwest Nigeria. Revue D’élevage Et De Médecine Vétérinaire Des Pays Tropicaux, 38(3), 284–294. 3841965

[vms3300-bib-0158] Opasina, B. , & Putt, S. (1985). Outbreaks of Peste des petits ruminants in village goat flocks in Nigeria. Tropical Animal Health and Production, 17(4), 219–224. 10.1007/BF02356980 4089968

[vms3300-bib-0159] Oshiek, A. , Abdelkadir, M. , Mihreteab, B. , Mengesha, S. , Teklay, G. , & Yemane, H. (2018). Investigating Peste des petits ruminants (PPR) in naturally infected goats and sheep in Anseba Region, Eritrea, by reverse transcription polymerase chain reaction (RT‐PCR). Tropical Animal Health and Production, 50(4), 915–920. 10.1007/s11250-018-1511-0 29374823

[vms3300-bib-0160] Osman, N. A. , Ibrahim, H. M. , Osman, A. A. , Alnour, R. M. , & Eldin, O. A. G. (2018). Sero‐prevalence of Peste des petits ruminants virus antibodies in sheep and goats from the Sudan, 2016–2017. Virus Disease, 29(4), 531–536. 10.1007/s13337-018-0496-7 30539057 PMC6261903

[vms3300-bib-0161] Otsyina, H. , Arthur, C. , Ayim‐Akunnor, M. , & Obese, F. (2013). Sero‐prevalence of Pestes des petits ruminant (PPR) in sheep, goats and cattle in Ghana. Bulletin of Animal Health and Production in Africa, 61(3), 473–479.

[vms3300-bib-0162] Özkul, A. , Akca, Y. , Alkan, F. , Barrett, T. , Karaoglu, T. , & Dagalp, S. B. (2002). Prevalence, distribution, and host range of Peste des petits ruminants virus, Turkey. Emerging Infectious Diseases, 8(7), 709. 10.3201/eid0807.010471 PMC2730320 12095439

[vms3300-bib-0163] Ozmen, O. , Kale, M. , Haligur, M. , & Yavru, S. (2009). Pathological, serological, and virological findings in sheep infected simultaneously with Bluetongue, Peste‐des‐petits‐ruminants, and Sheeppox viruses. Tropical Animal Health and Production, 41(6), 951–958. 10.1007/s11250-008-9284-5 19067219

[vms3300-bib-0164] Parida, S. , Muniraju, M. , Altan, E. , Baazizi, R. , Raj, G. D. , & Mahapatra, M. (2016). Emergence of PPR and its threat to Europe. Small Ruminant Research, 142, 16–21. 27695194 10.1016/j.smallrumres.2016.02.018 PMC5035059

[vms3300-bib-0165] Parvez, M. A. , Khatun, R. , & Al Noman, M. A. (2014). Prevalence and associated risk factors of Peste des petits ruminants (PPR) in goats in Chittagong district, Bangladesh. Research Journal for Veterinary Practitioners, 2(1s), 14–17. 10.14737/journal.rjvp/2014/2.1s.14.17

[vms3300-bib-0166] Patil, S. , Raghavendra, A. , Gajendragad, M. , Bhure, S. , Sengupta, P. , & Tiwari, C. (2009). Sero‐prevalence of Peste‐des‐Petits ruminants in small ruminants in Karnataka. Indian Veterinary Journal, 86(2), 118–119.

[vms3300-bib-0167] Poddar, S. , Tuli, D. , Sultana, J. , Akter, S. , & Alauddin, M. (2018). Prevalence of Peste des petits ruminants in Goat at Upizalla Veterinary Hospital, Pirojpur Sadar, Bangladesh. Turkish Journal of Veterinary Research. 2(1), 5–8.

[vms3300-bib-0168] Raghavendra, A. , Gajendragad, M. , Sengupta, P. , Patil, S. , Tiwari, C. , & Balumahendiran, M. (2008). Seroepidemiology of Peste des petits ruminants in sheep and goats of southern peninsular India. Revue Scientifique Et Technique De l'OIE, 27(3), 861–867. 10.20506/rst.27.3.1838 19284053

[vms3300-bib-0169] Rahman, M. M. , Alam, K. J. , Alam, M. S. , Hasan, M. M. , & Moonmoon, M. (2016). A study on prevalence of Peste des petits ruminant (PPR) in goat at Bagmara upazilla at Rajshahi district in Bangladesh. Research in Agriculture Livestock and Fisheries, 3(2), 339–344. 10.3329/ralf.v3i2.29362

[vms3300-bib-0170] Rahman, M. Z. , Haider, N. , Gurley, E. S. , Ahmed, S. , Osmani, M. G. , & Hossain, M. B. (2018). Epidemiology and genetic characterization of Peste des petits ruminants virus in Bangladesh. Veterinary Medicine and Science, 4(3), 161–171. 10.1002/vms3.98 PMC6090418 29663718

[vms3300-bib-0171] Rahman, M. M. , Hassan, M. Z. , Sultana, S. , Uddin, M. K. , & Hossain, S. S. (2017). Incidence of Peste des petits ruminants in Rangpur sadar of Bangladesh. Asian Journal of Medical and Biological Research, 3(4), 529–533. 10.3329/ajmbr.v3i4.35345

[vms3300-bib-0172] Rahman, M. , Hossain, M. , Ahsan, M. , Khokon, M. , & Kibria, A. (2011). Prevalence of PPR and its effective treatment in goats of Pabna district of Bangladesh. International Journal of Aquaculture and Fishery Sciences, 4, 418–422.

[vms3300-bib-0173] Rahman, M. , Shadmin, I. , Noor, M. , Parvin, R. , Chowdhury, E. , & Islam, M. (2011). Peste des petits ruminants virus infection of goats in Bangladesh: Pathological investigation, molecular detection and isolation of the virus. Bangladesh Veterinarian, 28(1), 1–7. 10.3329/bvet.v28i1.8808

[vms3300-bib-0174] Rakshit, N. , Paul, A. , Amin, M. , Asaduzzaman, M. , Sen, P. , & Talukder, M. (2015). Occurrence and therapeutic response of Peste des petits ruminants (PPR) in goats at the selected southern part of Bangladesh. Wayamba Journal of Animal Science, 7, 1239–1243.

[vms3300-bib-0175] Rashid, A. , Asim, M. , & Hussain, A. (2008). Seroprevalence of Peste des petits ruminants (PPR) virus in goats, sheep and cattle at livestock production research institute Bahadurnagar Okara. Journal of Animal and Plant Sciences, 18(4), 114–116.

[vms3300-bib-0176] Rony, M. , Rahman, A. , Alam, M. , Dhand, N. , & Ward, M. (2017). Peste des petits ruminants risk factors and space‐time clusters in Mymensingh, Bangladesh. Transboundary and Emerging Diseases, 64(6), 2042–2048. 10.1111/tbed.12615 28109070

[vms3300-bib-0177] Saeed, F. A. , Abdel‐Aziz, S. A. , & Gumaa, M. M. (2018). Seroprevalence and associated risk factors of Peste des petits ruminants among sheep and goats in Kassala state, Sudan. Open Journal of Animal Sciences, 8(04), 381 10.4236/ojas.2018.84029

[vms3300-bib-0178] Saeed, I. K. , Ali, Y. H. , Khalafalla, A. I. , & Rahman‐Mahasin, E. (2010). Current situation of Peste des petits ruminants (PPR) in the Sudan. Tropical Animal Health and Production, 42(1), 89–93. 10.1007/s11250-009-9389-5 19548103

[vms3300-bib-0179] Sağlam, Y. , & Temur, A. (2009). Immunohistochemical detection of Peste des petits ruminants (PPR) viral antigen from the cases of naturally occurring pneumonia in sheep. Kafkas Universitesi Veteriner Fakultesi Dergisi, 15(3), 423–428.

[vms3300-bib-0180] Salih, H. A. M. , Elfadil, A. A. M. , Saeed, I. K. , & Ali, Y. H. (2014). Seroprevalence and risk factors of Peste des Petits Ruminants in sheep and goats in Sudan. Journal of Advanced Veterinary and Animal Research, 1(2), 42–49. 10.5455/javar.2014.a12

[vms3300-bib-0181] Sande, R. , Ayebazibwe, C. , Waiswa, C. , Ejobi, F. , Mwiine, F. N. , & Olaho‐Mukani, W. (2011). Evidence of Peste des petits ruminants virus antibodies in small ruminants in Amuru and Gulu districts, Uganda. Pakistan Veterinary Journal, 31(4), 363–365.

[vms3300-bib-0182] Sannat, C. , Chandel, B. , Chauhan, H. , & Dadawala, A. (2011). Seroprevalence of PPR in sheep and goats of North Gujarat. Indian Journal of Small Ruminants, 17(1), 118–121.

[vms3300-bib-0183] Saravanan, P. , Balamurugan, V. , Sen, A. , Sarkar, J. , Sahay, B. , & Rajak, K. . (2007). Mixed infection of Peste des petits ruminants and orf on a goat farm in Shahjahanpur, India. vol. 160. Veterinary Record, 160(12), 410–412. 10.1136/vr.160.12.410 17384295

[vms3300-bib-0184] Saritha, G. , Shobhamani, B. , Rajak, K. , & Sreedevi, B. (2015). Detection and confirmation of PPR virus antigen in sheep and goats by sandwich‐ELISA and RT‐PCR in Andhra Pradesh, India. Journal of Advanced Veterinary and Animal Research, 2(2), 210–222. 10.5455/javar.2015.b71

[vms3300-bib-0185] Saritha, G. , Shobhamani, B. , & Sreedevi, B. (2014). Sero‐prevalence of Peste des petits ruminants in pastoral small ruminants with special reference on sensitivity to age and agro‐climatic zone. Animal Science, 8(3), 103–107.

[vms3300-bib-0186] Sarker, S. , & Islam, M. H. (2011). Prevalence and risk factor assessment of Peste des petits ruminants in goats in Rajshahi, Bangladesh. Veterinary World, 546 10.5455/vetworld.2011.546-549

[vms3300-bib-0187] Şevik, M. , & Sait, A. (2015). Genetic characterization of Peste des petits ruminants virus, Turkey, 2009–2013. Research in Veterinary Science, 101, 187–195. 10.1016/j.rvsc.2015.05.005 26022069

[vms3300-bib-0188] Sharma, C. , Mehta, H. , Prakash, M. , & Shukla, P. (2012). Studies on clinico‐haemato‐biochemical changes in Peste des petits ruminants in goats. Veterinary Practitioner, 13(2), 322–355.

[vms3300-bib-0189] Sharma, C. , Shrivastava, M. , Mehta, H. , & Shukla, P. (2012). Studies on incidence of Peste des petits ruminants in goats of Indore district of Madhya Pradesh. Veterinary Practitioner, 13(1), 16–18.

[vms3300-bib-0190] Shukla, N. , Singh, K. , & Hirpurkar, S. (2008). Seroprevalence of Peste des petits ruminants in sheep in Chhattisgarh. The Indian Journal of Small Ruminants, 14(1), 131–132.

[vms3300-bib-0191] Siddiqui, M. , Ahasan, A. , Islam, N. , Kundu, P. , Munshi, M. , & Chowdhury, E. (2014). Peste des petits ruminants (PPR) virus antibodies in goats and cattle of the Saint Martin s Island in Bangladesh. Bangladesh Veterinarian, 31(2), 55–59. 10.3329/bvet.v31i2.27685

[vms3300-bib-0192] Singh, B. , Bardhan, D. , Verma, M. , Prasad, S. , & Sinha, D. (2014). Estimation of economic losses due to Peste de petits ruminants in small ruminants in India. Veterinary World, 7(4), 194–199.

[vms3300-bib-0193] Singh, D. , Malik, Y. S. , Sharma, K. , & Kuldeep, D. (2015). Identification and phylogenetic analysis of Peste des petits ruminants (PPR) virus Isolates from India. Asian Journal of Animal and Veterinary Advances, 10(8), 386–393. 10.3923/ajava.2015.386.393

[vms3300-bib-0194] Singh, R. , Saravanan, P. , Sreenivasa, B. , Singh, R. , & Bandyopadhyay, S. (2004). Prevalence and distribution of Peste des petits ruminants virus infection in small ruminants in India. Revue Scientifique Et Technique, 23(3), 807–819. 10.20506/rst.23.3.1522 15861876

[vms3300-bib-0195] Singh, S. , Jindal, N. , Nain, S. , & Khokhar, R. (2006). Seroprevalence of Peste des petits ruminants in sheep and goats in and around Haryana state. Haryana Veterinarian, 45, 11–14.

[vms3300-bib-0196] Soltan, M. A. , & Abd‐Eldaim, M. M. (2014). Emergence of Peste des petits ruminants virus lineage IV in Ismailia Province, Egypt. Infection, Genetics and Evolution, 28, 44–47. 10.1016/j.meegid.2014.08.016 25200722

[vms3300-bib-0197] Sundufu, A. J. , Ansumana, R. , Bockarie, A. S. , Bangura, U. , Lamin, J. M. , & Jacobsen, K. H. (2015). Syndromic surveillance of Peste des petits ruminants and other animal diseases in Koinadugu district, Sierra Leone, 2011–2012. Tropical Animal Health and Production, 47(2), 473–477. 10.1007/s11250-014-0736-9 25433648

[vms3300-bib-0198] Swai, E. S. , Kapaga, A. , Kivaria, F. , Tinuga, D. , Joshua, G. , & Sanka, P. (2009). Prevalence and distribution of Peste des petits ruminants virus antibodies in various districts of Tanzania. Veterinary Research Communications, 33(8), 927–936. 10.1007/s11259-009-9311-7 19705291

[vms3300-bib-0199] Taylor, W. , Al Busaidy, S. , & Barrett, T. (1990). The epidemiology of Peste des petits ruminants in the Sultanate of Oman. Veterinary Microbiology, 22(4), 341–352. 10.1016/0378-1135(90)90021-M 2114052

[vms3300-bib-0200] Thombare, N. , & Sinha, M. K. (2009). Economic implications of Peste des petits ruminants (PPR) disease in sheep and goats: A sample analysis of district Pune, Maharastra. Agricultural Economics Research Review, 22, 319–322.

[vms3300-bib-0201] Torsson, E. , Berg, M. , Misinzo, G. , Herbe, I. , Kgotlele, T. , & Päärni, M. (2017). Seroprevalence and risk factors for Peste des petits ruminants and selected differential diagnosis in sheep and goats in Tanzania. Infection Ecology & Epidemiology, 7(1), 1368336 10.1080/20008686.2017.1368336 29081918 PMC5645728

[vms3300-bib-0202] Truong, T. , Boshra, H. , Embury‐Hyatt, C. , Nfon, C. , Gerdts, V. , & Tikoo, S. (2014). Peste des petits ruminants virus tissue tropism and pathogenesis in sheep and goats following experimental infection. PLoS One, 9(1), e87145 10.1371/journal.pone.0087145. 24498032 PMC3907444

[vms3300-bib-0203] Undrakhbayar, T. , Uuganbayar, E. , & Odbileg, R. (2016). Sero‐surveillance of “Peste des petits ruminants” PPR in Mongolia and development of recommendation. Mongolian Journal of Agricultural Sciences, 19(3), 22–26. 10.5564/mjas.v19i3.731

[vms3300-bib-0204] Wang, Z. , Bao, J. , Wu, X. , Liu, Y. , Li, L. , & Liu, C. (2009). Peste des petits ruminants virus in Tibet, China. Emerging Infectious Diseases, 15(2), 299–301. 10.3201/eid1502.080817 19193278 PMC2657621

[vms3300-bib-0205] Waret‐Szkuta, A. , Roger, F. , Chavernac, D. , Yigezu, L. , Libeau, G. , & Pfeiffer, D. U. (2008). Peste des petits ruminants (PPR) in Ethiopia: Analysis of a national serological survey. BMC Veterinary Research, 4(1), 34 10.1186/1746-6148-4-34 18786275 PMC2561016

[vms3300-bib-0206] Yapici, O. , Bulut, O. , Avci, O. , Kale, M. , Tursumbetov, M. , & Yavru, S. (2014). First report on seroprevalence of Bluetongue, Border disease and Peste des petits ruminants virus infections in sheep in Kyrgyzstan. Indian Journal of Animal Research, 48(5), 469–472. 10.5958/0976-0555.2014.00013.2

[vms3300-bib-0207] Yener, Z. , Sağlam, Y. , Temur, A. , & Keleş, H. (2004). Immunohistochemical detection of Peste des petits ruminants viral antigens in tissues from cases of naturally occurring pneumonia in goats. Small Ruminant Research, 51(3), 273–277. 10.1016/S0921-4488(03)00194-9

[vms3300-bib-0208] Yilmaz, V. (2016). Molecular detection of Peste des petits ruminants virus from different sample materials of sheep, Northeastern Turkey. Advances in Animal and Veterinary Sciences, 4(3), 169–173. 10.14737/journal.aavs/2016/4.3.169.173

[vms3300-bib-0209] Yousuf, M. A. , Giasuddin, M. , Islam, S. S. , & Islam, M. R. (2015). Management of an outbreak of Peste des petits ruminants with antibiotic combined hyperimmune serum therapy. Asian Journal of Medical and Biological Research, 1(2), 230–234. 10.3329/ajmbr.v1i2.25616

[vms3300-bib-0210] Yousuf, M. A. , Rahman, M. M. , Alauddin, M. , Rahman, S. B. , Islam, S. S. , & Islam, M. R. (2017). Sero‐surveillance of Peste des petits ruminant viral antibody in goats at different areas of Bangladesh. Asian Journal of Medical and Biological Research, 3(3), 347–351. 10.3329/ajmbr.v3i3.34524

[vms3300-bib-0211] Zahur, A. , Irshad, H. , Hussain, M. , Ullah, A. , Jahangir, M. , & Khan, M. Q. (2008). The epidemiology of Peste des petits ruminants in Pakistan. Revue Scientifique Et Technique De l'OIE, 27(3), 877–884. 10.20506/rst.27.3.1848 19284055

[vms3300-bib-0212] Zahur, A. , Ullah, A. , Hussain, M. , Irshad, H. , Hameed, A. , & Jahangir, M. (2011). Sero‐epidemiology of Peste des petits ruminants (PPR) in Pakistan. Preventive Veterinary Medicine, 102(1), 87–92. 10.1016/j.prevetmed.2011.06.011 21788090

[vms3300-bib-0213] Zahur, A. , Ullah, A. , Irshad, H. , Farooq, M. , Hussain, M. , & Jahangir, M. (2009). Epidemiological investigations of a Peste des petits ruminants (PPR) outbreak in Afghan sheep in Pakistan. Pakistan Veterinary Journal, 29(4), 174–178.

[vms3300-bib-0214] Zahur, A. B. , Ullah, A. , Irshad, H. , Latif, A. , Ullah, R. W. , & Jahangir, M. (2014). Epidemiological analysis of Peste des petits ruminants (PPR) outbreaks in Pakistan. Journal of Biosciences and Medicines, 2(06), 18–26. 10.4236/jbm.2014.26004

