# Supplementary material for: Peste des petits ruminants (PPR) in Africa and Asia: A systematic review and meta‐analysis of the prevalence in sheep and goats between 1969 and 2018
Source: Vet Med Sci. 2020 Jun 12;6(4):813–33. doi: 10.1002/vms3.300 (PMC7738735; doi:10.1002/vms3.300)
Supplement: Supplementary file 2 — Appendix S2 [file VMS3-6-813-s002.docx]

**Text S1:** Quality assessment checklist

The following items were examined and given a score based on a simple scale system (1 for ''yes'', 0 for ''no'').

1. Was the research objective clearly stated?
2. Was the sampling area clearly described with reference to the location?
3. Was the period of the study stated?
4. Was the target sample a close representation of the general population?
5. Was some form of random selection used to select the samples
6. Was a minimum sample size calculated?
7. Were the sample processing and diagnostic method clearly described?
8. Were the subjects categorised by sex?
9. Were the subjects categorised by age and were the age categories clearly defined?

The quality index score for each study was calculated by dividing the study quality score by 9.

**Table S2:** Quality score and quality index score of individual contributing study

| Author | Year | Quality score | Quality index score |
| --- | --- | --- | --- |
| Abd El-Rahim et al. | 2010 | 7 | 0.78 |
| Abdalla et al. | 2012 | 7 | 0.78 |
| Abraham et al. | 2005 | 6 | 0.67 |
| Adombi et al. | 2017 | 4 | 0.44 |
| Afera et al. | 2014 | 9 | 1.00 |
| Ali et al. | 2014 | 5 | 0.56 |
| Al-Majali et al. | 2008 | 8 | 0.89 |
| Almeshay et al. | 2017 | 9 | 1.00 |
| Ameen and Ajayi | 2013 | 5 | 0.56 |
| Ayim-Akonor et al. | 2014 | 4 | 0.44 |
| Baazizi et al. | 2015 | 5 | 0.56 |
| Baazizi et al. | 2017 | 8 | 0.89 |
| Bello et al. | 2016 | 8 | 0.89 |
| Bello et al. | 2018 | 8 | 0.89 |
| Birindwa et al. | 2017 | 6 | 0.67 |
| Cêtre-Sossah et al. | 2016 | 6 | 0.67 |
| Delil et al. | 2012 | 6 | 0.67 |
| Elhaig et al. | 2018 | 6 | 0.67 |
| El-Yuguda et al. | 2009 | 4 | 0.44 |
| El-Yuguda et al. | 2009 | 6 | 0.67 |
| El-Yuguda et al. | 2013 | 7 | 0.78 |
| Enan et al. | 2013 | 6 | 0.67 |
| Ezeokolo et al. | 1986 | 6 | 0.67 |
| Faris et al. | 2012 | 8 | 0.89 |
| Farougou et al. | 2013 | 8 | 0.89 |
| Fentie et al. | 2018 | 7 | 0.78 |
| Gari et al. | 2015 | 6 | 0.67 |
| Gari et al. | 2017 | 9 | 1.00 |
| Goossens et al. | 1998 | 6 | 0.67 |
| Haroun et al. | 2002 | 4 | 0.44 |
| Intisar et al. | 2017 | 6 | 0.67 |
| Ishag et al. | 2015 | 7 | 0.78 |
| Kardjadj et al. | 2015 | 5 | 0.56 |
| Kgotlele et al. | 2014 | 3 | 0.33 |
| Kgotlele et al. | 2014 | 5 | 0.56 |
| Kgotlele et al. | 2016 | 6 | 0.67 |
| Kihu et al. | 2015 | 8 | 0.89 |
| Kwiatek et al. | 2011 | 4 | 0.44 |
| Lawal et al. | 2011 | 4 | 0.44 |
| Luther et al. | 2005 | 7 | 0.78 |
| Maganga et al. | 2013 | 4 | 0.44 |
| Mahamat et al. | 2018 | 8 | 0.89 |
| Mahapatra et al. | 2015 | 5 | 0.56 |
| Mahmoud et al. | 2017 | 6 | 0.67 |
| Mbyuzi et al. | 2014 | 6 | 0.67 |
| Mebrahtu et al. | 2018 | 7 | 0.78 |
| Megersa et al. | 2011 | 7 | 0.78 |
| Mostafa et al. | 2012 | 6 | 0.67 |
| Moumin et al. | 2018 | 7 | 0.78 |
| Mulindwa et al. | 2011 | 7 | 0.78 |
| Muse et al. | 2012 | 4 | 0.44 |
| Nwobodo et al. | 2013 | 6 | 0.67 |
| Opasina | 1985 | 4 | 0.44 |
| Opasina and Putt | 1985 | 5 | 0.56 |
| Ishag et al. | 2015 | 6 | 0.67 |
| Oshiek et al. | 2018 | 4 | 0.44 |
| Osman et al. | 2018 | 5 | 0.56 |
| Otsyina et al. | 2013 | 6 | 0.67 |
| Saeed et al. | 2010 | 6 | 0.67 |
| Saeed et al. | 2018 | 7 | 0.78 |
| Salih et al. | 2014 | 6 | 0.67 |
| Sande et al. | 2011 | 7 | 0.78 |
| Soltan and Abd-Eldaim | 2014 | 6 | 0.67 |
| Sundufu et al. | 2015 | 6 | 0.67 |
| Swai et al. | 2009 | 7 | 0.78 |
| Torsson et al. | 2017 | 7 | 0.78 |
| Waret-Szkuta et al. | 2008 | 7 | 0.78 |
| Abubakar et al. | 2008 | 6 | 0.67 |
| Abubakar et al. | 2008 | 5 | 0.56 |
| Abubakar et al. | 2008 | 5 | 0.56 |
| Abubakar et al. | 2009 | 7 | 0.78 |
| Abubakar et al. | 2011 | 7 | 0.78 |
| Abubakar et al. | 2016 | 5 | 0.56 |
| Abubakar et al. | 2017 | 6 | 0.67 |
| Abubakar et al. | 2018 | 6 | 0.67 |
| Acharya et al. | 2018 | 9 | 1.00 |
| Ahmad et al. | 2005 | 4 | 0.44 |
| Ahmed et al. | 2016 | 5 | 0.56 |
| Ahmed et al. | 2017 | 6 | 0.67 |
| AL-Afaleq et al. | 2004 | 8 | 0.89 |
| Alam et al. | 2018 | 7 | 0.78 |
| Albayrak and Alkan | 2009 | 6 | 0.67 |
| Albayrak and Gür | 2010 | 6 | 0.67 |
| Al-Dubaib | 2008 | 6 | 0.67 |
| Al-Dubaib | 2009 | 6 | 0.67 |
| Amin | 2015 | 5 | 0.56 |
| Anees et al. | 2013 | 3 | 0.33 |
| Atta-ur-Rahman | 2004 | 7 | 0.78 |
| Aytekin et al. | 2011 | 6 | 0.67 |
| Aziz-ul-Rahman et al. | 2016 | 4 | 0.44 |
| Balamurugan et al | 2011 | 7 | 0.78 |
| Balamurugan et al | 2012 | 6 | 0.67 |
| Balamurugan et al | 2014 | 6 | 0.67 |
| Balamurugan et al | 2014 | 6 | 0.67 |
| Banik et al. | 2008 | 6 | 0.67 |
| Bari et al. | 2018 | 7 | 0.78 |
| Begum et al. | 2016 | 6 | 0.67 |
| Begum et al. | 2017 | 4 | 0.44 |
| Bhanuprakash et al. | 2008 | 5 | 0.56 |
| Bhaskar et al. | 2011 | 6 | 0.67 |
| Bupasha et al. | 2015 | 8 | 0.89 |
| Chuhan et al. | 2012 | 5 | 0.56 |
| Chuhan et al. | 2014 | 5 | 0.56 |
| Chavan et al. | 2009 | 5 | 0.56 |
| Choudhary et al. | 2009 | 4 | 0.44 |
| Chowdhury et al. | 2014 | 6 | 0.67 |
| Das et al. | 2007 | 4 | 0.44 |
| De et al. | 2016 | 7 | 0.78 |
| Devi et al. | 2016 | 7 | 0.78 |
| Durrani et al. | 2010 | 6 | 0.67 |
| El-Rahim et al. | 2005 | 4 | 0.44 |
| Güler et al. | 2014 | 6 | 0.67 |
| Gurcay et al. | 2013 | 4 | 0.44 |
| Haq et al. | 2017 | 5 | 0.56 |
| Haque et al. | 2004 | 6 | 0.67 |
| Hota et al. | 2018 | 7 | 0.78 |
| Islam et al. | 2012 | 6 | 0.67 |
| Islam et al. | 2014 | 7 | 0.78 |
| Islam et al. | 2015 | 6 | 0.67 |
| Islam et al. | 2016 | 7 | 0.78 |
| Islam et al. | 2017 | 6 | 0.67 |
| Islam et al. | 2018 | 7 | 0.78 |
| Jaisree et al. | 2017 | 6 | 0.67 |
| Jalees et al. | 2013 | 7 | 0.78 |
| Jalees et al. | 2016 | 7 | 0.78 |
| Janus et al. | 2009 | 5 | 0.56 |
| Kabir et al. | 2010 | 8 | 0.89 |
| Kabir et al. | 2016 | 5 | 0.56 |
| Karam et al. | 2018 | 6 | 0.67 |
| Karlewad et al. | 2007 | 7 | 0.78 |
| Khan et al. | 2007 | 6 | 0.67 |
| Khan et al. | 2008 | 8 | 0.89 |
| Khaskheli et al. | 2017 | 6 | 0.67 |
| Krishna et al. | 2001 | 5 | 0.56 |
| Kumar et al. | 2017 | 6 | 0.67 |
| Li et al. | 2017 | 6 | 0.67 |
| Lucky et al. | 2016 | 6 | 0.67 |
| Lundervold et al | 2004 | 6 | 0.67 |
| Mahajan et al. | 2012 | 7 | 0.78 |
| Mahajan et al. | 2013 | 7 | 0.78 |
| Mahmoud et al. | 2016 | 6 | 0.67 |
| Mahmoud et al. | 2017 | 6 | 0.67 |
| Mahmoud and Galbat | 2017 | 6 | 0.67 |
| Maitlo et al. | 2017 | 5 | 0.56 |
| Meher et al. | 2017 | 6 | 0.67 |
| Mehmood et al. | 2009 | 5 | 0.56 |
| Milind et al. | 2018 | 5 | 0.56 |
| Mohanto et al. | 2018 | 8 | 0.89 |
| Muhsen | 2013 | 7 | 0.78 |
| Munir et al. | 2008 | 6 | 0.67 |
| Munir et al. | 2013 | 4 | 0.44 |
| Nabi et al. | 2018 | 7 | 0.78 |
| Nath et al. | 2014 | 8 | 0.89 |
| Naznin et al. | 2014 | 7 | 0.78 |
| Nizamani et al. | 2015 | 8 | 0.89 |
| Özkul et al. | 2002 | 6 | 0.67 |
| Ozmen et al. | 2009 | 6 | 0.67 |
| Parvez et al. | 2014 | 8 | 0.89 |
| Patil et al. | 2009 | 5 | 0.56 |
| Poddar et al. | 2018 | 8 | 0.89 |
| Raghavendra et al. | 2008 | 6 | 0.67 |
| Rahman et al. | 2011 | 7 | 0.78 |
| Rahman et al. | 2011 | 4 | 0.44 |
| Rahman et al. | 2016 | 6 | 0.67 |
| Rahman et al. | 2017 | 7 | 0.78 |
| Rahman et al. | 2018 | 6 | 0.67 |
| Rakshit et al. | 2015 | 6 | 0.67 |
| Rashid et al. | 2008 | 5 | 0.56 |
| Rony et al. | 2016 | 7 | 0.78 |
| Saglam and Temur | 2009 | 6 | 0.67 |
| Sannat et al. | 2011 | 5 | 0.56 |
| Saravanan et al. | 2007 | 4 | 0.44 |
| Saritha et al. | 2014 | 6 | 0.67 |
| Saritha et al. | 2015 | 4 | 0.44 |
| Sarker and Islam | 2011 | 7 | 0.78 |
| Şevik and Sait | 2015 | 6 | 0.67 |
| Sharma et al. | 2012 | 6 | 0.67 |
| Sharma et al. | 2012 | 4 | 0.44 |
| Shukla et al. | 2008 | 7 | 0.78 |
| Siddiqui et al. | 2014 | 6 | 0.67 |
| Singh et al. | 2004 | 6 | 0.67 |
| Singh et al. | 2006 | 6 | 0.67 |
| Singh et al. | 2015 | 5 | 0.56 |
| Taylor et al. | 1990 | 4 | 0.44 |
| Thombare and Sinha | 2009 | 6 | 0.67 |
| Undrakhbayar et al. | 2016 | 6 | 0.67 |
| Wang et al. | 2009 | 6 | 0.67 |
| Yapici et al. | 2014 | 5 | 0.56 |
| Yener et al. | 2004 | 6 | 0.67 |
| Yilmaz | 2016 | 4 | 0.44 |
| Yousuf et al. | 2015 | 6 | 0.67 |
| Yousuf et al. | 2017 | 6 | 0.67 |
| Zahur et al. | 2008 | 6 | 0.67 |
| Zahur et al. | 2009 | 7 | 0.78 |
| Zahur et al. | 2011 | 6 | 0.67 |
| Zahur et al. | 2014 | 6 | 0.67 |

**Figure S1:** Frequency of quality category of selected studies
