# Supplementary material for: Peste des petits ruminants (PPR) in Africa and Asia: A systematic review and meta‐analysis of the prevalence in sheep and goats between 1969 and 2018
Source: Vet Med Sci. 2020 Jun 12;6(4):813–33. doi: 10.1002/vms3.300 (PMC7738735; doi:10.1002/vms3.300)
Supplement: Supplementary file 3 — Appendix S3 [file VMS3-6-813-s003.docx]

**Texts S2: Article excluded**

**Individual prevalence data not available**

1. Abera, T., Thangavelu, A., Chandran, N. D. J., & Raja, A. (2014). A SYBR Green I based real time RT-PCR assay for specific detection and quantitation of Peste des petits ruminants virus. BMC veterinary research, 10(1), 22.
2. Abraham, G., & Berhan, A. (2001). The use of antigen-capture enzyme-linked immunosorbent assay (ELISA) for the diagnosis of rinderpest and peste des petits ruminants in Ethiopia. Tropical animal health and production, 33(5), 423-430.
3. Abubakar, M., Arshed, M. J., Zahur, A. B., Ali, Q., & Banyard, A. C. (2012). Natural infection with peste des petits ruminants virus: a pre and post vaccinal assessment following an outbreak scenario. Virus research, 167(1), 43-47.
4. Abubakar, M., Manzoor, S., & Ali, Q. (2015). Evaluating the role of vaccine to combat peste des petits ruminants outbreaks in endemic disease situation. Journal of animal science and technology, 57(1), 2.
5. Al-Naeem, A., Abu Elzein, E. M. E., & Al-Afaleq, A. I. (2000). Epizootiological aspects of peste des petits ruminants and rinderpest in sheep and goats in Saudi Arabia. Rev Sci Tech, 19(3), 855-858.
6. Anderson, J., & McKay, J. A. (1994). The detection of antibodies against peste des petits ruminants virus in cattle, sheep and goats and the possible implications to rinderpest control programmes. Epidemiology & Infection, 112(1), 225-231.
7. Aruni, A. W., Lalitha, P. S., Mohan, A. C., Chitravelu, P., & Anbumani, S. P. (1998). Histopathological study of a natural outbreak of peste des petits ruminants in goats of Tamilnadu. Small Ruminant Research, 28(3), 233-240.
8. Awa, D. N., Njoya, A., & Tama, A. N. (2000). Economics of prophylaxis against peste des petits ruminants and gastrointestinal helminthosis in small ruminants in north Cameroon. Tropical Animal Health and Production, 32(6), 391-403.
9. Ayaz, M. M., Muhammad, G., & Rehman, S. (1997). Pneumo-enteritis syndrome among goats in Dera Ghazi Khan. Pakistan Vet. J, 17(2), 97-99.
10. Baazizi, R., Mahapatra, M., Clarke, B. D., Ait-Oudhia, K., Khelef, D., & Parida, S. (2017). Peste des petits ruminants (PPR): A neglected tropical disease in Maghreb region of North Africa and its threat to Europe. PloS one, 12(4), e0175461.
11. Balamurugan, V., R. Apsana, D. S. N. Raju, S. Abraham, G. Govindaraj, M. Nagalingam, D. Hemadri, B. M. Veeregowda, M. R. Gajendragad, and H. Rahman. "Epidemiological investigation of the peste des petitis ruminants outbreaks in Tumkur district, Karnataka, India." (2014).
12. Balamurugan, V., Sen, A., Saravanan, P., Singh, R. P., Singh, R. K., Rasool, T. J., & Bandyopadhyay, S. K. (2006). One-step multiplex RT-PCR assay for the detection of peste des petits ruminants virus in clinical samples. Veterinary research communications, 30(6), 655-666.
13. Balamurugan, V., Sen, A., Venkatesan, G., Yadav, V., Bhanot, V., Bhanuprakash, V., & Singh, R. K. (2010). Application of Semi‐quantitative M Gene‐Based Hydrolysis Probe (TaqMan) Real‐Time RT‐PCR Assay for the Detection of Peste des petitis ruminants Virus in the Clinical Samples for Investigation into Clinical Prevalence of Disease. Transboundary and emerging diseases, 57(6), 383-395.
14. Balamurugan, V., Sen, A., Venkatesan, G., Yadav, V., Bhanot, V., Bhanuprakash, V., & Singh, R. K. (2012). A rapid and sensitive one step-SYBR green based semi quantitative real time RT-PCR for the detection of peste des petits ruminants virus in the clinical samples. Virologica Sinica, 27(1), 1-9.
15. Balamurugan, V., Sen, A., Venkatesan, G., Yadav, V., Bhanuprakash, V., & Singh, R. K. (2010). Isolation and identification of virulent peste des petits ruminants viruses from PPR outbreaks in India. Tropical animal health and production, 42(6), 1043-1046.
16. Balamurugan, V., Singh, R. P., Saravanan, P., Sen, A., Sarkar, J., Sahay, B., ... & Singh, R. K. (2007). Development of an indirect ELISA for the detection of antibodies against Peste-des-petits-ruminants virus in small ruminants. Veterinary research communications, 31(3), 355-364.
17. Banyard, A. C., Wang, Z., & Parida, S. (2014). Peste des petits ruminants virus, Eastern Asia. Emerging infectious diseases, 20(12), 2176.
18. Bao, J., Li, L., Wang, Z., Barrett, T., Suo, L., Zhao, W., ... & Li, J. (2008). Development of one-step real-time RT-PCR assay for detection and quantitation of peste des petits ruminants virus. Journal of virological methods, 148(1-2), 232-236.
19. Bao, J., Wang, Q., Li, L., Liu, C., Zhang, Z., Li, J., ... & Wang, Z. (2017). Evolutionary dynamics of recent peste des petits ruminants virus epidemic in China during 2013–2014. Virology, 510, 156-164.
20. Batten, C. A., Banyard, A. C., King, D. P., Henstock, M. R., Edwards, L., Sanders, A., ... & Barrett, T. (2011). A real time RT-PCR assay for the specific detection of Peste des petits ruminants virus. Journal of virological methods, 171(2), 401-404.
21. Bhuiyan, A.R., Chowdhury, E.H., Kwiatek, O., Parvin, R., Rahman, M.M., Islam, M.R., Albina, E. and Libeau, G., 2014. Dried fluid spots for peste des petits ruminants virus load evaluation allowing for non-invasive diagnosis and genotyping. BMC veterinary research, 10(1), p.247.
22. Boshra, H., Truong, T., Babiuk, S. and Hemida, M.G., 2015. Seroprevalence of sheep and goat pox, peste des petits ruminants and Rift Valley fever in Saudi Arabia. PLoS One, 10(10), p.e0140328.
23. Boussini, H., Chitsungo, E., Bodjo, S.C., Diakite, A., Nwankpa, N., Elsawalhy, A., Anderson, J.R., Diallo, A. and Dundon, W.G., 2016. First report and characterization of peste des petits ruminants virus in Liberia, West Africa. Tropical animal health and production, 48(7), pp.1503-1507.
24. Cam, Y., Gencay, A., Beyaz, L., Atalay, O., Atasever, A., Ozkul, A. and Kibar, M., 2005. Peste des petits ruminants in a sheep and goat flock in Kayseri province, Turkey. Veterinary record, 157(17), p.523.
25. Cao, Z., Jin, Y., Shen, T., Xu, F. and Li, Y., 2018. Risk factors and distribution for peste des petits ruminants (PPR) in Mainland China. Small Ruminant Research, 162, pp.12-16.
26. Chauhan, H.C., Lambade, P.S., Sen, A., Dadawala, A.I., Ranaware, P.B., Chandel, B., Joshi, D.V., Patel, S.S., Pankaj, K., Shah, N.M. and Kher, H.N., 2011. The use of pathological and histopathological techniques in the diagnosis of peste des petits ruminants in India. Veterinaria italiana, 47(1), pp.41-47.
27. Chazya, R., M’kandawire, E., Muma, J.B., Mwacalimba, K.K., Karimuribo, E & Simuunza, M (2015). Peste des Petits ruminants (ppr) introduction into northern Zambia from Tanzania via live goat consignment: a quantitative risk assessment study. International Journal of Science and Agriculture 2 (7), 1-23
28. Chazya, R., Muma, J.B., Mwacalimba, K.K., Karimuribo, E., Mkandawire, E. and Simuunza, M., 2014. A qualitative assessment of the risk of introducing peste des petits ruminants into northern Zambia from Tanzania. Veterinary medicine international, 2014.
29. Choi, K.S., Nah, J.J., Ko, Y.J., Kang, S.Y. and Jo, N.I., 2005. Rapid competitive enzyme-linked immunosorbent assay for detection of antibodies to peste des petits ruminants virus. Clin. Diagn. Lab. Immunol., 12(4), pp.542-547.
30. Clarke, B., Mahapatra, M., Friedgut, O., Bumbarov, V. and Parida, S., 2017. Persistence of Lineage IV Peste-des-petits ruminants virus within Israel since 1993: An evolutionary perspective. PloS one, 12(5), p.e0177028.
31. Couacy-Hymann, E., Roger, F., Hurard, C., Guillou, J.P., Libeau, G. and Diallo, A., 2002. Rapid and sensitive detection of peste des petits ruminants virus by a polymerase chain reaction assay. Journal of virological methods, 100(1-2), pp.17-25.
32. Das, S., Nath, R., Balamurugan, V., Choudhury, R. and Devi, M., 2015. Haemato-Biochemical analysis of goats naturally infected with peste des petitis ruminants.
33. Dayhum, A., Sharif, M., Eldaghayes, I., Kammon, A., Calistri, P., Danzetta, M.L., Di Sabatino, D., Petrini, A., Ferrari, G., Grazioli, S. and Pezzoni, G., 2018. Sero‐prevalence and epidemiology of peste des petits ruminants in Libya. Transboundary and emerging diseases, 65(1), pp.e48-e54.
34. De Nardi, M., Lamin Saleh, S.M., Batten, C., Oura, C., Di Nardo, A. and Rossi, D., 2012. First evidence of peste des petits ruminants (PPR) virus circulation in Algeria (Sahrawi territories): outbreak investigation and virus lineage identification. Transboundary and emerging diseases, 59(3), pp.214-222.
35. Dhar, P., Sreenivasa, B.P., Barrett, T., Corteyn, M., Singh, R.P. and Bandyopadhyay, S.K., 2002. Recent epidemiology of peste des petits ruminants virus (PPRV). Veterinary microbiology, 88(2), pp.153-159.
36. Diallo, A., Minet, C., Le Goff, C., Berhe, G., Albina, E., Libeau, G. and Barrett, T., 2007. The threat of peste des petits ruminants: progress in vaccine development for disease control. Vaccine, 25(30), pp.5591-5597.
37. Diop, M., Sarr, J. and Libeau, G., 2005. Evaluation of novel diagnostic tools for peste des petits ruminants virus in naturally infected goat herds. Epidemiology & Infection, 133(4), pp.711-717.
38. El Arbi, A.S., El Mamy, A.B., Salami, H., Isselmou, E., Kwiatek, O., Libeau, G., Kane, Y. and Lancelot, R., 2014. Peste des petits ruminants virus, Mauritania. Emerging infectious diseases, 20(2), p.334.
39. El-Yuguda, A.D., Nabia, B., Abubakar, M.B. and Baba, S.S., 2008. Prevalence of PPR cases among Sahel goats presented at the Borno State Veterinary clinic Maiduguri Nigeria from 1996-2005. Nigerian Veterinary Journal, 29(1), pp.25-29.
40. Emikpe, B.O., Jarikre, T.A. and Eyarefe, O.D., 2013. Retrospective study of disease incidence and type of pneumonia in nigerian small ruminants in Ibadan, Nigeria. African Journal of Biomedical Research, 16(2), pp.107-113.
41. Ezeibe, M.C.O., Okoroafor, O.N., Ngene, A.A., Eze, J.I., Eze, I.C. and Ugonabo, J.A.C., 2008. Persistent detection of peste de petits ruminants antigen in the faeces of recovered goats. Tropical animal health and production, 40(7), pp.517-519.
42. FAROOQ, U., KHAN, Q.M. and BARRETT, T., 2008. Molecular based diagnosis of rinderpest and peste des petits ruminants virus in Pakistan. Eye, 2(1), p.3.
43. Farooq, U., Khan, Q.M. and Barrett, T., 2008. Molecular diagnosis of rinderpest and peste des petits ruminants virus using trizol reagent. Pakistan Veterinary Journal, 28, pp.63-67.
44. Folitse, R.D., Amemor, E., REJOICE, E.N., Emikpe, B.O. and Tasiame, W., 2017. Pattern of peste des petits ruminants (PPR) distribution in Ghana (2005-2013). Bulgarian Journal of Veterinary Medicine, 20(1).
45. Forsyth, M.A. and Barrett, T., 1995. Evaluation of polymerase chain reaction for the detection and characterisation of rinderpest and peste des petits ruminants viruses for epidemiological studies. Virus research, 39(2-3), pp.151-163.
46. Gomes, A.R., Byregowda, S.M., Veeregowda, B.M., Rathnamma, D., Chandranaik, B.M., Shivashankar, B.P., Mallinath, K.C. and Balamurugan, V., 2016. Epidemiological investigation of the peste des petits ruminant's outbreaks in Karnataka, India.
47. Govindaraj, G., V., Balamurugan, H., Rahman. 2016. Estimation of economic loss of PPR in sheep and goats in India: An annual incidence based analysis. British Journal of Virology, 3(3s): 77-85.
48. Hegde, R., Gomes, A.R., Muniyellappa, H.K., Byregowda, S.M., Giridhar, P. and Renukaprasad, C., 2009. A short note on peste des petits ruminants in Karnataka, India. Revue scientifique et technique, 28(3), p.1031.
49. Kardjadj, M., Kouidri, B., Metref, D., Luka, P.D. and Ben-Mahdi, M.H., 2016. Abortion and various associated risk factors in small ruminants in Algeria. Preventive veterinary medicine, 123, pp.97-101.
50. Karim A, Bhattacharjee U, Puro K, Shakuntala I, Sanjukta R,Das S, Ghatak S, Sen A (2016). Detection of Peste des petits ruminants virus and goatpox virus from an outbreak in goats with high mortality in Meghalaya state, India, Veterinary World, 9(9): 1025-1027.
51. Karimuribo, E.D., Loomu, P.M., Mellau, L.S.B. and Swai, E.S., 2011. Retrospective study on sero-epidemiology of peste des petits ruminants before its official confirmation in northern Tanzania in 2008. Research Opinions in Animal & Veterinary Sciences.
52. Kataria, A.K., Kataria, N. and Gahlot, A.K., 2007. Large scale outbreaks of peste des petits ruminants in sheep and goats in Thar desert of India. Slovenian Veterinary Research, 44(4), pp.123-132.
53. Kihu, S.M., Gitao, G.C., Bebora, L.C., John, N.M., Wairire, G.G., Maingi, N. and Wahome, R.G., 2015. Economic losses associated with Peste des petits ruminants in Turkana County Kenya. Pastoralism, 5(1), p.9.
54. Kock, R.A., Orynbayev, M.B., Sultankulova, K.T., Strochkov, V.M., Omarova, Z.D., Shalgynbayev, E.K., Rametov, N.M., Sansyzbay, A.R. and Parida, S., 2015. Detection and genetic characterization of lineage IV peste des petits ruminant virus in Kazakhstan. Transboundary and emerging diseases, 62(5), pp.470-479.
55. Krishnamoorthy, P., Govindaraj, G., Shome, B.R. and Rahman, H., 2016. Spatio-Temporal Epidemiological Analysis of Livestock Diseases: A Case of Tamil Nadu State In India.
56. Kulkarni, D.D., Bhikane, A.U., Shaila, M.S., Varalakshmi, P., Apte, M.P. and Narladkar, B.W., 1996. Peste des petits ruminants in goats in India. Veterinary record, 138, pp.187-187.
57. Kumar, N., Chaubey, K.K., Chaudhary, K., Singh, S.V., Sharma, D.K., Gupta, V.K., Mishra, A.K. and Sharma, S., 2013. Isolation, identification and characterization of a Peste des Petits Ruminants virus from an outbreak in Nanakpur, India. Journal of virological methods, 189(2), pp.388-392.
58. Libeau, G., Prehaud, C., Lancelot, R., Colas, F., Guerre, L., Bishop, D.H.L. and Diallo, A., 1995. Development of a competitive ELISA for detecting antibodies to the peste des petits ruminants virus using a recombinant nucleobrotein. Research in veterinary science, 58(1), pp.50-55.
59. Luka, P.D., Erume, J., Mwiine, F.N. and Ayebazibwe, C., 2012. Molecular characterization of peste des petits ruminants virus from the Karamoja region of Uganda (2007-2008). Archives of virology, 157(1), pp.29-35.
60. Luka, P.D., Erume, J., Mwiine, F.N., Ayebazibwe, C. and Shamaki, D., 2011. Molecular characterization and phylogenetic study of peste des petits ruminants viruses from North central States of Nigeria. BMC veterinary research, 7(1), p.32.
61. Ma, J., Jianhua, X., Han, L., Xiang, G., Hao, C. and Hongbin, W., 2017. Spatiotemporal pattern of peste des petits ruminants and its relationship with meteorological factors in China. Preventive veterinary medicine, 147, pp.194-198.
62. Maan, S., Kumar, A., Gupta, A.K., Dalal, A., Chaudhary, D., Gupta, T.K., Bansal, N., Kumar, V., Batra, K., Sindhu, N. and Kumar, A., 2018. Concurrent infection of Bluetongue and Peste‐des‐petits‐ruminants virus in small ruminants in Haryana State of India. Transboundary and emerging diseases, 65(1), pp.235-239.
63. Mahajan, S., Agrawal, R., Kumar, M., Mohan, A. and Pande, N., 2012. Sandwich ELISA based evaluation of clinical samples for Peste des petits ruminants (PPR) virus detection. Small ruminant research, 106(2-3), pp.206-209.
64. Mahajan, S., Agrawal, R., Kumar, M., Mohan, A. and Pande, N., 2013. Comparative evaluation of RT-PCR with sandwich-ELISA for detection of Peste des petits ruminant in sheep and goats. Veterinary World, 6(6), p.288.
65. Malik, Y.S., Singh, D., Chandrashekar, K.M., Shukla, S., Sharma, K., Vaid, N. and Chakravarti, S., 2011. Occurrence of Dual Infection of Peste‐Des‐Petits‐Ruminants and Goatpox in Indigenous Goats of Central India. Transboundary and emerging diseases, 58(3), pp.268-273.
66. Manimaran K, Selvaraj J, Jaisree S, Babu RP, Hemalatha S, Raja A, Roy P. An outbreak of peste des petitis ruminants in sheep and goats at Salem district of Tamil Nadu, India. Indian Journal of Animal Research. 2017 Apr 1;51(2):332-5.
67. Mantip, S., Quan, M., Shamaki, D. and Van Vuuren, M., 2016. Comparison of nucleotide sequences of recent and previous lineages of peste-des-petits-ruminants viruses of sheep and goats in Nigeria. Onderstepoort Journal of Veterinary Research, 83(1), pp.1-10.
68. Mariner, J.C., Jones, B.A., Rich, K.M., Thevasagayam, S., Anderson, J., Jeggo, M., Cai, Y., Peters, A.R. and Roeder, P.L., 2016. The opportunity to eradicate peste des petits ruminants. The Journal of Immunology, 196(9), pp.3499-3506.
69. Misinzo, G., Kgotlele, T., Muse, E.A., Van Doorsselaere, J., Berg, M. and Munir, M., 2015. Peste des petits ruminants virus lineage II and IV From goats in southern Tanzania during an outbreak in 2011. British Journal of Virology, 2(1), p.1.
70. Munir, M., Zohari, S., Saeed, A., Khan, Q.M., Abubakar, M., LeBlanc, N. and Berg, M., 2012. Detection and phylogenetic analysis of peste des petits ruminants virus isolated from outbreaks in Punjab, Pakistan. Transboundary and emerging diseases, 59(1), pp.85-93.
71. Muniraju, M., El Harrak, M., Bao, J., Parthiban, A.B.R., Banyard, A.C., Batten, C. and Parida, S., 2013. Complete genome sequence of a peste des petits ruminants virus recovered from an alpine goat during an outbreak in Morocco in 2008. Genome Announc., 1(3), pp.e00096-13.
72. Muniraju, M., Mahapatra, M., Ayelet, G., Babu, A., Olivier, G., Munir, M., Libeau, G., Batten, C., Banyard, A.C. and Parida, S., 2016. Emergence of lineage IV peste des petits ruminants virus in Ethiopia: complete genome sequence of an Ethiopian isolate 2010. Transboundary and emerging diseases, 63(4), pp.435-442.
73. Murag, S., Kumar, M.S., Kumar, M.C., Sanjukta, R.K., Venkatesha, M.D. and Renukaprasad, C., 2010. An Outbreak of Peste Des Petits Ruminants (PPR) in Small Ruminants. Intas Polivet, 11(2).
74. Muse, E.A., Karimuribo, E.D., Gitao, G.C., Misinzo, G., Mellau, L.S., Msoffe, P.L., Swai, E.S. and Albano, M.O., 2012. Epidemiological investigation into the introduction and factors for spread of Peste des Petits Ruminants, southern Tanzania. Onderstepoort Journal of Veterinary Research, 79(2), pp.49-54.
75. Muse, E.A., Karimuribo, E.D., Gitao, G.C., Misinzo, G., Mellau, L.S., Msoffe, P.L., Swai, E.S. and Albano, M.O., 2012. Epidemiological investigation into the introduction and factors for spread of Peste des Petits Ruminants, southern Tanzania. Onderstepoort Journal of Veterinary Research, 79(2), pp.49-54.
76. Muthuchelvan, Dhanavelu, Ankan De, Bikas Debnath, Dheeraj Choudhary, Gnanavel Venkatesan, Kaushal Kishore Rajak, Shashi Bhusan Sudhakar, Divakar Himadri, Awadh Bihari Pandey, and Satya Parida. "Molecular characterization of peste-des-petits ruminants virus (PPRV) isolated from an outbreak in the Indo-Bangladesh border of Tripura state of North-East India." Veterinary microbiology 174, no. 3-4 (2014): 591-595.
77. Ndamukong, K.J.N., Sewell, M.M.H. and Asanji, M.F., 1989. Disease and mortality in small ruminants in the North West Province of Cameroon. Tropical Animal Health and Production, 21(3), pp.191-196.
78. Njue, S., Saeed, K., Maloo, S., Muchai, J., Biaou, C. and Tetu, K., 2018. Sero-prevalence study to determine the effectiveness of Peste de Petits Ruminants vaccination in Somalia. Pastoralism, 8(1), p.17.
79. Osman, N.A., Ali, A.S. and Fadol, M.A., 2009. Antibody seroprevalences against Peste des Petits Ruminants (PPR) virus in sheep and goats in Sudan. Tropical animal health and production, 41(7), p.1449.
80. Pawaiya, R.V.S., Misra, N., Bhagwan, P.S.K. and Dubey, S.C., 2004. Pathology and distribution of antigen in goats naturally infected with peste des petits ruminants virus. Indian Journal of Animal Sciences, 74(1), pp.35-40.
81. Raj, G.D., Kumar, A.S., Shaila, M.S., Nachimuthu, K. and Palaniswami, K.S., 2003. Molecular epidemiology of peste des petits ruminants viruses from southern India.
82. Roeder, P.L., Abraham, G., Kenfe, G. and Barrett, T., 1994. Peste des petits ruminants in Ethiopian goats. Tropical animal health and production, 26(2), pp.69-73.
83. Roy, P., Vairamuthu, S., Thangavelu, A., Chitradevi, S., Purushothaman, V. and Koteeswaran, A., 2010. An outbreak of Peste des Petits Ruminants Among Thelichery Breed of Goats. International Journal of Applied Research in Veterinary Medicine, 8(3).
84. Salih HA, Elfadil AA, Ganawa E. Case–Control Study of Potential Risk Facrtors Associated With Peste Des Petits Ruminants (PPR)Outbreaks in the Sudan.
85. Sayeed, M.A., Rahman, S.M.A., Alam, J. and Taimur, M.J.F.A., 2005. An economic study on goat diseases in some selected areas of Bangladesh. SAARC Journal of Agriculture, 3, pp.17-28.
86. Selvaraju, G. and Balasubramaniam, G.A., 2013. Epidemiological measures of peste des petits ruminants in small ruminants in Tamil Nadu, India.
87. Selvaraju, G. and Balasubramaniam, G.A., 2014. Temporal trends of peste des petits ruminants, bluetongue and sheep pox in North-West agroclimatic zone of Tamil Nadu. Indian Vet. J, 91(9), pp.39-41.
88. Selvaraju, G., 2014. EPIDEMIOLOGICAL MEASURES OF CAUSAL ASSOCIATION BETWEEN PESTE DES PETITS RUMINANTS (PPR) AND ITS DETERMINANTS IN SMALL RUMINANTS.
89. Selvaraju, G., Dinakaran, A.M. and Balasubramaniam, G.A., 2013. Seroepidemiology of Peste Des Petits Ruminants in Small Ruminants of North-West Agroclimatic Zone of Tamil Nadu.
90. ŞEVİK, M., 2012. Molecular Detection of Peste des petits ruminants virus from different organs/tissues of naturally infected animals. Molecular Microbiology.
91. Shaila, M.S., Shamaki, D., Forsyth, M.A., Diallo, A., Goatley, L., Kitching, R.P. and Barrett, T., 1996. Geographic distribution and epidemiology of peste des petits ruminants viruses. Virus research, 43(2), pp.149-153.
92. Sharma, K.K., Kshirsagar, D.P., Kalyani, I.H., Patel, D.R., Vihol, P.D. and Patel, J.M., 2015. Diagnosis of peste des petits ruminants infection in small ruminants through in-house developed Indirect ELISA: Practical considerations. Veterinary world, 8(4), p.443.
93. Singh, B., Bardhan, D., Verma, M.R., Prasad, S. and Sinha, D.K., 2014. Estimation of economic losses due to Peste de Petits Ruminants in small ruminants in India. Veterinary World, 7(4).
94. Singh, R.P., 2011. Control strategies for peste des petits ruminants in small ruminants of India. Revue Scientifique et Technique-OIE, 30(3), p.879.
95. Stem, C., 1993. An economic analysis of the prevention of peste des petits ruminants in Nigerien goats. Preventive veterinary medicine, 16(2), pp.141-150.
96. Toplu, N., Oguzoglu, T.C. and Albayrak, H., 2012. Dual infection of fetal and neonatal small ruminants with border disease virus and peste des petits ruminants virus (PPRV): neuronal tropism of PPRV as a novel finding. Journal of comparative pathology, 146(4), pp.289-297.
97. Tsegaye, D., Belay, B. and Haile, A., 2013. Prevalence of major goat diseases and mortality of goat in Daro-Labu District of West Hararghe, Eastern Ethiopia. Journal of Scientific and Innovative Research, 2(3), pp.665-672.
98. Ularamu, H.G., Owolodun, O.A., Woma, T.Y., Audu, B.J., Aaron, G.B., Chollom, S.C. and Shamaki, D., 2012. Molecular diagnosis of recent suspected outbreaks of peste des petits ruminants (PPR) in Yola, Adamawa State, Nigeria. African Journal of Biotechnology, 11(5), pp.1158-1162.
99. Ullah, R.W., Zahur, A.B., Irshad, H., Latif, A., Dasti, J.I., Malik, A.R. and Afzal, M., 2015. Detection of Peste des Petits Ruminants Virus genome in fecal material of goats after an outbreak in Punjab Province of Pakistan: A longitudinal study. Sri Lanka Journal of Food and Agriculture, 1(2).
100. Wang, J., Wang, M., Wang, S., Liu, Z., Shen, N., Si, W., Sun, G., Drewe, J.A. and Cai, X., 2015. Peste des petits ruminants virus in Heilongjiang province, China, 2014. Emerging infectious diseases, 21(4), p.677.
101. Wasee Ullah, R., Bin Zahur, A., Latif, A., Iqbal Dasti, J., Irshad, H., Afzal, M., Rasheed, T., Rashid Malik, A. and Qureshi, Z.U.A., 2016. Detection of peste des petits ruminants viral RNA in fecal samples of goats after an outbreak in Punjab province of Pakistan: A longitudinal study. BioMed research international, 2016.
102. Woma, T.Y., Adombi, C.M., Yu, D., Qasim, A.M.M., Sabi, A.A., Maurice, N.A., Olaiya, O.D., Loitsch, A., Bailey, D., Shamaki, D. and Dundon, W.G., 2016. Co‐circulation of Peste‐des‐Petits‐Ruminants Virus Asian lineage IV with Lineage II in Nigeria. Transboundary and emerging diseases, 63(3), pp.235-242.
103. Wu, X., Li, L., Li, J., Liu, C., Wang, Q., Bao, J.Y., Zou, Y., Ren, W., Wang, H., Zhang, Y. and Lv, Y., 2016. Peste des Petits ruminants viruses re‐emerging in China, 2013–2014. Transboundary and emerging diseases, 63(5), pp.e441-e446.
104. Yesilbag, K., Yilmaz, Z., Gölcu, E. and Özkul, A., 2005. Peste des petits ruminants outbreak in western Turkey. The Veterinary Record, 157(9), p.260.

**Comparison of methods/method development (others)**

1. Munir, M., Siddique, M. and Ali, Q., 2009. Comparative efficacy of standard AGID and precipitinogen inhibition test with monoclonal antibodies based competitive ELISA for the serology of Peste des Petits Ruminants in sheep and goats. Tropical animal health and production, 41(3), pp.413-420.
2. Osman, N.A., Ali, A.S. and Fadol, M.A., 2008. Rapid detection of Peste des Petits Ruminants (PPR) virus antigen in Sudan by agar gel precipitation (AGPT) and haemagglutination (HA) Tests. Tropical animal health and production, 40(5), pp.363-368.
3. Raj, G.D., Rajanathan, T.M.C., Kumar, C.S., Ramathilagam, G., Hiremath, G. and Shaila, M.S., 2008. Detection of peste des petits ruminants virus antigen using immunofiltration and antigen-competition ELISA methods. Veterinary microbiology, 129(3-4), pp.246-251.
4. Singh, R.P., Sreenivasa, B.P., Dhar, P., Shah, L.C. and Bandyopadhyay, S.K., 2004. Development of a monoclonal antibody based competitive-ELISA for detection and titration of antibodies to peste des petits ruminants (PPR) virus. Veterinary microbiology, 98(1), pp.3-15.
5. Singh, R.P., Sreenivasa, B.P., Dhar, P. and Bandyopadhyay, S.K., 2004. A sandwich-ELISA for the diagnosis of Peste des petits ruminants (PPR) infection in small ruminants using anti-nucleocapsid protein monoclonal antibody. Archives of virology, 149(11), pp.2155-2170.
6. Wosu, L.O., 1991. Haemagglutination test for diagnosis of peste des petits ruminants disease in goats with samples from live animals. Small Ruminant Research, 5(1-2), pp.169-172.
7. Zhang, G.R., Yu, R.S., Zeng, J.Y., Zhu, Y.M., Dong, S.J., Dunzhu, L., Zhu, S., Duoji, C., Lei, Z.H. and Li, Z., 2013. Development of an epitope-based competitive ELISA for the detection of antibodies against Tibetan peste des petits ruminants virus. Intervirology, 56(1), pp.55-59.
8. Ishag, O.M., Intisar, K.S. and Ali, Y.H., 2014. Detection of antibodies to Peste des petits ruminants virus using passive haemagglutination test and cELISA in the White Nile state-Sudan, comparative study. African Journal of Microbiology Research, 8(38), pp.3475-3481.
9. Foroughi, A., Chaharaein, B., Ownagh, A. and Mardani, K., Comparison of c-ELISA and RT-PCR Methods in Detection of Peste Des Petits Ruminants in Early Stage from Clinical Specimens in Goats and Sheep in Kermanshah Province.
10. Abubakar, M., Ashiq, S., Hussain, Z., Hussain, M., Saleha, S. H., Arshed, M. J., & Bin Zahoor, A. (2011). Comparison of antigen detection methods of peste des petits ruminants virus in clinical samples of small ruminants. Bulg J Vet Med, 14(2), 103-8.
11. Misbah, A., Abubakar, M., Anjum, R., Saleha, S., & Qurban, A. (2009). Prevalence of peste des petits ruminants virus (PPRV) in Mardan, Hangu and Kohat District of Pakistan; Comparative analysis of PPRV suspected serum samples using competitive ELISA (cELISA) and agar gel immunodiffusion (AGID). Veterinary world, 2(3), 89.
12. Bahadar, S., Anjum, A. A., Ahmad, M. D., & Hanif, A. (2009). Isolation and identification of peste des petits ruminants virus by cell culture and immunocapture enzyme linked immunosorbent assay. J Anim Plant Sci, 19, 119-121.
13. Bhuiyan, A.R., Rahman, M.M., Begum, J.A., Islam, M.R. and Chowdhury, E.H., 2012. Comparison of genes as target for molecular diagnosis of peste des petits ruminants in goats. Bangladesh Veterinarian, 29(2), pp.56-62.

**Retro prospective study**

1. Mokhtari, A., Azizi, Z. and Fradonbeh, S.R., 2017. Epidemiological study and spatial modeling of peste des petits ruminants (PPR) in central area of Iran. Revista MVZ Córdoba, 22(2), pp.5899-5909.
2. Mondal SP, Yamage M (2014) A Retrospective Study on the Epidemiology of Anthrax, Foot and Mouth Disease, Haemorrhagic Septicaemia, Peste des Petits Ruminants and Rabies in Bangladesh, 2010-2012. PLoS ONE 9(8): e104435.
3. Okoli, I.C., 2003. Incidence and modulating effects of environmental factors on trypanosomosis, peste des petit ruminants (PPR) and bronchopneumonia of West African dwarf goats in Imo state, Nigeria. Livestock Research for Rural Development, 15(9), pp.112-119.
4. Sarker, Y.A., Miah, A.H., Sharif, N., Himel, M.H., Islam, S., Ray, R.C., Paul, T.K., Islam, M.T. and Sikder, M.H., 2015. A retrospective study of common diseases at veterinary teaching hospital, Bangladesh Agricultural University, Mymensingh. Bangladesh Journal of Veterinary Medicine, 13(2), pp.55-61.

**Experimental**

1. Arslan, H. H., Cenesiz, S., Nisbet, C. E. V. A. T., & Yazici, Z. A. F. E. R. (2007). Serum haptoglobin and amyloid A concentrations and clinical findings in sheep with peste des petits ruminants. BULLETIN-VETERINARY INSTITUTE IN PULAWY, 51(4), 471.
2. Bundza, A., Afshar, A., Dukes, T.W., Myers, D.J., Dulac, G.C. and Becker, S.A., 1988. Experimental peste des petits ruminants (goat plague) in goats and sheep. Canadian Journal of Veterinary Research, 52(1), p.46.
3. Couacy-Hymann, E., Bodjo, S.C., Danho, T., Koffi, M.Y., Libeau, G. and Diallo, A., 2007. Early detection of viral excretion from experimentally infected goats with peste-des-petits ruminants virus. Preventive veterinary medicine, 78(1), pp.85-88.
4. Couacy-Hymann, E., Bodjo, S.C., Tounkara, K., Koffi, Y.M., Ohui, A.H., Danho, T. and Bronsvoort, B.M., 2007. Comparison of two competitive ELISAs for the detection of specific peste-des-petits-ruminant antibodies in sheep and cattle populations. African Journal of Biotechnology, 6(6).
5. Ershaduzzaman, M., Rahman, M.M., Roy, B.K. and Chowdhury, S.A., 2007. Studies on the diseases and mortality pattern of goats under farm conditions and some factors affecting mortality and survival rates in Black Bengal kids. Bangladesh Journal of Veterinary Medicine, pp.71-76.
6. Hussain, M., Afzal, M., Muneer, R., Ashfaque, M. and Haq, E.U., 1998. AN OUTBREAK OF PESTE DES PETrrs RUMINANTS IN GOATS IN REA W ALPINDI.
7. Hussain, M., Irshad, H. and Khan, M.Q., 2008. Laboratory diagnosis of transboundary animal diseases in Pakistan. Transboundary and emerging diseases, 55(5‐6), pp.190-195.
8. Islam, M.R., Shamsuddin, M., Das, P.M. and Dewan, M.L., 2001. An outbreak of peste des petits ruminants in Black Bengal goats in Mymensingh, Bangladesh. The Bangladesh Veterinarian, 18(1), pp.14-19.
9. Martrenchar, A., Zoyem, N., Njoya, A., Tama, A.C.N., Bouchel, D. and Diallo, A., 1999. Field study of a homologous vaccine against peste des petits ruminants in northern Cameroon. Small Ruminant Research, 31(3), pp.277-280.
10. Nanda, Y.P., Chatterjee, A., Purohit, A.K., Diallo, A., Innui, K., Sharma, R.N., Libeau, G., Thevasagayam, J.A., Brüning, A., Kitching, R.P. and Anderson, J., 1996. The isolation of peste des petits ruminants virus from Northern India. Veterinary microbiology, 51(3-4), pp.207-216.
11. Odo, B.I., 2003. Comparative study of some prevalent diseases of ecotype goats reared in southeastern Nigeria. Small Ruminant Research, 50(1-2), pp.203-207.
12. Olugasa, B.O. and Anderson, J.R.N., 2012. Assessment of seroconversion against peste des petits ruminants vaccine among sheep and goats in Buchanan, Liberia. Sokoto Journal of Veterinary Sciences, 10(2), pp.56-60.

**Case report**

1. Islam, A., Singh, A., Islam, M.A. and Majumder, S., 2011. Peste Des Petits Ruminants of Goats, Outbreak and Economic Losses: A Case Study. Biological Sciences-PJSIR, 54(2), pp.76-82.
2. TANWAR, V.K., 2013. AN OUTBREAK OF PESTE DES PETITS RUMINANTS IN A GOAT FLOCK. Haryana Veterinarian, 52.
3. Tariq, A., & Shahzad, A. (2013). Diagnosis of Peste Des Petits in 2 Year Old Buck on The Basis of Clinical Signs and Hematology. International Journal of Molecular Veterinary Research, 3(1).

**Same data set in different publication**

1. Khan, H.A., Siddique, M., Abubakar, M. and Ashraf, M., 2008. The detection of antibody against peste des petits ruminants virus in sheep, goats, cattle and buffaloes. Tropical animal health and production, 40(7), pp.521-527.
2. Munir, M., Siddique, M., Shehzad, A., Zohari, S. and Stahl, K., 2010. Seroprevalence of antibodies to peste des petits ruminants at various governmental livestock farms of Punjab, Pakistan. Asian Journal of Epidemiology, 3(3), pp.183-191.

**Non-English**

1. Bidjeh, K., Bornarel, P., Imadine, M. and Lancelot, R., 1995. Premier isolement au Tchad du virus de la PPR et reproduction expérimentale de la maladie. Revue d’élevage et de médecine vétérinaire des pays tropicaux, 48(4), pp.295-300.

**Full-text not available**

1. Bourdin, P. and Doutre, MP, 1976. The plague of small ruminants in Senegal: new data. Review of Livestock and Veterinary Medicine in Tropical Countries , 29 (3), pp.199-204.
2. Elshemey, T. and Mahmoud, M., 2011. Seroprevalence of antibodies against peste des petits ruminants (PPR) virus in sheep and goat in Kingdom Saudia Arabia. Alexandria Journal of Veterinary Sciences, 32(1), pp.175-182.
3. Emikpe, B.O. and Akpavie, S.O., 2010. The prevalence of antibodies to Peste des petits ruminants virus (PPRV) in goats from rural and urban communities in Ibadan, Nigeria. Bulletin of Animal Health and Production in Africa, 58(2).
4. Harish, B.R., Chandranaik, B.M., Raveendra, H., Venkatesha, M.D. and Renukaprasad, C., 2009. Epidemiology and diagnosis of Peste des petits ruminants in Karnataka. Indian Veterinary Journal, 86(8), pp.773-775.
5. Kamissoko, B., Sidibe, CAK, Niang, M., Samake, K., Traore, A., Diakite, A., Sangare, O., Diallo, A. and Libeau, G., 2013. Serological prevalence of plague of small ruminants of sheep and goats in Mali. Review of Livestock and Veterinary Medicine in Tropical Countries , 66 (1), pp.5-10.
6. Okpeku, M., Nodu, M.B. and Jumbo, C., 2013. Goat management systems and peste des petits ruminant (PPR) incidence in rivers and Bayelsa states, Nigeria. Journal of Agriculture, Forestry and the Social Sciences, 11(2), pp.181-186.
7. Pallav, S., Kaushal, K. and Choudhary, M.K., 2010. Seroprevalance of peste des petits ruminants (PPR) in goats and its effective management. Indian Journal of Veterinary Medicine, 30(2), pp.128-129.
8. Ruhweza, S.P., Ayebazibwe, C., Mwiine, F.N., Muhanguzi, D. and Olaho, W., 2010. Seroprevalence of peste des petits ruminants (PPR) virus antibodies in goats and sheep in north-eastern Uganda. Bulletin of Animal Health and Production in Africa, 58(2).
9. Santhosh, A.K., Raveendra, H., Isloor, S., Gomes, R.A., Rathnamma, D., Byregowda, S.M., Prabhudas, K. and Renukaprasad, C., 2009. Seroprevalence of peste des petits ruminants in organised and unorganised sectors in Karnataka. Indian Veterinary Journal, 86(7), pp.659-660.
