# Supplementary material for: Peste des petits ruminants (PPR) in Africa and Asia: A systematic review and meta‐analysis of the prevalence in sheep and goats between 1969 and 2018
Source: Vet Med Sci. 2020 Jun 12;6(4):813–33. doi: 10.1002/vms3.300 (PMC7738735; doi:10.1002/vms3.300)
Supplement: Supplementary file 4 — Appendix S4 [file VMS3-6-813-s004.docx]

**Texts S3: Articles included**

1. Baazizi R, Ait-Oudhia K, Parida S, Mahapatra M, Khelef D. Peste of Small Ruminants in Algeria: Virus Circulation by Serosurvey Preliminary Results. Egyptian Journal of Sheep and Goat Sciences. 2015;65 2363:1-2.

2. Baazizi R, Khelef D, Hussain T. Peste des petits ruminants in algeria: viral circulation of pprv between 2012 and 2015. Journal of animal and plant sciences. 2017;27 5:1522-7.

3. Kardjadj M, Ben-Mahdi M-H, Luka PD. First serological and molecular evidence of PPRV occurrence in Ghardaïa district, center of Algeria. Tropical animal health and production. 2015;47 7:1279-84.

4. Ahmed SSU. Risk factors for peste des petits ruminants: a hospital basedcase-control study. International Journal of Natural Sciences. 2016;6 1.

5. Shahabuddin Ahmed M, Hasan R, Hossain MA, Uddin F, Rashid H, Talha MH, et al. Clinical Prevalence and Influencing Factors Analysis for the Occurrence of Peste Des Petits Ruminants (PPR) Disease of Goat at Sylhet Region, Bangladesh. 2017.

6. Alam MB, Mahmud T, Khan SA, Islam A, Hai MA, Hassan MM. Occurrence of diseases and disease conditions in cattle and goats at the Upazilla Veterinary Hospital, Debidwar, Comilla. Journal of Advanced Veterinary and Animal Research. 2018;5 2:117-22.

7. Amin MR. Prevalence of common parasitic and infectious diseases of goat at Babugonj upazilla, Barisal, Bangladesh. Asian Journal of Medical and Biological Research. 2015;1 3:449-56.

8. Banik S, Podder S, Samad M, Islam M. Sero-surveillance and immunization in sheep and goats against peste des petits ruminants in Bangladesh. Bangladesh Journal of Veterinary Medicine. 2008;6 2:185-90.

9. Bari MS, Rana EA, Ahaduzzaman M, Al Masud A, Das T, Hasan T. Hemato-biochemical parameters of Pesti-des Petits Ruminants (PPR) affected goats in Chittagong, Bangladesh. Journal of Advanced Veterinary and Animal Research. 2018;5 2:211-7.

10. Bupasha Z, Hossain F, Sarker M, Ahaduzzaman M, Biswas P. Variability in prevalence and therapeutic effectiveness in PPR affected goats of Thakurgoan, Bangladesh. Annals of Veterinary and Animal Science. 2015;2:15-9.

11. Chowdhury EH, Bhuiyan AR, Rahman MM, Siddique MSA, Islam MR. Natural peste des petits ruminants virus infection in Black Bengal goats: virological, pathological and immunohistochemical investigation. BMC veterinary research. 2014;10 1:263.

12. Das KK, Shil NK, Islam MR. Sero-epidemiological investigation on Peste des Petits Ruminants in black Bengal goats. Bangladesh Journal of Microbiology. 2007;24 2:143-5.

13. Anowar A, Nadir E. Sero-Monitoring of Peste Des Petits Ruminants (PPR) antibodies in small and large ruminants in Bangladesh. Journal of Animal and Veterinary advances. 2004;3 7:453-8.

14. Islam M, Khan M, Kader H, Begum M, Asgar M. Prevalence of PPR of goat and their response to antibiotic treatment at Mirzaganj Upazila of Patuakhali Distrtict. Journal of Environmental Science and Natural Resources. 2012;5 2:181-4.

15. Islam K, Ahad A, Mahmood A, Rahman MM, Islam MZ, Bin MH, et al. Prevalence and Clinico–Pathological Features of Peste des Petits Ruminants in Different Breeds of Goats and their Response to Antimicrobials. Journal of Infection and Molecular Biology. 2014;2:43-8.

16. Islam SS, Rao S, Akhter AT, Hossain MM, Islam MR, Islam SS, et al. Investigation of peste des petits ruminants outbreaks in goat farms of Chuadanga District of Bangladesh in 2014. Asian Journal of Medical and Biological Research. 2015;1 3:434-41.

17. Islam MM, Hasan MA, Yousuf MA, Islam UK, Shawan MMAK, Islam MR. Seroprevalence of Peste des Petits Ruminant Virus specific antibody in goats in different regions of Bangladesh. Journal of Advanced Veterinary and Animal Research. 2016;3 2:127-33.

18. Md. Mazedul Islam AHMK, Md Zulfekar Ali. Prevalence of Peste des Petits Ruminants (PPR) in goat in Sylhet District, Bangladesh. International Journal of Biosciences. 2018;13 6:102-8.

19. Jaisree S, Hemalatha S, Muthuramalingam T, Manimaran K, Mahaprabhu R, Gnanaraj PT, et al. Investigation on Outbreak of Peste Des Petits Ruminants (PPR) in an Organized Farm among Tellicherry Breed of Goats﻿. 2017.

20. Kabir M, Reza M, Razi K, Parvez M, Bag M, Mahfuz S. A report on clinical prevalence of diseases and disorders in cattle and goat at the Upazilla Veterinary Hospital, Ulipur, Kurigram. International Journal of Biological Research. 2010;2 11:17-23.

21. Kabir ME, Hossain MM, Ershaduzzaman M, Yousuf MA, Islam MR. Sero-surveillance and sero-monitoring of locally produced PPR vaccine in the field and experimental level. Asian Journal of Medical and Biological Research. 2016;2 1:33-7.

22. Lucky NS, Hossain MK, Roy AC, Haque MM, Uddin AM, Islam MM, et al. A longitudinal study on clinical diseases and disorders of cattle and goats in Sylhet, Bangladesh. Journal of Advanced Veterinary and Animal Research. 2016;3 1:24-37.

23. Meher M, Afrin M, Hassan Z, Alam J. Epidemiological investigation of peste des petits ruminants virus infection in goat with therapeutic managementat at Bera upazila of Pabna in Bangladesh. Progressive Agriculture. 2017;28 2:114-9.

24. Mohanto JK, Hoque MF, Juli MSB, Pramanik SK. Prevalence of peste des petits ruminants (ppr) in goat and their response to antibiotic treatment at gangachara upazila of rangpur district.

25. Nabi R, Hossain MS, Saha S, Alam J, Giasuddin M. Molecular epidemiology of peste des petits ruminants (PPR) in goat. Int J Sci Technol Res. 2018;7:71-12.

26. Nath TC, Bhuiyan MJU, Mamun M, Datta R, Chowdhury S, Hossain M, et al. Common infectious diseases of goats in Chittagong district of Bangladesh. Int J Sci Res Agric Sci. 2014;1:43-9.

27. Naznin M, Ahaduzzaman M, Chowdhury S, Biswas P. Prevalence and clinico-pathological parameters of PPR infected goats and their response to antibiotic treatment at Panchlaish, Chittagong, Bangladesh. International Journal of Natural Sciences. 2014;4 2:1-7.

28. Parvez MA, Khatun R, Al Noman MA. Prevalence and associated risk factors of Peste des Petits Ruminants (PPR) in goats in Chittagong district, Bangladesh. Res J Vet Pract. 2014;2:14-7.

29. Poddar S, Tuli D, Sultana J, Akter S, Alauddin M. Prevalence of Peste des Petits Ruminants in Goat at Upizalla Veterinary Hospital, Pirojpur Sadar, Bangladesh. TURKISH JOURNAL OF VETERINARY RESEARCH. 2 1:5-8.

30. Rahman M, Hossain M, Ahsan M, Khokon M, Kibria A. Prevalence of PPR and its effective treatment in goats of Pabna district of Bangladesh. International Journal of Aquaculture and Fishery Sciences. 2011;4:418-22.

31. Rahman M, Shadmin I, Noor M, Parvin R, Chowdhury E, Islam M. Peste des petits ruminants virus infection of goats in Bangladesh: Pathological investigation, molecular detection and isolation of the virus. Bangladesh Veterinarian. 2011;28 1:1-7.

32. Rahman MM, Alam KJ, Alam MS, Hasan MM, Moonmoon M. A study on prevalence of peste des petits ruminant (PPR) in goat at Bagmara upazilla at Rajshahi district in Bangladesh. Research in Agriculture Livestock and Fisheries. 2016;3 2:339-44.

33. Rahman MM, Hassan MZ, Sultana S, Uddin MK, Hossain SS. Incidence of Peste des Petits Ruminants in Rangpur sadar of Bangladesh. Asian Journal of Medical and Biological Research. 2017;3 4:529-33.

34. Rahman MZ, Haider N, Gurley ES, Ahmed S, Osmani MG, Hossain MB, et al. Epidemiology and genetic characterization of Peste des petits ruminants virus in Bangladesh. Veterinary medicine and science. 2018;4 3:161-71.

35. Rakshit N, Paul A, Amin M, Asaduzzaman M, Sen P, Talukder M. Occurrence and therapeutic response of Peste des petits ruminants (PPR) in goats at the selected southern part of Bangladesh. Wayamba Journal of Animal Science. 2015;7:1239-43.

36. Rony M, Rahman A, Alam M, Dhand N, Ward M. Peste des Petits Ruminants risk factors and space–time clusters in Mymensingh, Bangladesh. Transboundary and emerging diseases. 2017;64 6:2042-8.

37. Sarker S, Islam MH. Prevalence and risk factor assessment of Peste des petits ruminants in goats in Rajshahi, Bangladesh. Veterinary world. 2011;4 12:546.

38. Siddiqui M, Ahasan A, Islam N, Kundu P, Munshi M, Chowdhury E. Peste des Petits Ruminants (PPR) virus antibodies in goats and cattle of the Saint Martin s Island in Bangladesh. Bangladesh Veterinarian. 2014;31 2:55-9.

39. Yousuf MA, Giasuddin M, Islam SS, Islam MR. Management of an outbreak of peste des petits ruminants with antibiotic combined hyperimmune serum therapy. Asian Journal of Medical and Biological Research. 2015;1 2:230-4.

40. Yousuf MA, Rahman MM, Alauddin M, Rahman SB, Islam SS, Islam MR, et al. Sero-surveillance of peste des petits ruminant viral antibody in goats at different areas of Bangladesh. Asian Journal of Medical and Biological Research. 2017;3 3:347-51.

41. Adombi C, Waqas A, Dundon W, Li S, Daojin Y, Kakpo L, et al. Peste des petits ruminants in Benin: Persistence of a single virus genotype in the country for over 42 years. Transboundary and emerging diseases. 2017;64 4:1037-44.

42. Mahamat O, Doungous T, Kebkiba B, Oumar HA, Oussiguéré A, Yacoub AH, et al. Seroprevalence, geographical distribution, and risk factors of peste des petits ruminants in the Republic of Chad. Journal of Advanced Veterinary and Animal Research. 2018;5 4:420-5.

43. Li J, Li L, Wu X, Liu F, Zou Y, Wang Q, et al. Diagnosis of peste des petits ruminants in wild and domestic animals in Xinjiang, China, 2013–2016. Transboundary and emerging diseases. 2017;64 6:e43-e7.

44. Wang Z, Bao J, Wu X, Liu Y, Li L, Liu C, et al. Peste des petits ruminants virus in Tibet, China. Emerging infectious diseases. 2009;15 2:299.

45. Cêtre‐Sossah C, Kwiatek O, Faharoudine A, Soulé M, Moutroifi Y, Vrel M-A, et al. Impact and epidemiological investigations into the incursion and spread of peste des petits ruminants in the Comoros Archipelago: an increased threat to surrounding Islands. Transboundary and emerging diseases. 2016;63 4:452-9.

46. Birindwa BA, George GC, Ntagereka BP, Christopher O, Lilly BC. Mixed infection of peste-des-petits ruminants and Capripox in goats in South Kivu, Democratic Republic of Congo. Journal of Advanced Veterinary and Animal Research. 2017;4 4:348-55.

47. G. Moumin CM, S. Teshale & M. Gezahegne. Seroprevalence and risk factors for peste des petits ruminants in sheep and goats in Djibouti. Revue scientifique et technique. 2018;37 3:1-18.

48. El-Rahim I, Sharawi S, Barakat M, El-Nahas E. An outbreak of peste des petits ruminants in migratory flocks of sheep and goats in Egypt in 2006. Rev Sci Tech. 2010;29 3:655-62.

49. Elhaig MM, Selim A, Mandour AS, Schulz C, Hoffmann B. Prevalence and molecular characterization of peste des petits ruminants virus from Ismailia and Suez, Northeastern Egypt, 2014–2016. Small ruminant research. 2018;169:94-8.

50. Mahmoud MAE-F, Elbayoumy MK, Sedky D, Ahmed S. Serological investigation of some important RNA viruses affecting sheep and goats in Giza and Beni-Suef governorates in Egypt. Veterinary world. 2017;10 10:1161.

51. Soltan MA, Abd-Eldaim MM. Emergence of peste des petits ruminants virus lineage IV in Ismailia Province, Egypt. Infection, Genetics and Evolution. 2014;28:44-7.

52. Oshiek A, Abdelkadir M, Mihreteab B, Mengesha S, Teklay G, Yemane H, et al. Investigating peste des petits ruminants (PPR) in naturally infected goats and sheep in Anseba Region, Eritrea, by reverse transcription polymerase chain reaction (RT-PCR). Tropical animal health and production. 2018;50 4:915-20.

53. Abraham G, Sintayehu A, Libeau G, Albina E, Roger F, Laekemariam Y, et al. Antibody seroprevalences against peste des petits ruminants (PPR) virus in camels, cattle, goats and sheep in Ethiopia. Preventive veterinary medicine. 2005;70 1-2:51-7.

54. Afera B, Hussien D, Amsalu K. Seroprevalence of peste des petits ruminants in goats of southern parts of Tigray region. Global Veterinaria. 2014;12:512-6.

55. Delil F, Asfaw Y, Gebreegziabher B. Prevalence of antibodies to peste des petits ruminants virus before and during outbreaks of the disease in Awash Fentale district, Afar, Ethiopia. Tropical animal health and production. 2012;44 7:1329-30.

56. Faris D, Yilkal A, Berhe G, Kelay B. Seroprevalence and sero-conversion after vaccination against peste des petits ruminants in sheep and goats from Awash Fentale district, Afar, Ethiopia. Preventive veterinary medicine. 2012;103 2-3:157-62.

57. Fentie T, Teshome Y, Ayele B, Molla W, Fenta N, Nigatu S, et al. Sero-epidemiological study of peste des petits ruminants in small ruminants in Amahara region, Ethiopia. Comparative Clinical Pathology. 2018;27 4:1029-36.

58. Gari G, Mekonnen G, Sibhat D, Abebe A, Sahle M, Abie G. Participatory disease surveillance (PDS) of sheep and goats deseases in selected districts of Afar Regional State: Particular focus on Pestes des petit ruminants (PPR) and sheep and goat pox disease (SGP). Ethiopian Veterinary Journal. 2015;19 1:83-105.

59. Gari G, Serda B, Negesa D, Lemma F, Asgedom H. Serological Investigation of Peste Des Petits Ruminants in East Shewa and Arsi Zones, Oromia Region, Ethiopia. Veterinary Medicine International. 2017;2017.

60. Mebrahtu K, Getachew S, Tesfaye T, Sahlu E, Aragaw K. Sero-epidemiological study of peste des petits ruminants (PPR) in sheep and goats under different production systems in South Omo, southern Ethiopia. Small ruminant research. 2018;169:90-3.

61. Megersa B, Biffa D, Belina T, Debela E, Regassa A, Abunna F, et al. Serological investigation of peste des petits ruminants (PPR) in small ruminants managed under pastoral and agro-pastoral systems in Ethiopia. Small Ruminant Research. 2011;97 1-3:134-8.

62. Waret-Szkuta A, Roger F, Chavernac D, Yigezu L, Libeau G, Pfeiffer DU, et al. Peste des Petits Ruminants (PPR) in Ethiopia: Analysis of a national serological survey. BMC Veterinary Research. 2008;4 1:34.

63. Maganga GD, Verrier D, Zerbinati RM, Drosten C, Drexler JF, Leroy EM. Molecular typing of PPRV strains detected during an outbreak in sheep and goats in south-eastern Gabon in 2011. Virology journal. 2013;10 1:82.

64. Goossens B, Osaer S, Kora S, Chandler K, Petrie L, Thevasagayam J, et al. Abattoir survey of sheep and goats in The Gambia. Veterinary record. 1998;142 11:277-81.

65. Ayim-Akonor M, Obese F, Arthur C, Owusu-Ntumy D, Otsyina H. Molecular detection and differentiation of Peste des Petits Ruminant virus and Rinderpest virus in sheep and goats with PPR-like symptoms in Dangme West District of Ghana.

66. Otsyina H, Arthur C, Ayim-Akunnor M, Obese F. Sero-Prevalence of Pestes Des Petits Ruminant (PPR) in Sheep, Goats and Cattle in Ghana. Bulletin of Animal Health and Production in Africa. 2013;61 3:473-9.

67. Balamurugan V, Saravanan P, Sen A, Rajak K, Bhanuprakash V, Krishnamoorthy P, et al. Sero-epidemiological study of peste des petits ruminants in sheep and goats in India between 2003 and 2009. Revue Scientifique et Technique-OIE. 2011;30 3:889.

68. Balamurugan V, Saravanan P, Sen A, Rajak KK, Venkatesan G, Krishnamoorthy P, et al. Prevalence of peste des petits ruminants among sheep and goats in India. Journal of veterinary science. 2012;13 3:279-85.

69. Balamurugan V, Das S, Raju D, Chakravarty I, Nagalingam M, Hemadri D, et al. Prevalence of peste des petits ruminants in goats in North-East India. Virusdisease. 2014;25 4:488-92.

70. Balamurugan V, Krishnamoorthy P, Raju D, Rajak K, Bhanuprakash V, Pandey A, et al. Prevalence of Peste-des-petits-ruminant virus antibodies in cattle, buffaloes, sheep and goats in India. Virusdisease. 2014;25 1:85-90.

71. Begum SS, Mahato G, Sharma P, Sharma K, Hussain M, Das BC, et al. Seroprevalence of peste des petits ruminants in goats in Assam, India. Asian J Anim Vet Adv. 2016;11 3:210-2.

72. Begum S, Mahato G, Muthuchelvan D, Chaudhary D, Hussain M, Nashiruddullah N, et al. Molecular identification of Peste-des-petits-ruminant virus from goats of Assam state of North-East India. 2017.

73. Bhanuprakash V, Saravanan P, Hosamani M, Balamurugan V, Mondal B, Singh RK. Status of sheep sera to bluetongue, peste des petits ruminants and sheep pox in a few northern states of India. Vet Ital. 2008;44 3:527-36.

74. Bhaskar S, Deshmukh V, Chopade N, Rautmare S, Aziz A. Peste Des Petits Ruminants (PPR) outbreak in sheep and goats in Maharashtra: laboratory confirmation by s-ELISA (Mukteshwar) and vero cell culture. Animal Science Reporter. 2011;5 2:64-8.

75. Chauhan H, Dadawala A, Chandel B, Kalyani I, Patel SS, Kher H. Seroprevalence of peste des petits ruminants in small ruminants under different managemental conditions. Indian Journal of Field Veterinarians. 2012;7 3.

76. Chauhan HC, Kher H, Rajak KK, Sen A, Dadawala AI, Chandel BS. Epidemiology and diagnosis of peste des petits ruminants in sheep and goats by Serological, molecular and isolation methods in Gujarat, India. Advanced in Animal and Veterinary Sciences. 2014;2 4:192-8.

77. Chavan V, Digraskar S, Dhonde S, Bedarkar S. Seromonitoring of Peste Des Petits ruminants (PPR) in goats (Capra hircus) of Parbhani region of maharashtra. Veterinary World. 2009;2 8.

78. Pooja C, Jhala M, Kanani A. Incidence of PPR virus in Gujarat by s-ELISA and molecular detection by F, N and H gene based RT-PCR. Royal Veterinary Journal of India. 2009;5 1/2:1-4.

79. De A, Debnath B, Dutta T, Shil S, Bhadouriya S, Chaudhary D, et al. Sero-epide-miology of peste-des-petits-ruminants in goats of Tripura state of North-East India. Adv Anim Vet Sci. 2016;4 5:215-7.

80. Devi M, Das S, Sharma K, Dutta R. Seroprevelance and molecular detection of peste des petits ruminants in goats of Assam. VirusDisease. 2016;27 1:91-7.

81. Haq AA, Santhamani R, Chakravarti S, Yadav AK, Rajak KK, Upmanyu V, et al. Investigation on Peste des Petits Ruminants Outbreak in Goats of Bareilly District of Uttar Pradesh, India. Journal of Immunology and Immunopathology. 2017;19 1:47-54.

82. Hota A, Biswal S, Sahoo N, Rout M, Chaudhary D, Pandey A, et al. Seroprevalence of PPR among Sheep and Goats of Different Agroclimatic Zones of Odisha.

83. M Islam DC, S Das, T Rahman, S Sarma, J Hussain, A Sultana, M Medhi and SB Gogoi. Seroprevalence of Peste Des Petits ruminants in goats of Assam, India. Journal of Entomology and Zoology Studies. 2017;5 5:899-901.

84. Janus A, Tresamol P, Saseendranath M, Vijayakumar K, Pillai UN. Seroprevalence of PPR in goats in Kerala by cELISA. J Vet Anim Sci. 2009;40:15-6.

85. Karam A, Puro K, Das S, Shakuntala I, Sanjukta R, Milton A, et al. Seroprevalence of peste des petits ruminants and bluetongue in goat population of Meghalaya, India. Veterinary world. 2018;11 12:1689.

86. Karlewad V, Bhikane A, Ambore B, Awaz K. Epidemiological observations on peste des petits ruminants in Osmanabadi goats in Maharashtra. Veterinary Practitioner. 2007;8 1:92-3.

87. KRISHNA V, RAO MS, Shaila M. Neutralizing antibodies to peste-des-petits ruminants virus in small ruminants in Andhra Pradesh-A serological survey. The Indian Journal of Animal Sciences. 2001;71 3.

88. Kumar P, Sinha BS, Roy RK, Kumari RR, Kumar A. Peste des petits ruminants in goats: Sero-epidemiological study in middle Indo-Gangetic plains. Indian Journal of Animal Sciences. 2017;87 4:418-21.

89. Mahajan S, Agrawal R, Kumar M, Mohan A, Pande N. Risk of seroconversion to peste des petits ruminants (PPR) and its association with species, sex, age and migration. Small ruminant research. 2012;104 1-3:195-200.

90. Mahajan S, Agrawal R, Kumar M, Mohan A, Pande N. Incidence of Peste des petits ruminants in nomadic sheep and goat of Jammu region. Vet World. 2013;6 7:384-7.

91. Mahender Milind BJ, D.K. Sharma1, Abhishek Gaurav, M.C. Sharma, Prakash aC. Status of Peste Des Petits Ruminants in Small Ruminants of Semi Arid

Regions of Rajasthan. International Journal of Current Microbiology and Applied Sciences. 2018;7 12:1-8.

92. Patil S, Raghavendra A, Gajendragad M, Bhure S, Sengupta P, Tiwari C, et al. Sero-prevalence of Peste-des-Petits ruminants in small ruminants in Karnataka. Indian Veterinary Journal. 2009;86 2:118-9.

93. Raghavendra A, Gajendragad M, Sengupta P, Patil S, Tiwari C, Balumahendiran M, et al. Seroepidemiology of peste des petits ruminants in sheep and goats of southern peninsular India. Revue scientifique et technique. 2008;27 3:861.

94. Chandrahas S, Chandel B, Chauhan H, Dadawala A. Seroprevalence of PPR in sheep and goats of North Gujarat. Indian Journal of Small Ruminants. 2011;17 1:118-21.

95. Saravanan P, Balamurugan V, Sen A, Sarkar J, Sahay B, Rajak K, et al: Mixed infection of peste des petits ruminants and orf on a goat farm in Shahjahanpur, India. British Medical Journal Publishing Group; 2007.

96. Saritha G, Shobhamani B, Sreedevi B. SERO-PREVALENCE OF PESTE DES PETITS RUMINANTS IN PASTORAL SMALL RUMINANTS WITH SPECIAL REFERENCE ON SENSITIVITY TO AGE AND AGRO-CLIMATIC ZONE. Animal Science. 2014;8 3.

97. Saritha G, Shobhamani B, Rajak K, Sreedevi B. Detection and confirmation of PPR virus antigen in sheep and goats by sandwich-ELISA and RT-PCR in Andhra Pradesh, India. Journal of Advanced Veterinary and Animal Research. 2015;2 2:210-2.

98. Sharma C, Shrivastava M, Mehta H, Shukla P. Studies on incidence of peste des petits ruminants in goats of Indore district of Madhya Pradesh. Veterinary Practitioner. 2012;13 1:16-8.

99. Sharma C, Mehta H, Prakash M, Shukla P. Studies on clinico-haemato-biochemical changes in peste des petits ruminants in goats. Veterinary Practitioner. 2012;13 2:322-5.

100. Shukla N, Singh K, Hirpurkar S. Seroprevalence of peste des petits ruminants in sheep in Chhattisgarh. The Indian Journal of Small Ruminants. 2008;14 1:131-2.

101. Singh R, Saravanan P, Sreenivasa B, Singh R, Bandyopadhyay S. Prevalence and distribution of peste des petits ruminants virus infection in small ruminants in India. Rev Sci Tech. 2004;23 3:807-19.

102. Singh S, Jindal N, Nain S, Khokhar R. Seroprevalence of peste des petits ruminants in sheep and goats in and around Haryana state. Haryana Veterinarian. 2006;45:11-4.

103. Singh D, Malik YS, Sharma K, Kuldeep D. Identification and Phylogenetic Analysis of Peste des Petits Ruminants (PPR) Virus Isolates from India. Asian Journal of Animal and Veterinary Advances. 2015;10 8:386-93.

104. Thombare N, Sinha MK. Economic implications of peste des petits ruminants (PPR) disease in sheep and goats: a sample analysis of district Pune, Maharastra. Agricultural Economics Research Review. 2009;22 347-2016-16859:319-22.

105. Muhsen R. Seroepidemiology of PPR in goats in Basrah province. AL-Qadisiyah Journal of Veterinary Medicine Sciences. 2013;12 1:139-43.

106. Al-Majali AM, Hussain NO, Amarin NM, Majok AA. Seroprevalence of, and risk factors for, peste des petits ruminants in sheep and goats in Northern Jordan. Preventive veterinary medicine. 2008;85 1-2:1-8.

107. Lundervold M, Milner-Gulland E, O'callaghan C, Hamblin C, Corteyn A, Macmillan A. A serological survey of ruminant livestock in Kazakhstan during post-Soviet transitions in farming and disease control. Acta Veterinaria Scandinavica. 2004;45 4:211.

108. Kihu SM, Gachohi JM, Ndungu EK, Gitao GC, Bebora LC, John NM, et al. Sero-epidemiology of Peste des petits ruminants virus infection in Turkana County, Kenya. BMC veterinary research. 2015;11 1:87.

109. Yapici O, Bulut O, Avci O, Kale M, Tursumbetov M, Yavru S, et al. First report on seroprevalence of Bluetongue, Border disease and Peste des Petits Ruminants virus infections in sheep in Kyrgyzstan. Indian Journal of Animal Research. 2014;48 5.

110. Almeshay MD, Gusbi A, Eldaghayes I, Mansouri R, Bengoumi M, Dayhum AS. Peste des petits ruminants in Tripoli Region, Lybia. Veterinaria italiana. 2017;53 3:235-42.

111. Ts U, Uuganbayar E, Odbileg R. Sero-surveillance of “Peste des Petits Ruminants” PPR in Mongolia and development of recommendation. Mongolian Journal of Agricultural Sciences. 2016;19 3:22-6.

112. Kwiatek O, Ali YH, Saeed IK, Khalafalla AI, Mohamed OI, Obeida AA, et al. Asian lineage of peste des petits ruminants virus, Africa. Emerging infectious diseases. 2011;17 7:1223.

113. Acharya N, Poudel SP, Acharya KP. Cross-sectional sero-prevalence study of Peste des Petits Ruminants (PPR) in goats of Syangja and Kaski districts of Nepal. VirusDisease. 2018;29 2:173-9.

114. Farougou S, Gagara M, Mensah GA. Prevalence of peste des petits ruminants in the arid zone in the Republic of Niger. Onderstepoort Journal of Veterinary Research. 2013;80 1:01-6.

115. Ameen S, Ajayi J. Studies on influence of seasonality on clinical conditions of small ruminants in Ogbomoso areas of Oyo State. International Journal of Applied Agriculture and Apiculture Research. 2013;9 1-2:18-27.

116. Bello A, Lawal J, Dauda J, Wakil Y, Lekko Y, Mshellia E, et al. Research for peste des petits ruminants (PPR) virus antibodies in goats, sheep and gazelle from Bauchi and Gombe States, north eastern Nigeria. Direct Res J Agric Food Sci. 2016;4 8:193-8.

117. Bello M, Kazeem H, Oladele S, Fatihu M, Tambuwal F, Jibril A. Seroprevalence of peste des petits ruminants among unvaccinated small ruminants in Sokoto State, northwestern Nigeria. Comparative Clinical Pathology. 2018;27 5:1141-6.

118. El-Yuguda A, Chabiri L, Adamu F, Baba S. Peste des petits ruminants virus (PPRV) infection among small ruminants slaughtered at the central abattoir, Maiduguri, Nigeria. Sahel Journal of Veterinary Science. 2010;8 2.

119. El-Yuguda A, Abubakar M, Nabi A, Andrew A, Baba S. Outbreak of peste des petits ruminant in an unvaccinated Sahel goat farm in Maiduguri, Nigeria. African Journal of Biomedical Research. 2009;12 1:83-7.

120. El-Yuguda A-D, Saheed Baba S, Ganiyu Ambali A, Egwu GO. Seroprevalence of peste des petits ruminants among domestic small and large ruminants in the semi-arid region of North-eastern Nigeria. Veterinary World. 2013;6 10.

121. Ezeokoli C, Umoh J, Chineme C, Isitor G, Gyang E. Clinical and epidemiological features of peste des petits ruminants in Sokoto Red goats. Revue d’elevage et de medecine veterinaire des pays tropicaux. 1986;39 3-4:269-73.

122. Lawal A, Lasisi O, Emikpe B, Ogundipe G. Outbreak of Peste Des Petits Ruminants in West African Dwarf Goats in Eruwa, Southwestern Nigeria. Nigerian Veterinary Journal. 2011;32 4.

123. Luther N, Umoh J, Majiyagbe K, Shamaki D, Nwosuh D, Dogo G. Studies on the prevalence of antibodies to peste des petits ruminants virus (PPRV) among goats in Bauchi state. Nigerian Veterinary Journal. 2005;27 1:17-22.

124. Nwobodo H, Ezeifeka G, Ezejiofor C, Onyianta O. Seroprevalence of Peste des petits ruminants among goats and sheep in Enugu State of Nigeria. Animal Health and Production. 2013;61:613-6.

125. Opasina B. Disease constraints on village goat production in southwest Nigeria. Revue d’élevage et de médecine vétérinaire des pays tropicaux. 1985;38 3:284-94.

126. Opasina B, Putt S. Outbreaks of peste des petits ruminants in village goat flocks in Nigeria. Tropical animal health and production. 1985;17 4:219-24.

127. Taylor W, Al Busaidy S, Barrett T. The epidemiology of peste des petits ruminants in the Sultanate of Oman. Veterinary microbiology. 1990;22 4:341-52.

128. Abubakar M, Jamal SM, Hussain M, Ali Q. Incidence of peste des petits ruminants (PPR) virus in sheep and goat as detected by immuno-capture ELISA (Ic ELISA). Small ruminant research. 2008;75 2-3:256-9.

129. Abubakar M, Jamal S, Khan MA, Ali Q. Peste des petits ruminants outbreak in small ruminants of Northern areas of Pakistan. Res J Vete Sci 2008c. 2008;1 1:56-61.

130. Abubakar M, Ali Q, Khan HA. Prevalence and mortality rate of peste des petitis ruminant (PPR): possible association with abortion in goat. Tropical animal health and production. 2008;40 5:317-21.

131. Abubakar M, Jamal SM, Arshed MJ, Hussain M, Ali Q. Peste des petits ruminants virus (PPRV) infection; its association with species, seasonal variations and geography. Tropical animal health and production. 2009;41 7:1197.

132. Abubakar M, Javed Arshed M, Hussain M, Ali Q. Evidence of peste des petits ruminants in serology of sheep and goats from Sindh, Pakistan. Transboundary and emerging diseases. 2011;58 2:152-6.

133. Abubakar M, Manzoor S, Wensman JJ, Torsson E, Qurban A, Munir M. Molecular and epidemiological features of Peste des Petits ruminants outbreak during endemic situation. Hosts and Viruses. 2016;3 4:123.

134. Abubakar M, Zahur AB, Afzal M, Ali Q, Gonzales J. Peste des Petits Ruminants (PPR) in Pakistan: analysis of a national level serological data. Small ruminant research. 2017;155:57-65.

135. Abubakar M, Zahur AB, Naeem K, Khan MA, Qureshi S. Field and molecular epidemiology of peste des petits ruminants in Pakistan. Pakistan Journal of Zoology. 2018;50 2.

136. Ahmad K, Jamal S, Ali Q, Hussain M. An outbreak of peste des petits ruminants (PPR) in a goat flock in Okara, Pakistan. Young. 2005;209 5:10.

137. Anees M, Shabbir MZ, Muhammad K, Nazir J, Shabbir MAB, Wensman JJ, et al. Genetic analysis of peste des petits ruminants virus from Pakistan. BMC veterinary research. 2013;9 1:60.

138. Atta-ur-Rahman MA, Rahman S, Akhtar M, Ullah S. Peste des petits ruminants antigen in mesenteric lymph nodes of goats slaughtered at DI Khan. Pak Vet J. 2004;24 3:159-60.

139. Abubakar M, Rasool MH, Manzoor S, Saqalein M, Rizwan M, Munir M, et al. Evaluation of risk factors for peste des petits ruminants virus in sheep and goats at the Wildlife-Livestock Interface in Punjab Province, Pakistan. BioMed research international. 2016;2016.

140. Durrani AZ, Kamal N, Mehmood N, Shakoori AR. Prevalence of peste des petits ruminants (KATA) in sheep and goats of Punjab. Pakistan Journal of Zoology. 2010;42 3.

141. Jalees MM, Hussain I, Arshad M, Muhammad G, Khan QM, Mahmood MS. Occurrence of peste des petitis ruminants in five districts of Punjab, Pakistan. Pak Vet J. 2013;33 2:165-9.

142. Jalees MM, Hussain I, Arshad M, Mohammad G, Khan QM. Seroprevalence and molecular detection of Peste des Petits Ruminants Virus (PPRV) in different breeds of sheep and goat of Punjab (Pakistan) and its status in gravid animals. Pakistan Journal of Life and Social Sciences. 2016;14:12-7.

143. Khan H, Siddique M, Arshad M, Khan Q, Rehman S. Sero-prevalence of peste des petits ruminants (PPR) virus in sheep and goats in Punjab province of Pakistan. Pakistan veterinary journal. 2007;27 3:109.

144. Khan HA, Siddique M, Abubakar M, Arshad MJ, Hussain M. Prevalence and distribution of peste des petits ruminants virus infection in small ruminants. Small Ruminant Research. 2008;79 2-3:152-7.

145. Khaskheli AA, Khaskheli MI, Khaskheli A, Khaskheli G, Abro R, Barham GS. Clinical Prevalence of Peste Des Petits Ruminants (PPR) Disease in Small Ruminants at the Urban Areas of Hyderabad, Sindh. Journal of Basic and Applied Sciences. 2017;13:281-6.

146. Maitlo A, Ujan J, Ujjan S, Ruk M, Memon B, Mahar A, et al. Screening of peste des petits ruminants virus in a population of district Khairpur, Pakistan. Genetics and molecular research: GMR. 2017;16 3.

147. Mehmood A, Ali Q, Gadahi JA, Malik SA, Shah SI. Detection of Peste des Petits Ruminants (PPR) virus antibodies in sheep and goat populations of the North West Frontier Province (NWFP) of Pakistan by competitive ELISA (cELISA). Veterinary World. 2009;2 9.

148. Munir M, Siddique M, Shehzad A, Zohari S, Stahl K. Seroprevalence of antibodies to peste des petits ruminants at various governmental livestock farms of Punjab, Pakistan. Asian Journal of Epidemiology. 2010;3 3:183-91.

149. Munir M, Shah SH, Shabbir MZ, Berg M. Peste des petits ruminants in Pakistan. J Infect Mol Biol. 2013;1:64-6.

150. Nizamani A, Nizamani Z, Umrani A, Dewani P, Vandiar M, Gandahi J, et al. Prevalence of peste des petits ruminants virus antibodies in small Ruminantsin Sindh, Pakistan. The Journal of Animal & Plant Sciences. 2015;25 6:1515-9.

151. Rashid A, Asim M, Hussain A. Seroprevalence of peste des petits ruminants (PPR) virus in goats, sheep and cattle at livestock production research institute Bahadurnagar Okara. J Anim Plant Sci. 2008;18 4:114-6.

152. Zahur A, Irshad H, Hussain M, Ullah A, Jahangir M, Khan MQ, et al. The epidemiology of peste des petits ruminants in Pakistan. Revue scientifique et technique. 2008;27 3:877.

153. Zahur A, Ullah A, Irshad H, Farooq M, Hussain M, Jahangir M. Epidemiological investigations of a peste des petits ruminants (PPR) outbreak in Afghan sheep in Pakistan. Pakistan Veterinary Journal. 2009;29 4:174-8.

154. Zahur A, Ullah A, Hussain M, Irshad H, Hameed A, Jahangir M, et al. Sero-epidemiology of peste des petits ruminants (PPR) in Pakistan. Preventive Veterinary Medicine. 2011;102 1:87-92.

155. Zahur AB, Ullah A, Irshad H, Latif A, Ullah RW, Jahangir M, et al. Epidemiological analysis of Peste des Petits Ruminants (PPR) outbreaks in Pakistan. Journal of Biosciences and Medicines. 2014;2 06:18.

156. Adel A-A, Abu-Elzein E, Al-Naeem A-M, Amin M. Serosurveillance for peste des petits ruminants (PPR) and rinderpest antibodies in naturally exposed Saudi sheep and goats. Veterinarski arhiv. 2004;74 6:459-65.

157. Al-Dubaib M. Prevalence of Peste despetitis ruminants Infection in Sheep and Goat Farms at the Central Region of Saudi Arabia. Research journal of Veterinary Sciences. 2008;1 1:67-70.

158. Al-Dubaib M. Peste des petitis ruminants morbillivirus infection in lambs and young goats at Qassim region, Saudi Arabia. Tropical animal health and production. 2009;41 2:217-20.

159. El-Rahim I, Baky M, Habashi A, Mahmoud M, Al-Mujalii D. Peste des petits ruminants among sheep and goats in Saudi Arabia in 2004. Assuit Veterinary Medicine Journal. 2005;51:100-11.

160. Mahmoud AZ, Abdellatif M, Shazali L. Prevalence of PPR-virus antibodies in sheep, goats and camels in Hail, Saudi Arabia. British Journal of Virology. 2016;3 3:86.

161. Mahmoud A, Abdellatif M, Abdalla A. High seroprevalence of PPRV-antibodies among sheep and goats in Hail, Saudi Arabia. Vet Sci Res Rev. 2017;3 1:1-5.

162. Mahmoud M, Galbat S. Outbreak of foot and mouth disease and peste des petits ruminants in sheep flock imported for immediate slaughter in Riyadh. Veterinary world. 2017;10 2:238.

163. Sundufu AJ, Ansumana R, Bockarie AS, Bangura U, Lamin JM, Jacobsen KH, et al. Syndromic surveillance of peste des petits ruminants and other animal diseases in Koinadugu district, Sierra Leone, 2011–2012. Tropical animal health and production. 2015;47 2:473-7.

164. Abdalla A, Majok A, El Malik K, Ali A. Sero-prevalence of peste des petits ruminants virus (PPRV) in small ruminants in Blue Nile, Gadaref and North Kordofan States of Sudan. Journal of Public Health and Epidemiology. 2012;4 3:59-64.

165. Ali Y, Intisar K, Khalafalla A. Outbreaks of Peste des petits ruminants in two different localities in Sudan. Journal of Veterinary Medicine and Animal Health. 2014;6 6:174-7.

166. Enan K, Intisar K, Haj M, Hussien M, Taha K, Elfahal A, et al. Seroprevalence of two important viral diseases in small ruminants in Marawi Province Northern State, Sudan. International Journal of Livestock Production. 2013;4 2:18-21.

167. Haroun M, Hajer I, Mukhtar M, Ali B. Detection of antibodies against peste des petits ruminants virus in sera of cattle, camels, sheep and goats in Sudan. Veterinary research communications. 2002;26 7:537-41.

168. Intisar K, Ali YH, Haj M, Sahar M, Shaza M, Baraa A, et al. Peste des petits ruminants infection in domestic ruminants in Sudan. Tropical animal health and production. 2017;49 4:747-54.

169. Ishag O, Intisar K, Ali Y. Detection of antibodies to Peste des petits ruminants virus using passive haemagglutination test and cELISA in the White Nile state-Sudan, comparative study. African Journal of Microbiology Research. 2014;8 38:3475-81.

170. Mostafa SAET. SERO-PREVALENCE AND RISK FACTORS OF PESTE DES PETITS RUMINANT (PPR) IN SHEEP IN RIVER NILE AND WHITE NILE STATES, SUDAN. Journal of Veterinary Medicine and Animal Production. 2013;3 2.

171. Ishag OM, Saeed IK, Ali YH. Peste des petits ruminants outbreaks in White Nile State, Sudan. Onderstepoort Journal of Veterinary Research. 2015;82 1:01-4.

172. Osman NA, Ibrahim HM, Osman AA, Alnour RM, Eldin OAG. Sero-prevalence of peste des petits ruminants virus antibodies in sheep and goats from the Sudan, 2016–2017. VirusDisease. 2018;29 4:531-6.

173. Saeed IK, Ali YH, Khalafalla AI, Rahman-Mahasin E. Current situation of Peste des petits ruminants (PPR) in the Sudan. Tropical animal health and production. 2010;42 1:89.

174. Saeed FA, Abdel-Aziz SA, Gumaa MM. Seroprevalence and Associated Risk Factors of Peste des Petits Ruminants among Sheep and Goats in Kassala State, Sudan. Open Journal of Animal Sciences. 2018;8 04:381.

175. Salih HAM, Elfadil AAM, Saeed IK, Ali YH. Seroprevalence and risk factors of Peste des Petits Ruminants in sheep and goats in Sudan. Journal of Advanced Veterinary and Animal Research. 2014;1 2:42-9.

176. Kgotlele T, Kasanga CJ, Kusiluka LJ, Misinzo G. Preliminary investigation on presence of peste des petits ruminants in Dakawa, Mvomero district, Morogoro region, Tanzania. Onderstepoort Journal of Veterinary Research. 2014;81 2:1-3.

177. Kgotlele T, Macha E, Kasanga C, Kusiluka L, Karimuribo E, Van Doorsselaere J, et al. Partial genetic characterization of peste des petits ruminants virus from goats in northern and eastern Tanzania. Transboundary and emerging diseases. 2014;61:56-62.

178. Kgotlele T, Torsson E, Kasanga C, Wensman JJ, Misinzo G. Seroprevalence of Peste Des Petits Ruminants virus from samples collected in different regions of Tanzania in 2013 and 2015. 2016.

179. Mahapatra M, Sayalel K, Muniraju M, Eblate E, Fyumagwa R, Shilinde S, et al. Spillover of peste des petits ruminants virus from domestic to wild ruminants in the Serengeti ecosystem, Tanzania. Emerging infectious diseases. 2015;21 12:2230.

180. Mbyuzi AO, Komba EV, Kimera SI, Kambarage DM. Sero-prevalence and associated risk factors of peste des petits ruminants and contagious caprine pleuro-pneumonia in goats and sheep in the Southern Zone of Tanzania. Preventive veterinary medicine. 2014;116 1-2:138-44.

181. Matondo R, Muse E, Karimuribo ED, Misinzo G, Albano MO, Gitao GC. Clinico-pathological findings of the 2011 outbreak of peste des petits ruminants (PPR) in Tandahimba district, southern Tanzania. 2012.

182. Swai ES, Kapaga A, Kivaria F, Tinuga D, Joshua G, Sanka P. Prevalence and distribution of Peste des petits ruminants virus antibodies in various districts of Tanzania. Veterinary Research Communications. 2009;33 8:927.

183. Torsson E, Berg M, Misinzo G, Herbe I, Kgotlele T, Päärni M, et al. Seroprevalence and risk factors for peste des petits ruminants and selected differential diagnosis in sheep and goats in Tanzania. Infection ecology & epidemiology. 2017;7 1:1368336.

184. Albayrak H, Alkan F. PPR virus infection on sheep in blacksea region of Turkey: Epidemiology and diagnosis by RT-PCR and virus isolation. Veterinary research communications. 2009;33 3:241-9.

185. Albayrak H, Gür S. A serologic investigation for Peste des petits ruminants infection in sheep, cattle and camels (Camelus dromedarius) in Aydın province, West Anatolia. Tropical animal health and production. 2010;42 2:151-3.

186. Aytekin İ, Mamak N, Ulucan A, Kalınbacak A. Clinical, haematological, biochemical and pathological findings in lambs with peste des petits ruminants. 2011.

187. Güler L, Şevik M, Hasöksüz M. Phylogenetic analysis of peste des petits ruminants virus from outbreaks in Turkey during 2008-2012. Turkish Journal of Biology. 2014;38 5:671-8.

188. Gurcay M, Kizil O, Baydar E. Peste Des Petits Ruminants (PPR) Virus Infections in Goats in the Eastern Anatolia of Turkey. Kafkas Univ Vet Fak Derg. 2013;19:93-8.

189. Özkul A, Akca Y, Alkan F, Barrett T, Karaoglu T, Dagalp SB, et al. Prevalence, distribution, and host range of Peste des petits ruminants virus, Turkey. Emerging infectious diseases. 2002;8 7:709.

190. Ozmen O, Kale M, Haligur M, Yavru S. Pathological, serological, and virological findings in sheep infected simultaneously with Bluetongue, Peste-des-petits-ruminants, and Sheeppox viruses. Tropical animal health and production. 2009;41 6:951-8.

191. Sağlam Y, Temur A. Immunohistochemical detection of peste des petits ruminants (PPR) viral antigen from the cases of naturally occurring pneumonia in sheep. Kafkas Univ Vet Fak Derg. 2009;15 3:423-28.

192. Şevik M, Sait A. Genetic characterization of peste des petits ruminants virus, Turkey, 2009–2013. Research in veterinary science. 2015;101:187-95.

193. Yener Z, Sağlam Y, Temur A, Keleş H. Immunohistochemical detection of peste des petits ruminants viral antigens in tissues from cases of naturally occurring pneumonia in goats. Small Ruminant Research. 2004;51 3:273-7.

194. Yilmaz V. Molecular detection of Peste des Petits Ruminants virus from different sample materials of sheep, Northeastern Turkey. Adv Anim Vet Sci. 2016;4 3:169-73.

195. Mulindwa B, Ruhweza SP, Ayebazibwe C, Mwiine FN, Muhanguzi D, Olaho-Mukani W. Peste des Petits Ruminants serological survey in Karamoja sub region of Uganda by competitive ELISA. infection (Abraham et al, 2005). 2011;2:2.

196. Sande R, Ayebazibwe C, Waiswa C, Ejobi F, Mwiine FN, Olaho-Mukani W. Evidence of peste des petits ruminants virus antibodies in small ruminants in Amuru and Gulu districts, Uganda. Pak Vet J. 2011;31 4:363-5.

**Texts S4:** Characteristics of 196 eligible studies derived from database searches to estimate the pooled prevalence of PPR in sheep and goats

| Reference (Authors and year) | Country | Study duration (month) | Host | Origin of sample | Method of detection | Number | Positive (%) |
| --- | --- | --- | --- | --- | --- | --- | --- |
| (Baazizi et al., 2015) | Algeria | 12 | Sheep | Mixed flock | Serology | 2786 | 485(17.41) |
|  |  |  | Goat | Mixed flock | Serology | 610 | 152(24.92) |
| (Baazizi et al., 2017) | Algeria | 1 | Sheep | Farm | Serology | 2786 | 484(17.37) |
|  |  |  | Goat | Farm | Serology | 610 | 152(24.92) |
| (Kardjadj et al., 2015) | Algeria | 1 | Sheep | Mixed flock | Symptomatic | 282 | 19(6.74) |
|  |  |  | Goat | Mixed flock | Symptomatic | 242 | 45(18.60) |
|  |  |  | Sheep | Mixed flock | Serology | 30 | 6(20) |
|  |  |  | Goat | Mixed flock | Serology | 32 | 11(34.38) |
|  |  |  | Sheep | Mixed flock | PCR | 30 | 6(20) |
|  |  |  | Goat | Mixed flock | PCR | 32 | 8(25) |
| (Ahmed et al., 2016) | Bangladesh | na | Goat | Hospital | Serology | 100 | 35(35) |
| (Ahmed et al., 2017) | Bangladesh | 12 | Goat | Hospital | Symptomatic | 1857 | 336(18.09) |
| (Alam et al., 2018) | Bangladesh | 12 | Goat | Hospital | Symptomatic | 252 | 31(12.30) |
| (Amin, 2015) | Bangladesh | 12 | Goat | Hospital | Symptomatic | 6799 | 580(8.53) |
| (Banik et al., 2008) | Bangladesh | 6 | Sheep | House hold flocks | Serology | 100 | 27(27) |
|  |  |  | Goat | House hold flocks | Serology | 100 | 25(25) |
| (Bari et al., 2018) | Bangladesh | 6 | Goat | Hospital | Symptomatic | 103 | 42(40.78) |
| (Bupasha et al., 2015) | Bangladesh | 2 | Goat | Hospital | Symptomatic | 132 | 52(39.39) |
| (Chowdhury et al., 2014) | Bangladesh | 24 | Goat | Farm | Symptomatic | 1264 | 947(74.92) |
| (Das et al., 2007) | Bangladesh | 1 | Goat | House hold flocks | Serology | 38 | 23(60.53) |
| (Haque et al., 2004) | Bangladesh | 24 | Goat | na | Serology | 750 | 370(49.33) |
| (Islam et al., 2012) | Bangladesh | 3 | Goat | Hospital | Symptomatic | 183 | 92(50.27) |
| (Islam et al., 2014) | Bangladesh | 6 | Goat | Hospital | Symptomatic | 182 | 87(47.80) |
| (Islam et al., 2015) | Bangladesh | 2 | Goat | Farm | Symptomatic | 284 | 44(15.49) |
| (Islam et al., 2016) | Bangladesh | 6 | Goat | Mixed flock | Serology | 414 | 36(8.70) |
| (Islam et al., 2018) | Bangladesh | 36 | Goat | Hospital | Symptomatic | 8975 | 1628(18.14) |
| (Jaisree et al., 2017) | Bangladesh | 1 | Goat | Farm | PCR | 25 | 25(100) |
| (Kabir et al., 2010) | Bangladesh | 3 | Goat | Hospital | Symptomatic | 115 | 33(28.70) |
| (Kabir et al., 2016) | Bangladesh | na | Goat | na | Symptomatic | 240 | 39(16.25) |
| (Lucky et al., 2016) | Bangladesh | 12 | Goat | Hospital | Symptomatic | 222 | 19(8.56) |
| (Meher et al., 2017) | Bangladesh | 6 | Goat | Hospital | Symptomatic | 465 | 253(54.41) |
| (Mohanto et al.) | Bangladesh | 4 | Goat | Hospital | Symptomatic | 975 | 412(42.26) |
| (Nabi et al., 2018) | Bangladesh | 4 | Goat | Hospital | symptomatic | 143 | 29(20.28) |
|  |  |  |  | House hold flocks | PCR | 72 | 24(33.33) |
| (Nath et al., 2014) | Bangladesh | 12 | Goat | Hospital | Symptomatic | 2013 | 228(11.33) |
| (Naznin et al., 2014) | Bangladesh | 3 | Goat | Hospital | Symptomatic | 202 | 98(48.51) |
| (Parvez et al., 2014) | Bangladesh | 24 | Goat | Hospital | symptomatic | 5485 | 493(8.99) |
| (Poddar et al.) | Bangladesh | 12 | Goat | Hospital | symptomatic | 319 | 43(13.48) |
| (Rahman et al., 2011a) | Bangladesh | 9 | Goat | Hospital | symptomatic | 6408 | 140(2.18) |
| (Rahman et al., 2011b) | Bangladesh | 1 | Goat | Farm | Symptomatic | 37 | 19(51.35) |
| (Rahman et al., 2016) | Bangladesh | 2 | Goat | Hospital | symptomatic | 72 | 20(27.78) |
| (Rahman et al., 2017) | Bangladesh | 4 | Sheep | Hospital | symptomatic | 6 | 4(66.67) |
|  |  |  | Goat | Hospital | symptomatic | 230 | 18(7.83) |
| (Rahman et al., 2018) | Bangladesh | 16 | Goat | Hospital | PCR | 539 | 203(37.66) |
| (Rakshit et al., 2015) | Bangladesh | 2 | Goat | Hospital | symptomatic | 91 | 69(75.82) |
| (Rony et al., 2017) | Bangladesh | 108 | Goat | Hospital | symptomatic | 1048 | 380(36.26) |
| (Sarker and Islam, 2011) | Bangladesh | 11 | Goat | Hospital | symptomatic | 627 | 129(20.57) |
| (Siddiqui et al., 2014) | Bangladesh | 12 | Goat | na | Serology | 192 | 72(37.50) |
| (Yousuf et al., 2015) | Bangladesh | 1 | Goat | Farm | PCR | 159 | 72(45.28) |
| (Yousuf et al., 2017) | Bangladesh | 7 | Goat | na | serology | 200 | 56(28) |
| (Adombi et al., 2017) | Benin | 2 | Sheep | Farm | PCR | 6 | 5(83.33) |
|  |  |  | Goat | Farm | PCR | 13 | 11(84.62) |
| (Mahamat et al., 2018) | Chad | 2 | Sheep | Mixed flock | Serology | 1847 | 1055(57.12) |
|  |  |  | Goat | Mixed flock | Serology | 1699 | 821(48.32) |
| (Li et al., 2017) | China | 36 | Sheep | Farm | PCR | 81 | 76(93.83) |
|  |  |  | Goat | Farm | PCR | 15 | 15(100) |
| (Wang et al., 2009) | China | 5 | Sheep | na | serology | 765 | 8(1.05) |
|  |  |  | Goat | na | serology | 771 | 263(34.11) |
| (Cêtre‐Sossah et al., 2016) | Comoros | 4 | Sheep | Farm | Serology | 35 | 0(0) |
|  |  |  | Goat | Farm | Serology | 1013 | 24(2.37) |
| (Birindwa et al., 2017) | Congo | 9 | Goat | Free range flocks | PCR | 150 | 97(64.67) |
| (Moumin et al., 2018) | Djibouti | 9 | Sheep | House hold flocks | Serology | 301 | 8(2.66) |
|  |  |  | Goat | House hold flocks | Serology | 1215 | 83(6.83) |
| (El-Rahim et al., 2010) | Egypt | 6 | Sheep | Free range flocks | Serology | 150 | 80(53.33) |
|  |  |  | Goat | Free range flocks | Serology | 93 | 74(79.57) |
|  |  |  | Sheep | Free range flocks | Symptomatic | 620 | 119(19.19) |
|  |  |  | Goat | Free range flocks | Symptomatic | 343 | 132(38.48) |
| (Elhaig et al., 2018) | Egypt | 30 | Sheep | Mixed flock | Symptomatic | 405 | 197(48.64) |
|  |  |  | Goat | Mixed flock | Symptomatic | 150 | 104(69.33) |
| (Mahmoud et al., 2017b) | Egypt | 6 | Sheep | Mixed flock | Serology | 190 | 126(66.32) |
|  |  |  | Goat | Mixed flock | Serology | 110 | 50(45.45) |
| (Soltan and Abd-Eldaim, 2014) | Egypt | 24 | Sheep | na | symptomatic | 700 | 150(21.43) |
|  |  |  | Goat | na | symptomatic | 15 | 14(93.33) |
| (Oshiek et al., 2018) | Eritrea | 5 | Sheep | Mixed flock | PCR | 3 | 2(66.67) |
|  |  |  | Goat | Mixed flock | PCR | 29 | 10(34.48) |
| (Abraham et al., 2005) | Ethiopia | 1.5 | Sheep | House hold flocks | Serology | 835 | 109(13.05) |
|  |  |  | Goat | House hold flocks | Serology | 442 | 40(9.05) |
| (Afera et al., 2014) | Ethiopia | 10 | Goat | House hold flocks | Serology | 240 | 114(47.50) |
| (Delil et al., 2012) | Ethiopia | 8 | Sheep | Free range flocks | Serology | 360 | 1(0.28) |
|  |  |  | Goat | Free range flocks | Serology | 879 | 20(2.28) |
|  |  |  | Sheep | Free range flocks | Serology | 41 | 3(7.32) |
|  |  |  | Goat | Free range flocks | Serology | 197 | 84(42.64) |
| (Faris et al., 2012) | Ethiopia | 10 | Sheep | House hold flocks | Serology | 360 | 1(0.28) |
|  |  |  | Goat | House hold flocks | Serology | 879 | 20(2.28) |
| (Fentie et al., 2018) | Ethiopia | na | Sheep | Farm | Serology | 329 | 49(14.89) |
|  |  |  | Goat | Farm | Serology | 343 | 74(21.57) |
| (Gari et al., 2015) | Ethiopia | 3 | Sheep | House hold flocks | Serology | 242 | 171(70.66) |
|  |  |  | Goat | House hold flocks | Serology | 258 | 217(84.11) |
| (Gari et al., 2017) | Ethiopia | 5 | Sheep | Mixed flock | Serology | 293 | 149(50.85) |
|  |  |  | Goat | Mixed flock | Serology | 407 | 190(46.68) |
| (Mebrahtu et al., 2018) | Ethiopia | 6 | Sheep | Mixed flock | Serology | 382 | 62(16.23) |
|  |  |  | Goat | Mixed flock | Serology | 512 | 214(41.80) |
| (Megersa et al., 2011) | Ethiopia | 7 | Sheep | Free range flocks | Serology | 251 | 75(29.88) |
|  |  |  | Goat | Free range flocks | serology | 912 | 284(31.14) |
| (Waret-Szkuta et al., 2008) | Ethiopia | 12 | Sheep | na | serology | 4211 | 350(8.31) |
|  |  |  | Goat | na | serology | 4585 | 431(9.40) |
| (Maganga et al., 2013) | Gabon | 1 | Sheep | House hold flocks | PCR | 3 | 3(100) |
|  |  |  | Sheep | House hold flocks | Symptomatic | 92 | 91(98.91) |
|  |  |  | Goat | House hold flocks | Symptomatic | 11 | 2(18.18) |
| (Goossens et al., 1998) | Gambia | 12 | Sheep | Abattoir | Serology | 438 | 217(49.54) |
|  |  |  | Goat | Abattoir | Serology | 1248 | 487(39.02) |
| (Ayim-Akonor et al., 2014) | Ghana | 13 | Sheep | House hold flocks | PCR | 20 | 18(90) |
|  |  |  | Goat | House hold flocks | PCR | 98 | 94(95.92) |
| (Otsyina et al., 2013) | Ghana | 27 | Sheep | House hold flocks | Serology | 1617 | 814(50.34) |
|  |  |  | Goat | House hold flocks | Serology | 1534 | 689(44.92) |
| (Balamurugan et al., 2011) | India | 84 | Sheep | Mixed flock | Serology | 2197 | 901(41.01) |
|  |  |  | Goat | Mixed flock | Serology | 2687 | 1239(46.11) |
| (Balamurugan et al., 2012a) | India | 85 | Sheep | Mixed flock | Serology | 592 | 145(24.49) |
|  |  |  | Goat | Mixed flock | Serology | 912 | 349(38.27) |
| (Balamurugan et al., 2014a) | India | 12 | Goat | Mixed flock | Serology | 391 | 70(17.90) |
| (Balamurugan et al., 2014c) | India | 12 | Sheep | House hold flocks | Serology | 173 | 79(45.66) |
|  |  |  | Goat | House hold flocks | Serology | 288 | 111(38.54) |
| (Begum et al., 2016) | India | 24 | Goat | House hold flocks | Serology | 918 | 121(13.18) |
| (Begum et al., 2017) | India | 12 | Goat | House hold flocks | PCR | 65 | 39(60) |
| (Bhanuprakash et al., 2008) | India | na | Sheep | Mixed flock | Serology | 516 | 215(41.67) |
| (Bhaskar et al., 2011) | India | 4 | Sheep | Farm | Serology | 24 | 16(66.67) |
|  |  |  | Goat | Farm | Serology | 232 | 161(69.40) |
| (Chauhan et al., 2012) | India | na | Sheep | Mixed flock | Serology | 591 | 335(56.68) |
|  |  |  | Goat | Mixed flock | Serology | 379 | 168(44.33) |
| (Chauhan et al., 2014) | India | na | Sheep | na | Serology | 429 | 245(57.11) |
|  |  |  | Goat | na | Serology | 324 | 79(24.38) |
| (Chavan et al., 2009) | India | na | Goat | na | Serology | 854 | 393(46.02) |
| (Choudhary et al., 2009) | India | na | Sheep | Hospital | Serology | 26 | 26(100) |
|  |  |  | Goat | Hospital | Serology | 53 | 49(92.45) |
| (De et al., 2016) | India | 48 | Goat | House hold flocks | Serology | 3454 | 73(2.11) |
| (Devi et al., 2016) | India | na | Goat | na | Serology | 579 | 158(27.29) |
| (Haq et al., 2017) | India | 2 | Goat | Hospital | Serology | 96 | 18(18.75) |
|  |  |  | Goat | Hospital | PCR | 10 | 3(30) |
| (Hota et al.) | India | 7 | Sheep | Mixed flock | Serology | 217 | 97(44.70) |
|  |  |  | Goat | Mixed flock | Serology | 289 | 148(51.21) |
| (M Islam, 2017) | India | 12 | Goat | na | Serology | 456 | 209(45.83) |
| (Janus et al., 2009) | India | na | Goat | na | Serology | 412 | 64(15.53) |
| (Karam et al., 2018) | India | 6 | Goat | House hold flocks | Serology | 598 | 43(7.19) |
| (Karlewad et al., 2007) | India | 12 | Goat | Hospital | Symptomatic | 740 | 435(58.78) |
| (Krishna et al., 2001) | India | na | Sheep | Mixed flock | Serology | 556 | 20(3.60) |
|  |  |  | Goat | Mixed flock | Serology | 116 | 0(0) |
| (Kumar et al., 2017) | India | 36 | Goat | Mixed flock | Serology | 769 | 265(34.46) |
| (Mahajan et al., 2012) | India | 24 | Sheep | Free range flocks | Serology | 108 | 38(35.19) |
|  |  |  | Goat | Free range flocks | Serology | 108 | 35(32.41) |
|  |  |  | Sheep | Free range flocks | serology | 108 | 25(23.15) |
|  |  |  | Goat | Free range flocks | Serology | 108 | 27(25) |
| (Mahajan et al., 2013) | India | 7 | Sheep | Free range flocks | Serology | 13 | 5(38.46) |
|  |  |  | Goat | Free range flocks | Serology | 21 | 14(66.67) |
| (Milind et al., 2018) | India | na | Sheep | House hold flocks | Serology | 128 | 26(20.31) |
|  |  |  | Goat | House hold flocks | Serology | 505 | 24(4.75) |
| (Patil et al., 2009) | India | na | Sheep | Mixed flock | Serology | 1172 | 418(35.67) |
|  |  |  | Goat | Mixed flock | Serology | 1023 | 391(38.22) |
| (Raghavendra et al., 2008) | India | 9 | Sheep | na | Serology | 1492 | 617(41.35) |
|  |  |  | Goat | na | serology | 2068 | 722(34.91) |
| (Sannat et al., 2011) | India | na | Sheep | Free range flocks | Serology | 282 | 139(49.29) |
|  |  |  | Goat | Free range flocks | serology | 723 | 326(45.09) |
| (Saravanan et al., 2007) | India | 1 | Goat | Farm | serology | 10 | 9(90) |
| (Saritha et al., 2014) | India | na | Sheep | Free range flocks | serology | 398 | 260(65.33) |
|  |  |  | Goat | Free range flocks | serology | 405 | 285(70.37) |
| (Saritha et al., 2015) | India | na | Goat | Mixed flock | PCR | 72 | 18(25) |
|  |  |  | Sheep | Mixed flock | PCR | 66 | 20(30.30) |
| (Sharma et al., 2012b) | India | 12 | Goat | Mixed flock | serology | 2412 | 812(33.67) |
| (Sharma et al., 2012a) | India | na | Goat | Mixed flock | Serology | 1068 | 346(32.40) |
| (Shukla et al., 2008) | India | na | Sheep | na | Serology | 90 | 38(42.22) |
| (Singh et al., 2004) | India | 24 | Sheep | Mixed flock | Serology | 1500 | 545(36.33) |
|  |  |  | Goat | Mixed flock | serology | 2907 | 943(32.44) |
| (Singh et al., 2006) | India | 36 | Sheep | Mixed flock | Serology | 357 | 195(54.62) |
|  |  |  | Goat | Mixed flock | Serology | 339 | 117(34.51) |
| (Singh et al., 2015) | India | 12 | Goat | Hospital | Serology | 58 | 28(48.28) |
| (Thombare and Sinha, 2009) | India | na | Sheep | Farm | symptomatic | 6014 | 3187(52.99) |
|  |  |  | Goat | Farm | symptomatic | 340 | 175(51.47) |
| (Muhsen, 2013) | Iraq | 7 | Goat | na | Serology | 1175 | 325(27.66) |
| (Al-Majali et al., 2008) | Jordan | na | Sheep | Mixed flock | Serology | 929 | 270(29.06) |
|  |  |  | Goat | Mixed flock | Serology | 400 | 196(49) |
| (Lundervold et al., 2004) | Kazakhstan | 24 | Sheep | Farm | serology | 542 | 3(0.55) |
|  |  |  | Goat | Farm | Serology | 137 | 1(0.73) |
| (Kihu et al., 2015a) | Kenya | 12 | Sheep | Free range flocks | Serology | 431 | 136(31.55) |
|  |  |  | Goat | Free range flocks | Serology | 538 | 214(39.78) |
| (Yapici et al., 2014) | Kyrgyzstan | na | Sheep | Free range flocks | serology | 655 | 230(35.11) |
| (Almeshay et al., 2017) | Libya | 3 | Sheep | Mixed flock | Serology | 601 | 266(44.26) |
|  |  |  | Goat | Mixed flock | Serology | 120 | 71(59.17) |
| (Undrakhbayar et al., 2016) | Mongolia | 48 | Sheep | na | serology | 1550 | 12(0.77) |
|  |  |  | Goat | na | serology | 400 | 4(1) |
| (Kwiatek et al., 2011) | Morocco | 12 | Sheep | na | PCR | 36 | 16(44.44) |
| (Acharya et al., 2018) | Nepal | 4 | Goat | House hold flocks | Serology | 460 | 380(82.61) |
| (Farougou et al., 2013) | Niger | 4 | Sheep | Mixed flock | Serology | 253 | 105(41.50) |
|  |  |  | Goat | Mixed flock | Serology | 266 | 126(47.37) |
| (Ameen and Ajayi, 2013) | Nigeria | 120 | Sheep | Free range flocks | Symptomatic | 129 | 8(6.20) |
|  |  |  | Goat | Free range flocks | Symptomatic | 758 | 58(7.65) |
| (Bello et al., 2016) | Nigeria | 6 | Sheep | Mixed flock | Serology | 988 | 192(19.43) |
|  |  |  | Goat | Mixed flock | Serology | 3218 | 2374(73.77) |
| (Bello et al., 2018) | Nigeria | na | Sheep | House hold flocks | Serology | 187 | 98(52.41) |
|  |  |  | Goat | House hold flocks | Serology | 246 | 99(40.24) |
| (El-Yuguda et al., 2010) | Nigeria | 2 | Sheep | Abattoir | Serology | 100 | 41(41) |
|  |  |  | Goat | Abattoir | Serology | 114 | 43(37.72) |
| (El-Yuguda et al., 2009) | Nigeria | na | Sheep | Farm | Serology | 20 | 12(60) |
|  |  |  | Goat | Farm | Serology | 113 | 85(75.22) |
| (El-Yuguda et al., 2013) | Nigeria | na | Sheep | Mixed flock | Serology | 1008 | 771(76.49) |
|  |  |  | Goat | Mixed flock | Serology | 1571 | 810(51.56) |
| (Ezeokoli et al., 1986) | Nigeria | 48 | Sheep | Hospital | Symptomatic | 2123 | 8(0.38) |
|  |  |  | Goat | Hospital | Symptomatic | 1042 | 11(1.06) |
| (Lawal et al., 2011) | Nigeria | 3 | Sheep | Hospital | Serology | 5 | 0(0) |
|  |  |  | Goat | Hospital | Serology | 7 | 7(100) |
|  |  |  | Goat | Hospital | Serology | 20 | 15(75) |
| (Luther et al., 2005) | Nigeria | na | Goat | na | Serology | 227 | 56(24.67) |
| (Nwobodo et al., 2013) | Nigeria | 12 | Sheep | Farm | serology | 226 | 78(34.51) |
|  |  |  | Goat | Farm | Serology | 429 | 230(53.61) |
| (Opasina, 1985) | Nigeria | 30 | Goat | House hold flocks | Symptomatic | 275 | 61(22.18) |
| (Opasina and Putt, 1985) | Nigeria | 2 | Goat | House hold flocks | Symptomatic | 144 | 61(42.36) |
| (Taylor et al., 1990) | Oman | 1 | Sheep | House hold flocks | serology | 156 | 37(23.72) |
|  |  |  | Goat | House hold flocks | serology | 568 | 139(24.47) |
| (Abubakar et al., 2008c) | Pakistan | 36 | Sheep | Mixed flock | Serology | 207 | 73(35.27) |
|  |  |  | Goat | Mixed flock | Serology | 220 | 102(46.36) |
| (Abubakar et al., 2008b) | Pakistan | na | Goat | Free range flocks | Serology | 22 | 11(50) |
|  |  |  | Goat | Free range flocks | Serology | 5 | 3(60) |
| (Abubakar et al., 2008a) | Pakistan | na | Goat | Free range flocks | Serology | 110 | 84(76.36) |
| (Abubakar et al., 2009) | Pakistan | 36 | Sheep | na | Serology | 440 | 238(54.09) |
|  |  |  | Goat | na | Serology | 616 | 272(44.16) |
| (Abubakar et al., 2011) | Pakistan | 18 | Sheep | na | Serology | 101 | 50(49.50) |
|  |  |  | Goat | na | Serology | 522 | 294(56.32) |
| (Abubakar et al., 2016) | Pakistan | 1 | Goat | Mixed flock | Symptomatic | 60 | 19(31.67) |
|  |  |  | Sheep | Mixed flock | Symptomatic | 34 | 0(0) |
| (Abubakar et al., 2017) | Pakistan | 12 | Sheep | Mixed flock | Serology | 6113 | 1129(18.47) |
|  |  |  | Goat | Mixed flock | Serology | 13426 | 3217(23.96) |
| (Abubakar et al., 2018) | Pakistan | 36 | Sheep | na | Symptomatic | 2568 | 688(26.79) |
|  |  |  | Goat | na | Symptomatic | 3653 | 1275(34.90) |
| (Ahmad et al., 2005) | Pakistan | 1 | Goat | Farm | Serology | 35 | 31(88.57) |
| (Anees et al., 2013) | Pakistan | na | Goat | Farm | PCR | 32 | 17(53.13) |
| (Atta-ur-Rahman et al., 2004) | Pakistan | 5 | Goat | Abattoir | Immunoelectrophoresis | 250 | 114(45.60) |
| (Aziz-ul-Rahman et al., 2016) | Pakistan | na | Sheep | Farm | Serology | 125 | 14(11.20) |
|  |  |  | Goat | Farm | Serology | 113 | 39(34.51) |
| (Durrani et al., 2010) | Pakistan | 24 | Sheep | na | PCR | 252 | 39(15.48) |
|  |  |  | Goat | na | PCR | 252 | 46(18.25) |
| (Jalees et al., 2013) | Pakistan | 12 | Sheep | Mixed flock | Serology | 309 | 159(51.46) |
|  |  |  | Goat | Mixed flock | Serology | 131 | 61(46.56) |
| (Jalees et al., 2016) | Pakistan | 15 | Sheep | Mixed flock | Serology | 400 | 244(61) |
|  |  |  | Goat | Mixed flock | Serology | 400 | 176(44) |
| (Khan et al., 2007) | Pakistan | 10 | Sheep | House hold flocks | Serology | 232 | 119(51.29) |
|  |  |  | Goat | House hold flocks | Serology | 428 | 167(39.02) |
| (Khan et al., 2008) | Pakistan | 12 | Sheep | House hold flocks | Serology | 338 | 192(56.80) |
|  |  |  | Goat | House hold flocks | Serology | 595 | 287(48.24) |
| (Khaskheli et al., 2017) | Pakistan | 1 | Sheep | Hospital | Symptomatic | 200 | 60(30) |
|  |  |  | Goat | Hospital | Symptomatic | 200 | 70(35) |
| (Maitlo et al., 2017) | Pakistan | na | Goat | House hold flocks | serology | 92 | 59(64.13) |
| (Mehmood et al., 2009) | Pakistan | na | Sheep | na | Serology | 1353 | 337(24.91) |
|  |  |  | Goat | na | Serology | 3195 | 491(15.37) |
| (Munir et al., 2010) | Pakistan | 7 | Sheep | Farm | Serology | 198 | 77(38.89) |
|  |  |  | Goat | Farm | Serology | 82 | 21(25.61) |
| (Munir et al., 2013) | Pakistan | na | Sheep | House hold flocks | PCR | 5 | 2(40) |
|  |  |  | Goat | House hold flocks | PCR | 8 | 2(25) |
| (Nizamani et al., 2015) | Pakistan | 19 | Sheep | na | serology | 1309 | 487(37.20) |
|  |  |  | Goat | na | Serology | 5787 | 2013(34.78) |
| (Rashid et al., 2008) | Pakistan | na | Sheep | Farm | Serology | 80 | 23(28.75) |
|  |  |  | Goat | Farm | Serology | 110 | 91(82.73) |
| (Zahur et al., 2008) | Pakistan | 42 | Sheep | Mixed flock | serology | 319 | 203(63.64) |
|  |  |  | Goat | Mixed flock | serology | 1144 | 893(78.06) |
| (Zahur et al., 2009) | Pakistan | 1 | Sheep | Farm | Symptomatic | 82 | 34(41.46) |
| (Zahur et al., 2011) | Pakistan | 24 | Sheep | Mixed flock | serology | 819 | 293(35.78) |
|  |  |  | Goat | Mixed flock | serology | 1979 | 980(49.52) |
| (Zahur et al., 2014) | Pakistan | 36 | Sheep | Mixed flock | symptomatic | 2367 | 1154(48.75) |
|  |  |  | Goat | Mixed flock | symptomatic | 5666 | 3898(68.80) |
| (Adel et al., 2004) | Saudi Arabia | 6 | Sheep | Farm | Serology | 701 | 22(3.14) |
|  |  |  | Goat | Farm | Serology | 334 | 2(0.60) |
| (Al-Dubaib, 2008) | Saudi Arabia | 7 | Sheep | na | Serology | 992 | 363(36.59) |
|  |  |  | Goat | na | Serology | 962 | 530(55.09) |
| (Al-Dubaib, 2009) | Saudi Arabia | 5 | Sheep | Farm | Serology | 996 | 363(36.45) |
|  |  |  | Goat | Farm | Serology | 962 | 530(55.09) |
| (El-Rahim et al., 2005) | Saudi Arabia | 5 | Sheep | na | Serology | 32 | 30(93.75) |
|  |  |  | Goat | na | Serology | 64 | 49(76.56) |
| (Mahmoud et al., 2016) | Saudi Arabia | 24 | Sheep | Mixed flock | Serology | 211 | 70(33.18) |
|  |  |  | Goat | Mixed flock | Serology | 132 | 83(62.88) |
| (Mahmoud et al., 2017a) | Saudi Arabia | 48 | Sheep | na | Serology | 683 | 406(59.44) |
|  |  |  | Goat | na | Serology | 624 | 470(75.32) |
| (Mahmoud and Galbat, 2017) | Saudi Arabia | 1 | Sheep | Farm | Serology | 50 | 32(64) |
| (Sundufu et al., 2015) | Sierra Leone | 24 | Goat | Mixed flock | symptomatic | 5679 | 1649(29.04) |
| (Abdalla et al., 2012) | Sudan | 17 | Sheep | House hold flocks | Serology | 399 | 251(62.91) |
|  |  |  | Goat | House hold flocks | Serology | 201 | 120(59.70) |
| (Ali et al., 2014) | Sudan | 1 | Sheep | Farm | Serology | 14 | 7(50) |
|  |  |  | Sheep | Farm | Symptomatic | 3500 | 1099(31.40) |
| (Enan et al., 2013) | Sudan | 1 | Sheep | House hold flocks | Serology | 164 | 42(25.61) |
|  |  |  | Goat | House hold flocks | Serology | 90 | 17(18.89) |
| (Haroun et al., 2002) | Sudan | na | Sheep | Farm | Serology | 52 | 27(51.92) |
|  |  |  | Goat | Farm | Serology | 48 | 27(56.25) |
| (Intisar et al., 2017) | Sudan | 60 | Sheep | na | Serology | 7413 | 4976(67.13) |
|  |  |  | Goat | na | Serology | 1459 | 703(48.18) |
| (Ishag et al., 2014) | Sudan | 11 | Sheep | Mixed flock | Serology | 171 | 93(54.39) |
|  |  |  | Goat | Mixed flock | Serology | 38 | 20(52.63) |
| (Kwiatek et al., 2011) | Sudan | 120 | Sheep | na | PCR | 26 | 21(80.77) |
|  |  |  | Goat | na | PCR | 5 | 5(100) |
| (Mostafa, 2012) | Sudan | na | Sheep | na | Serology | 519 | 275(52.99) |
| (Ishag et al., 2015) | Sudan | 24 | Sheep | Free range flocks | serology | 171 | 93(54.39) |
|  |  |  | Goat | Free range flocks | serology | 38 | 20(52.63) |
| (Osman et al., 2018) | Sudan | 24 | Sheep | na | Serology | 258 | 218(84.50) |
|  |  |  | Goat | na | serology | 62 | 41(66.13) |
| (Saeed et al., 2010) | Sudan | 6 | Sheep | na | Serology | 500 | 336(67.20) |
|  |  |  | Goat | na | Serology | 306 | 170(55.56) |
| (Saeed et al., 2018) | Sudan | 4 | Sheep | Mixed flock | Serology | 546 | 372(68.13) |
|  |  |  | Goat | Mixed flock | serology | 372 | 162(43.55) |
| (Salih et al., 2014) | Sudan | 4 | Sheep | na | Serology | 261 | 114(43.68) |
|  |  |  | Goat | na | serology | 219 | 105(47.95) |
| (Kgotlele et al., 2014a) | Tanzania | na | Sheep | na | PCR | 8 | 5(62.50) |
|  |  |  | Goat | na | PCR | 1 | 1(100) |
| (Kgotlele et al., 2014b) | Tanzania | 5 | Goat | Free range flocks | PCR | 71 | 21(29.58) |
| (Kgotlele et al., 2016) | Tanzania | 24 | Sheep | na | Serology | 952 | 240(25.21) |
|  |  |  | Goat | na | Serology | 2886 | 759(26.30) |
| (Mahapatra et al., 2015) | Tanzania | 1 | Sheep | Free range flocks | Serology | 5 | 0(0) |
|  |  |  | Goat | Free range flocks | serology | 5 | 2(40) |
| (Mbyuzi et al., 2014) | Tanzania | 2 | Sheep | Mixed flock | serology | 70 | 25(35.71) |
|  |  |  | Goat | Mixed flock | serology | 434 | 125(28.80) |
| (Muse et al., 2012) | Tanzania | 1 | Sheep | House hold flocks | PCR | 3 | 0(0) |
|  |  |  | Goat | House hold flocks | PCR | 27 | 17(62.96) |
| (Swai et al., 2009) | Tanzania | na | Sheep | Farm | Serology | 657 | 262(39.88) |
|  |  |  | Goat | Farm | Serology | 892 | 442(49.55) |
| (Torsson et al., 2017) | Tanzania | 2 | Sheep | Mixed flock | serology | 238 | 108(45.38) |
|  |  |  | Goat | Mixed flock | serology | 238 | 115(48.32) |
| (Albayrak and Alkan, 2009) | Turkey | 6 | Sheep | Farm | Serology | 892 | 133(14.91) |
|  |  |  | Sheep | Farm | Serology | 57 | 18(31.58) |
| (Albayrak and Gür, 2010) | Turkey | 1 | Sheep | Farm | Serology | 50 | 44(88) |
| (Aytekin et al., 2011) | Turkey | 1 | Sheep | Farm | Serology | 86 | 12(13.95) |
| (Güler et al., 2014) | Turkey | 60 | Sheep | na | PCR | 147 | 51(34.69) |
|  |  |  | Goat | na | PCR | 43 | 14(32.56) |
| (Gurcay et al., 2013) | Turkey | 10 | Goat | na | Serology | 15 | 15(100) |
| (Özkul et al., 2002) | Turkey | 24 | Sheep | Mixed flock | Serology | 1077 | 315(29.25) |
|  |  |  | Goat | Mixed flock | serology | 209 | 42(20.10) |
| (Ozmen et al., 2009) | Turkey | 1 | Sheep | na | Serology | 15 | 5(33.33) |
| (Sağlam and Temur, 2009) | Turkey | 24 | Sheep | Farm | Immunohistochemistry | 70 | 8(11.43) |
| (Şevik and Sait, 2015) | Turkey | 60 | Sheep | na | PCR | 40 | 8(20) |
|  |  |  | Goat | na | PCR | 9 | 5(55.56) |
| (Yener et al., 2004) | Turkey | 5 | Goat | Abattoir | Immunohistochemistry | 42 | 17(40.48) |
| (Yilmaz, 2016) | Turkey | na | Sheep | na | serology | 32 | 23(71.88) |
| (Mulindwa et al., 2011) | Uganda | 2 | Goat | Free range flocks | Serology | 280 | 160(57.14) |
| (Sande et al., 2011) | Uganda | 1 | Sheep | na | Serology | 86 | 8(9.30) |
|  |  |  | Goat | na | serology | 388 | 46(11.86) |
